# Characterization and expression profiles of the B-box gene family during plant growth and under low-nitrogen stress in *Saccharum*

**DOI:** 10.1186/s12864-023-09185-9

**Published:** 2023-02-17

**Authors:** Zilin Wu, Danwen Fu, Xiaoning Gao, Qiaoying Zeng, Xinglong Chen, Jiayun Wu, Nannan Zhang

**Affiliations:** 1 Guangdong Sugarcane Genetic Improvement Engineering Centre, Institute of Nanfan & Seed Industry, Guangdong Academy of Sciences, Guangzhou, 510316 Guangdong China; 2 Zhanjiang Research Center, Institute of Nanfan & Seed Industry, Guangdong Academy of Sciences, Zhanjiang, 524300 Guangdong China

**Keywords:** B-box, Sugarcane, Gene expression, Abiotic stress

## Abstract

**Background:**

B-box (BBX) zinc-finger transcription factors play crucial roles in plant growth, development, and abiotic stress responses. Nevertheless, little information is available on sugarcane (*Saccharum* spp.) *BBX* genes and their expression profiles.

**Results:**

In the present study, we characterized 25 *SsBBX* genes in the *Saccharum spontaneum* genome database. The phylogenetic relationships, gene structures, and expression patterns of these genes during plant growth and under low-nitrogen conditions were systematically analyzed. The *SsBBXs* were divided into five groups based on phylogenetic analysis. The evolutionary analysis further revealed that whole-genome duplications or segmental duplications were the main driving force for the expansion of the *SsBBX* gene family. The expression data suggested that many *BBX* genes (e.g., *SsBBX1* and *SsBBX13*) may be helpful in both plant growth and low-nitrogen stress tolerance.

**Conclusions:**

The results of this study offer new evolutionary insight into the *BBX* family members in how sugarcane grows and responds to stress, which will facilitate their utilization in cultivated sugarcane breeding.

**Supplementary Information:**

The online version contains supplementary material available at 10.1186/s12864-023-09185-9.

## Background

Numerous crucial roles are played by transcription factors (TFs) in various plant biological processes [1], such as developmental regulation, stress responses, and secondary metabolic pathway mediation [2]. Among them, B-box proteins (BBXs) are a type of zinc finger TF that possesses one or two N-terminal B-box domains in the absence or presence of a C-terminal CCT (CONSTANS, CO-like, and TOC1) domain mediating transcriptional regulation, protein interactions, and nuclear transport [3–5]. As a result of the systematic identification and classification of the *BBX* family in *Arabidopsis thaliana*, 32 members can be distinguished into five groups based on the number of B-box domains and the presence of a CCT domain [3]. The *AtBBX* members of Group I and II contain two B-box domains and a CCT domain, whereas the members of Group III contain a single B-box domain and a CCT domain; Group IV contains two B-box domains without the CCT domain, and Group V has only a single B-box domain [3, 4]. To date, *BBX* genes have been well characterized in many plants, such as *Arabidopsis* [3], rice [6], pear [7], pepper [8], tomato [9], and wolfberry [10].

A large number of analyses have reported that *BBX* genes are indispensably involved in the regulation of growth and development, flowering, and response to abiotic stress [4, 11]. In *A. thaliana*, *AtBBX18* plays an adverse role in both photomorphogenesis and thermotolerance [12], *AtBBX20* participates in the brassinolide and light signal pathways [13], and *AtBBX24* is involved in salt stress signaling and root growth [14]. In *Chrysanthemum morifolium*, *CmBBX24* delays flowering and enhances cold and drought tolerance [15]. In *Solanum sogarandinum*, *SsBBX24* is responsive to salt and PEG stress, but not to the absence of low temperature or water [16]. In apple (*Malus domestica*), *MdBBX20* regulates ultraviolet-b and low temperature signaling [17], and *MdBBX37* down-regulates anthocyanin biosynthesis [18]. In Asian pear (*Pyrus pyrifolia*), *PpBBX16* up-regulates light-induced anthocyanin accumulation [19]. In sweet potato (*Ipomoea batatas*), *IbBBX24* promotes tolerance to Fusarium wilt via the jasmonic acid pathway [20].

Cultivated sugarcane (*Saccharum* spp.) is an important sugar and energy crop that is cultivated in tropical and subtropical regions of the world with high economic value [21]. Commercial sugarcane cultivars are derived from interspecific hybridization between octoploid *S. officinarum* (2n = 8x = 80, x = 10) and *S. spontaneum* (2n = 5x - 16x = 40–128; x = 8) [22] with large and complex genomes. However, during the cultivation process, it is susceptible to extreme weather or unfavorable environmental stress. Therefore, it is of great significance to study the molecular mechanism of sugarcane stress resistance and adaptation. Stress is a vital environmental factor that limits plant growth and productivity. Plants have evolved an effective mechanism to cope with environmental stress over time. The nitrogen use efficiency (NUE) of sugarcane genotypes varies considerably, and high NUE varieties are an important issue [23]. Only 20% (or less) of the nitrogen can be obtained from dry biomass in sugarcane during harvest [24]. Therefore, understanding the use of nitrogen is critical for improving NUE through conventional breeding techniques and genetic engineering tools. *BBX* members from several *Poaceae* species including 25 from sorghum (*Sorghum bicolor*), 36 from maize (*Zea mays*), and 30 from rice (*Oryza sativa*) have been retrieved [6, 25]. However, to date, little is known about the members of the *BBX* family in *Saccharum*. The release of the *S. spontaneum* genome provides a platform for genome-wide identification of the *BBX* gene family in sugarcane [26].

In the present study, the gene structures and evolutionary relationships of the *BBX* gene family in wild sugarcane were comprehensively characterized based on *S. spontaneum* genome data. In addition, the transcription profiles of *BBXs* in various tissues, and under low-nitrogen stress were assessed. The results provide new insight into the evolutionary history of *BBXs* in *S. spontaneum* as well as information on the potential biological functions associated with the regulation of plant growth and abiotic stress in sugarcane.

## Materials and methods

### Identification of *BBX* family members in the *Saccharum spontaneum* genome

The genome data of wild sugarcane (*S. spontaneum* cv AP85–441) were retrieved from the Ming laboratory database [26]. A BlastP search of the *S. spontaneum* genome database was performed using the amino acid sequences of the *BBX* genes in *Arabidopsis thaliana* (32), *Oryza sativa* (30), *Sorghum bicolor* (25), and *Zea mays* (36) extracted from Phytozome v13 (https://phytozome-next.jgi.doe.gov/). To verify the identified genes, a Hidden Markov Model (HMM) profile (PF00643) of the B-box conserved domain [27] was downloaded from the Pfam database (http://pfam.xfam.org/), and then all non-redundant protein sequences were checked based on the default settings of HMMER software (v3.2) [28]. Redundant sequences were removed to retain the longest protein sequence. The *BBX* genes were named following the nomenclature scheme proposed by Khanna [3]. Furthermore, the SMART database (http://smart.embl-heidelberg.de) and the NCBI-CDD database (https://www.ncbi.nlm.nih.gov/Structure/bwrpsb/bwrpsb.cgi) were consulted to investigate the domains of the BBX proteins that have been identified. The isoelectric point (pI) and molecular weight (kDa) of the BBX proteins in *S. spontaneum* were estimated using the ExPASy online tool (http://www.expasy.org/tools/). The subcellular localization of sugarcane BBX proteins was calculated using the online tool WoLF PSORT (https://www.genscript.com/wolf-psort.html) and Plant-mPLoc (http://www.csbio.sjtu.edu.cn/bioinf/plant-multi/). A comparison of the BBX proteins between *S. spontaneum* and *S. bicolor* was conducted using BioEdit v7.2.5 [29].

### Phylogenetic and evolution analysis

The protein sequences of BBXs from *S. spontaneum*, *A. thaliana*, *O. sativa*, *S. bicolor*, and *Z. mays* were used for multiple alignment analysis in ClustalW software [30]. The phylogenetic tree was constructed with MEGA X software [31] using the neighbor-joining algorithm. Bootstrap analysis was carried out with 1000 replicates. The phylogenetic trees were visualized with iTOL v6.0 [32]. The tertiary structure model of the SsBBX protein was established by SWISS-MODEL software (https://swissmodel.expasy.org/interactive). The interspecies and intraspecies duplication of *BBX* genes was identified using MCScanX software. The conditions for determining gene duplication events were as follows: (1) the matching length of two gene sequences is greater than 80% of the length of the longer sequence; (2) the similarity of the matching portion of the two gene sequences is greater than 80%; and (3) among closely linked genes, they only participated in one replication event [33]. The association between the number of gene family and a particular genome-wide duplication mode was identified by enrichment analysis using Fisher’s exact test [34]. The Ka and Ks values of duplicated gene pairs were calculated using a described pipeline [35]. To estimate selection pressure, orthologous *BBX* gene pairs between sugarcane and sorghum were compared using the Ka/Ks rate. The divergence time (T) was calculated using the method previously reported [36]. The collinear and tandem relationships as well as the gene-chromosome locations of *BBX* genes were visualized by Circos software v0.69 [37].

### Characteristics of the BBX protein and gene structure

The exon-intron structures of the *S. spontaneum BBX* genes were analyzed using TBtools v1.098 software [38]. The conserved domains and motifs of the *S. spontaneum* BBX protein sequences were determined using the MEME program and the NCBI-CDD online portal. Multiple sequence alignments of the BBX protein were performed using ClustalW (v 2.0) and sequence logos were displayed using the WebLogo platform (https://weblogo.berkeley.edu/logo.cgi). The physical gene-chromosome locations of the *BBXs* in the *Z. mays*, *S. bicolor*, *O. sativa*, and *S. spontaneum* genomes were extracted from the genome annotation. The chromosomal locations of all identified *S. spontaneum* BBX genes were mapped to *O. sativa*, *S. bicolor*, and *Z. mays* chromosomes using MapChart software (Version 2.1) [39]. The circular linkage map of syntenic gene analysis in the *S. spontaneum*, *O. sativa*, *S. bicolor*, and *Z. mays* genome was constructed using TBtools software v1.098 [38].

### Cis-acting element analysis

To predict the cis-acting elements, the DNA sequences 2000 bp upstream of the translation start site were extracted from the genome sequences of *S. spontaneum* [26], and then these sequences were accessed through the PlantCARE program (http://bioinformatics.psb.ugent.be/webtools/plantcare/html/) to identify possible cis-acting elements in the *SsBBX* gene promoter regions [40].

### Subcellular localization analysis

The SsBBX13 ORF was cloned and inserted into the *pBWA(V)HS-GFP* vector by infusion cloning (Table S5). The *pBWA(V)HS-GFP* vector expressing GFP alone was used as a control. The red fluorescent protein mKATE with a nuclear localization signal (NLS, DPKKKRKV) [41], NLS-mKATE, was used as a nuclear marker. Tobacco (*Nicotiana benthamiana*) leaf infiltration was performed according to a previous protocol [42]. *Agrobacterium tumefaciens* strain GV3101 coexpressing SsBBX13-GFP and NLS-mKATE as well as those coexpressing GFP and NLS-mKATE were separately infiltrated into the two halves of a leaf. The leaves were harvested at 48 h postinoculation. Confocal images were acquired on a Zeiss LSM 800 microscope using a Plan-Apochromat 20×/0.8 M27 objective. The 488-, 561-, and 640-nm lasers were used to excite GFP, mKATE, and chlorophyll fluorescence, respectively. Emitted fluorescence was detected by GaAsP-Detector, set to detect 510 nm for GFP, 580 nm for mKATE, and 685 nm for chlorophyll fluorescence.

### Gene expression analysis

The expression profiles of the *BBXs* in sugarcane were analyzed based on previous research with four groups of transcriptome data (different developmental stages and tissues, leaf gradient, circadian rhythm, and low-nitrogen (LN) stress) [43, 44]. RNA preparation, cDNA library construction, and RNA-Seq library sequencing were performed as previously described [45, 46]. The raw transcriptome data were aligned to the reference gene model *S. spontaneum* AP85–441 using Trinity (https://github.com/trinityrnaseq/trinityrnaseq/wiki). Expression levels were calculated and normalized as fragments per kilobase million (FPKM) values as previously described [45, 46]. The heatmaps of gene expression levels were visualized using TBtools v1.098 [38] based on log_2_-transformed (FPKM) data values.

### Plant material cultivation and treatments

To analyze expression patterns, two *Saccharum* species, *S. spontaneum* cultivar ‘SES-208’ (2 *N* = 8× = 64) and *S. officinarum* cultivar ‘LA-Purple’ (2 N = 8× = 64) were grown in the greenhouse of Fujian Agriculture and Forestry University. To study their expression profiles at multiple developmental stages, tissue samples including stems and leaves at the seedling stage, as well as leaf rolls, leaves, internode-3 (upper), internode-6 (central), and internode-9 (bottom) at the pre-mature and mature stages were collected as previously described [43]. To explore the expression profiles of leaf development, the second leaf of SES-208 (11-day-old) and LA-Purple (15-day-old) was divided into 15 segments and 4 regions, and leaf samples including the basal zone (sink tissue), transitional zone (sink-source transition), maturing zone, and mature zone (activated photosynthetic zone with full differentiation) were collected using a previously described procedure [47]. To analyze the expression profile of the circadian rhythm, leaf samples from mature plants of SES-208 and LA-Purple were consistently collected 12 times at a 2-hour interval in the first 24 hours, followed by 7 times at a 4-hour interval in the next 24 hours. The tissues were collected according to a method described previously between 6:00 a.m. on March 2, 2017, and 6:00 a.m. on March 4, 2017 [48].

To determine the expression pattern of sugarcane under low-nitrogen stress, two *Saccharum* hybrid cultivars, YT55 (LN-tolerant) and YT00–236 (LN-sensitive), belonging to sister lines were cultivated in sugarcane breeding bases (Wengyuan, Guangdong Province) of the Institute of Nanfan & Seed Industry, Guangdong Academy of Sciences. Seedlings of 1-month-old YT55 and YT00–236 were transplanted to a greenhouse with a temperature range of 20 to 28 °C and a relative humidity range of 50 to 75% in a normal nitrogen level (7.5 mmol/L) for 20 days and then switched to a nitrogen-deficient nutrient solution (0.1 mmol/L) for starvation treatment according to a previous report [44]. Three biological replicates of the leaves and roots of a half dozen plants in individual pots were snap-frozen in liquid nitrogen at time points of 0 h, 6 h, 12 h, 24 h, 48 h, and 72 h after starvation and stored at − 80 °C until further analysis.

### Validation of *BBX* gene expression levels by RT–qPCR analysis

The expression levels of 2 *BBX* genes (*BBX1* and *BBX13*) were verified at 6 different time points (0 h, 6 h, 12 h, 24 h, 48 h, and 72 h) in the leaves and roots of *Saccharum* hybrid varieties YT55 and YT00–236 under LN conditions by real-time quantitative PCR (RT-qPCR). The total RNA of the collected roots and leaves was extracted using TRIzol Reagent (Invitrogen Technologies, USA) according to the manufacturer’s instructions. A NanoDrop spectrophotometer (Thermo Scientific, USA) was used to measure RNA quality after electrophoresis on a 1% agarose gel. Reverse transcription, RT-qPCR, and relative expression analysis were performed as previously described [46]. To normalize the expression levels, the constitutively expressed *eukaryotic elongation factor 1a* (*eEF-1a*) and *β-actin* genes were used as the reference genes [49]. The relative gene expression level of each gene was calculated using the 2^-ΔΔCt^ method [50]. A total of three biological and three technical replicates were performed for each sample. The primers for quantitative PCR analysis were designed using Primer Premier 5.0 (Premier Biosoft, USA). The primer sequences are cataloged in Table S6. A three-step PCR procedure was conducted with the aid of the 7500 Real-Time PCR System (Applied Biosystems, USA).

## Results

### Identification of *SsBBX* genes in *Saccharum spontaneum*

The Hidden Markov Model (HMM) and the *A. thaliana*, *O. sativa*, *S. bicolor*, and *Z. mays* BBX protein sequences downloaded from the Phytozome v13 database were applied to search the *BBX* family members in *S. spontaneum*. A total of 25 nonredundant *BBXs* were identified from wild sugarcane *S. spontaneum* AP85–441 [26] without considering 69 redundant alleles (Table S2). The 25 nonredundant *BBX* genes were renamed *SsBBX1–25* (Table 1) in terms of the relative linear arrangement on every chromosome and extensive nomenclature. All details of the *SsBBX* family members are listed in Table 1. Each *SsBBX* had a mean of four alleles (range two to six alleles) (Table S2). These *BBXs* were unequally distributed on all eight *S. spontaneum* chromosomes. Whole-length cDNA ranged from 615 to 1497 bp, and their protein translation products varied from 205 (SsBBX4) to 499 (SsBBX7) residues. The predicted isoelectric points (pI) of the *SsBBXs* ranged from 4.31 to 9.90, with a mean of 5.64. The calculated molecular weight (Mw) ranged from 22.56 to 54.94 kDa, with an average value of 35.36 kDa. The BBX proteins lacked transmembrane helical segments (TMHs) except for ZmBBX30 (Table S4). The subcellular localization results of the two online tools are inconsistent. The WoLF PSORT prediction results indicated that the SsBBX proteins were mainly located in the nucleus, followed by the chloroplast and cytoplasm. However, the Plant-mPLoc prediction results showed that all *SsBBX* family members were distributed in the nucleus. The TMH and subcellular localization of BBX proteins in *S. spontaneum* were compatible with those of other orthologous species, suggesting that they may play similar roles (Table S4). Protein sequence alignment of SsBBXs with their orthologs in sorghum revealed that sugarcane and sorghum shared similarities between 80.0 and 97.0%, with a mean of 89.8% (Table 1). The comparisons of SsBBX amino acid sequence alignments revealed the highest degree of similarity (70.0%) between SsBBX9 and SsBBX17 but the least amount of similarity between SsBBX7 and SsBBX16 (5.2%) (Table S1). These results indicated that a few members of the *SsBBX* family showed distinct functional diversification during evolution, whereas most others exhibited very little functional diversity.

**Table 1:** The characteristics of *BBX* family members in *Saccharum*

Gene name	Gene ID	Chr^a^	CDS^b^	AA^c^	Mw^d^	pI^e^	PL^f^	PL^g^	*Sorghum bicolor*	*Sorghum* ortholog ID	Similarity^h^
SsBBX1	Sspon.01G0026670-1A	1	1266	422	46.32	6.05	Chlo: 10, Nucl: 3	Nucl	SbBBX1	Sobic.001G118100	92.6
SsBBX2	Sspon.01G0039310-1B	1	1233	411	43.96	5.19	Mito: 9, Chlo: 3, Nucl: 1	Nucl	SbBBX2	Sobic.001G372700	93.9
SsBBX3	Sspon.02G0000150-1A	2	1236	412	43.00	5.00	Cyto: 6, Nucl: 3, Mito: 2	Nucl	SbBBX4	Sobic.002G408500	84.2
SsBBX4	Sspon.02G0036490-1B	2	615	205	22.56	5.38	Cyto: 8, Chlo: 3, Nucl: 1	Nucl	SbBBX3	Sobic.002G273300	93.3
SsBBX5	Sspon.03G0036880-1B	3	1077	359	37.07	5.31	Nucl: 11, Cyto: 2	Nucl	SbBBX5	Sobic.003G026700	90.8
SsBBX6	Sspon.04G0004240-2C	4	1446	482	52.01	5.74	Chlo: 10, Nucl: 4	Nucl	SbBBX9	Sobic.004G249500	91.2
SsBBX7	Sspon.04G0004590-2B	4	1497	499	54.94	8.23	Chlo: 10.5, Mito: 1.5	Nucl	SbBBX11	Sobic.004G256200	96.3
SsBBX8	Sspon.04G0007300-1A	4	855	285	29.53	5.53	Nucl: 13	Nucl	SbBBX12	Sobic.004G301000	84.6
SsBBX9	Sspon.04G0007300-1P	5	882	294	31.41	5.19	Nucl: 12, Chlo: 1	Nucl	/	/	
SsBBX10	Sspon.04G0008520-1A	4	993	331	34.88	5.06	Cyto: 7, Chlo: 3, Mito: 2	Nucl	SbBBX8	Sobic.004G211200	97.0
SsBBX11	Sspon.04G0008520-1P	5	978	326	34.38	5.43	Chlo: 3, Mito: 3, Cyto: 2	Nucl	SbBBX14	Sobic.006G135100	94.5
SsBBX12	Sspon.04G0017060-1A	4	1047	349	36.06	6.25	Chlo: 7, Cyto: 7	Nucl	SbBBX6	Sobic.004G063200	83.0
SsBBX13	Sspon.04G0017060-1P	8	1110	370	39.43	5.86	Cyto: 10, Chlo: 3	Nucl	SbBBX24	Sobic.010G214000	92.0
SsBBX14	Sspon.04G0028530-1B	4	738	246	24.30	9.90	Chlo: 9, Nucl: 3, Mito: 2	Nucl	/	/	
SsBBX15	Sspon.05G0007690-2C	5	732	244	25.58	5.04	Cyto: 7, Nucl: 6	Nucl	SbBBX13	Sobic.006G131800	86.5
SsBBX16	Sspon.05G0007690-1P	4	729	243	26.00	5.23	Mito: 4, Chlo: 3, Cyto: 3	Nucl	SbBBX7	Sobic.004G208400	90.1
SsBBX17	Sspon.05G0024260-1B	5	687	229	24.70	4.72	Nucl: 12, Chlo: 2	Nucl	SbBBX15	Sobic.006G163100	86.6
SsBBX18	Sspon.06G0002910-2B	6	1485	495	52.29	6.32	Nucl: 8, Cysk: 3, Cyto: 2	Nucl	SbBBX17	Sobic.007G189800	91.3
SsBBX19	Sspon.06G0008250-1A	6	894	298	29.70	5.71	Plas: 4.5, E.R.: 3.5, Chlo: 3	Nucl	/	/	
SsBBX20	Sspon.06G0023380-1B	6	786	262	26.38	4.63	Nucl: 11, Chlo: 1, Cyto: 1	Nucl	SbBBX16	Sobic.007G062100	87.8
SsBBX21	Sspon.07G0011100-1A	7	984	328	34.12	5.07	Nucl: 5, Mito: 4, Cyto: 3	Nucl	SbBBX19	Sobic.009G075600	93.2
SsBBX22	Sspon.07G0020490-1A	7	669	223	22.74	5.90	Cyto: 10, Nucl: 3	Nucl	SbBBX18	Sobic.008G073400	80.0
SsBBX23	Sspon.08G0001580-1A	8	924	308	31.73	4.77	Pero: 5, Nucl: 3, Extr: 3	Nucl	SbBBX25	Sobic.010G262200	83.8
SsBBX24	Sspon.08G0009590-1A	8	900	300	32.12	4.31	Nucl: 6, Chlo: 5, Cyto: 2	Nucl	SbBBX23	Sobic.010G123500	94.8
SsBBX25	Sspon.08G0021500-2C	8	1371	457	48.74	5.26	Chlo: 13	Nucl	SbBBX21	Sobic.010G108500	87.6

*Chlo* Chloroplast, *Nucl* Nucleus, *Mito* mitochondria, *Cyto* Cytoplasm, *Cysk* Cytoskeleton, *E.R.* Endoplasmic reticulum, *Plas* plasma membrane, *Pero* peroxisome, *Extr* Extracellular. Test k used for kNN is 14

^a^Chromosomal position of the BBXs

^b^Length of coding sequence in *BBX* genes

^c^Amino acid number in BBX protein sequences

^d^Molecular weight (Mw, kDa) predicted by ExPASy (https://web.expasy.org/compute_pi/)

^e^Isoelectric point (pI) predicted by ExPASy (https://web.expasy.org/compute_pi/)

^f^Subcellular location of the BBX proteins predicted by WoLF PSORT (https://www.genscript.com/wolf-psort.html)

^g^Subcellular location of the BBX proteins predicted by Plant-mPLoc (http://www.csbio.sjtu.edu.cn/bioinf/plant-multi/)

^h^Protein sequence similarity (%) between sorghum and sugarcane calculated by BioEdit software

### Subcellular localization of the SsBBX13 protein

**Fig. 1:**
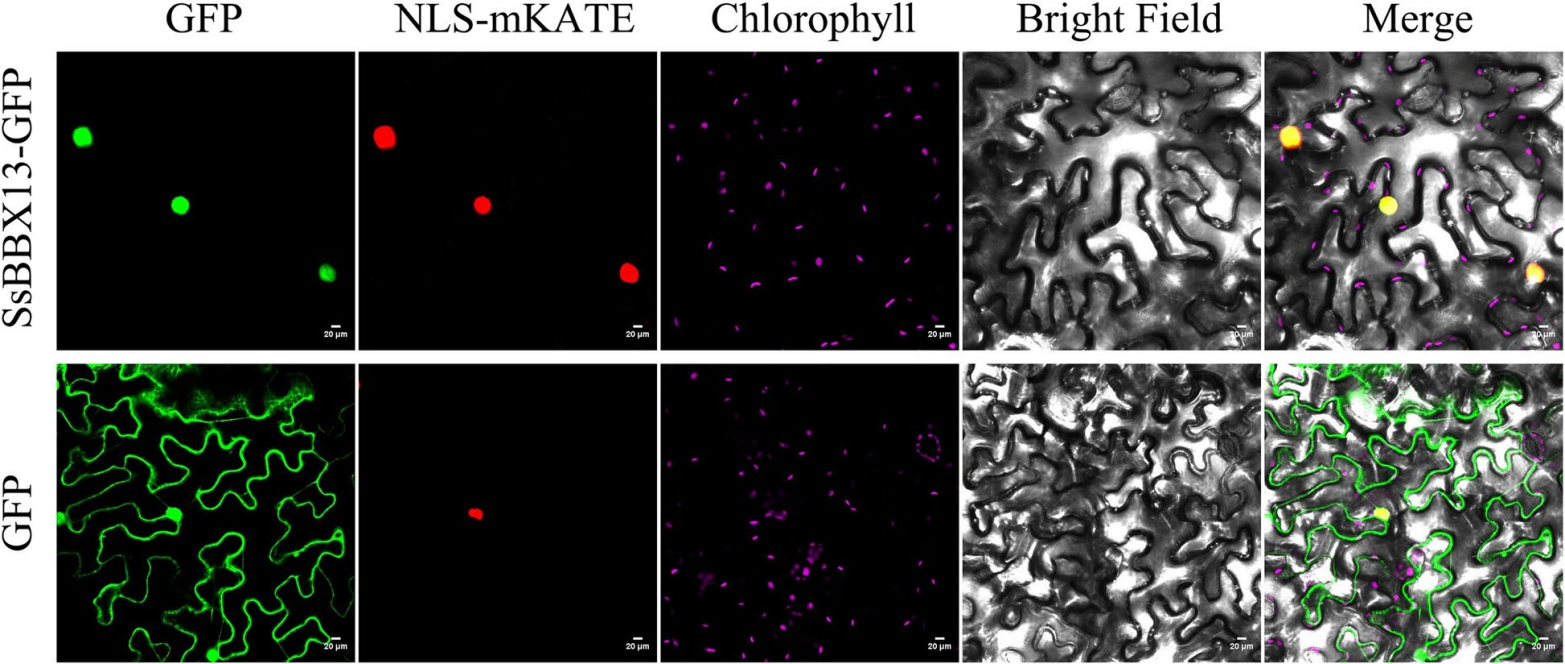
Subcellular location of GFP-fused SsBBX13 protein in *Nicotiana benthamiana* leaf epidermal cells. The SsBBX13-GFP or GFP was transiently co-expressed with the nuclear localization marker NLS-mKATE by *Agrobacterium*. Images of epidermal cells were captured using green fluorescence, mKATE fluorescence, chlorophyll fluorescence, visible light, and merged light. Confocal laser microscopy scanning was carried out 48 h after dark culture with a Zeiss LSM 800. Scale bars was 20 μm

To verify the subcellular localization of BBX, *SsBBX13*, which displays high plant growth and developmental expression patterns or is highly responsive to various stresses was selected. The ORFs of SsBBX13 together with green fluorescent protein GFP were cloned and transiently expressed in tobacco leaf epidermal cells. GFP alone was used as a control. Confocal scanning results indicated that the SsBBX13-GFP fusion protein was present in the nucleus, while GFP was distributed throughout the whole cell (Fig. 1). These results were in accordance with sequence predictions by the online tool Plant-mPLoc, which indicated that SsBBX13 was mainly located in the nucleus.

### Phylogenetic and gene structure analysis

**Fig. 2:**
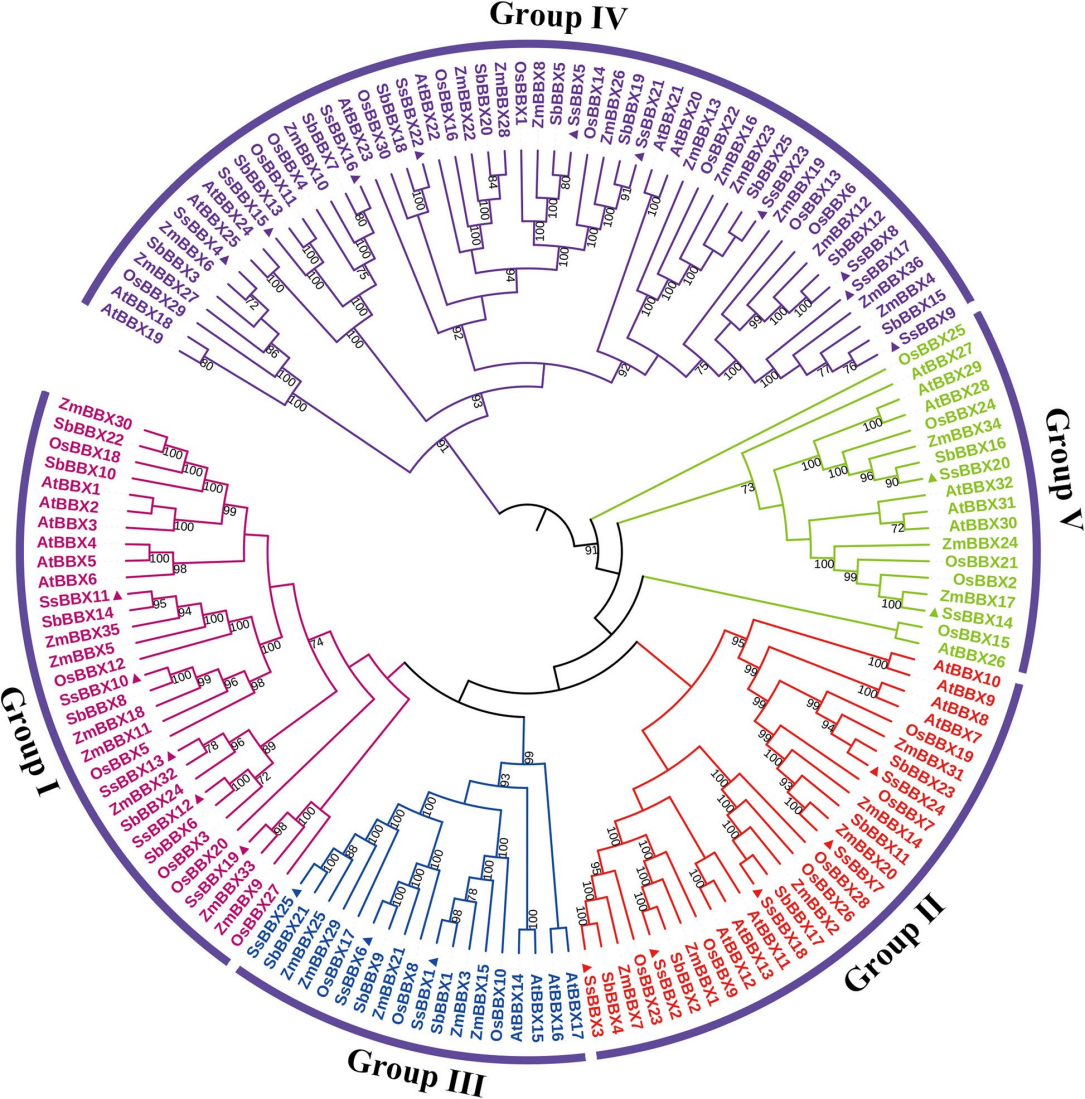
Phylogenetic tree of BBX peptide sequences of *S. spontaneum*, *A. thaliana*, *O. sativa*, *S. bicolor*, and *Z. mays*. Sequences were aligned using ClustalW software and the subsequent phylogenetic tree was constructed applying the Neighbor-joining algorithm by MEGA X software, with 1000 bootstrap replicates. Bootstrap values below 70% are not shown. Roman numerals (I–V) represent different gene clusters. The genes from each group are differentiated by color. Black solid triangles are the new BBXs found in *Saccharum*

**Fig. 3:**
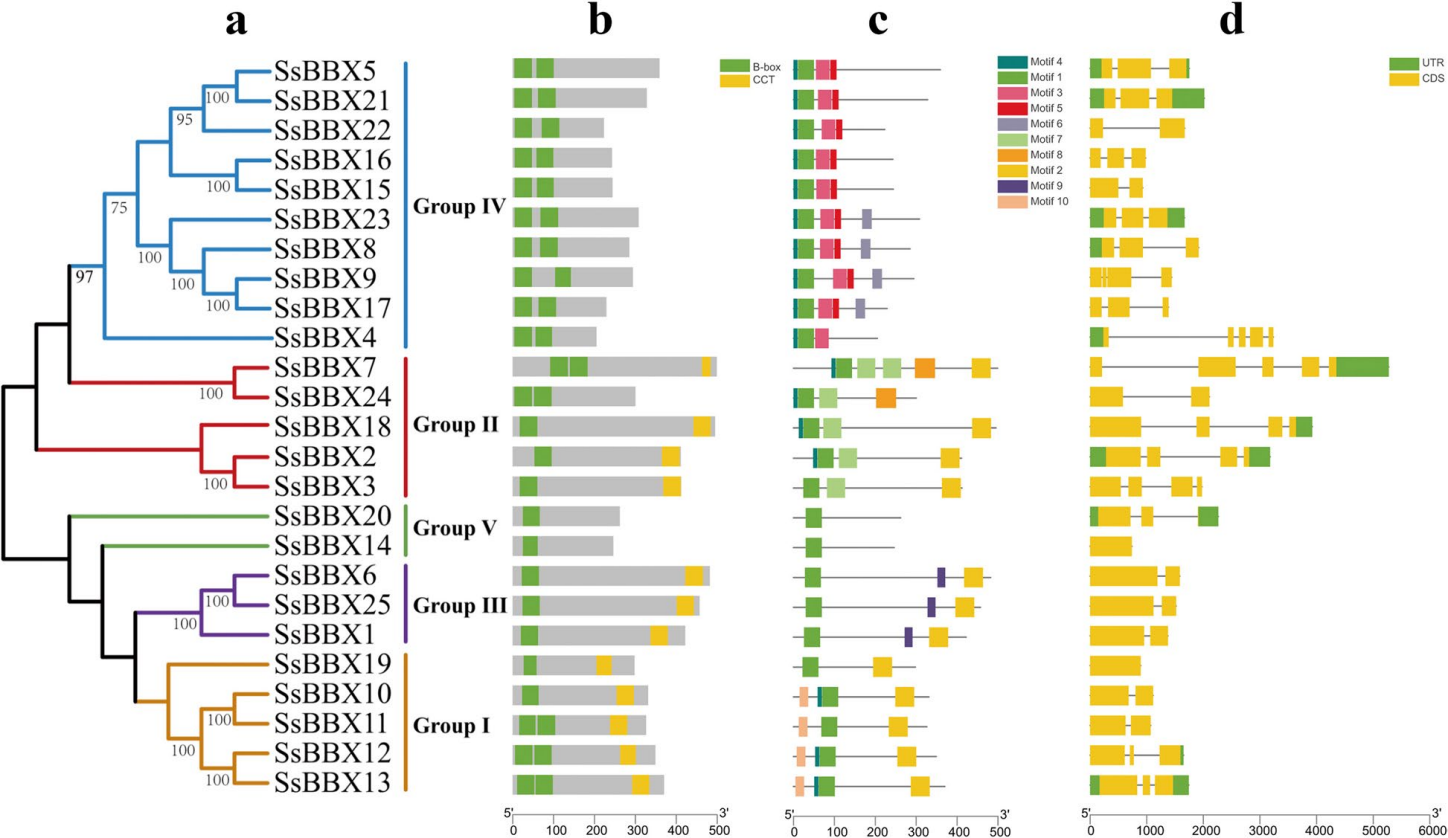
Phylogenetic tree (**a**), conserved domain (**b**), conserved motifs (**c**), and exon/intron organization (**d**) of *BBX* gene family from *S. spontaneum*. Protein sequences were aligned by ClustalW and the tree was constructed by MEGA X software using the neighbor-joining method, with 1000 bootstrap replicates. Bootstrap values below 70% are not shown. Black solid triangles are the new BBXs found in *Saccharum*. The B-box domains and CCT domain are highlighted by green and yellow boxes, respectively. Motifs of each of the SsBBXs, and 10 different motifs, are each denoted by different colored boxes. Exons and introns are represented by yellow boxes and black lines, and untranslated (UTR) 5′ - and 3′ -regions are indicated by green boxes, respectively

**Fig. 4:**
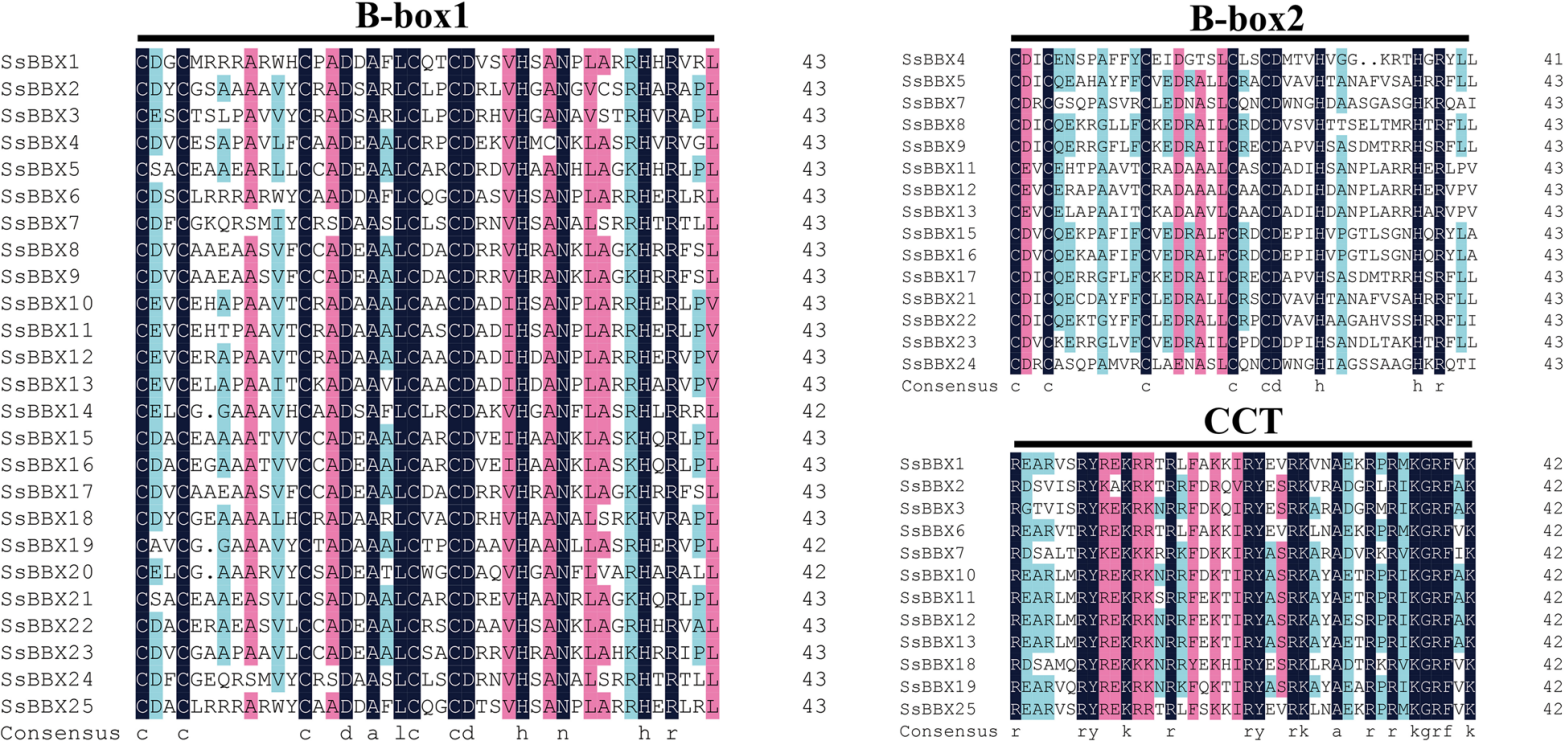
Domain composition of SsBBX proteins. Multiple sequence alignments of the domains of the SsBBXs. Multiple sequence alignments of the B-box 1, B-box 2, and CCT domains are shown. The identical and similar conserved amino acids were represented by black and pink shaded, respectively

The evolutionary relationships of BBX proteins among *S. spontaneum*, *A. thaliana*, *O. sativa*, *S. bicolor*, and *Z. mays* were determined using neighbor-joining (NJ) phylogenetic analysis. Based on the phylogenetic tree topology and a previous report in *Arabidopsis* [3, 4], all 148 BBX proteins were categorized into five groups (designated Groups I-V), and representatives of each species were found in every cluster (Fig. 2). The 25 *SsBBX* genes have a closer relationship with those of sorghum than with those of the other three species, which corresponds to a higher protein similarity between sugarcane and sorghum (Table 1 and Table S1). In all five species, Group IV had the most *BBXs* (52). Comparatively, Groups I and II each comprised 31 and 29 *BBXs*, whereas Groups III and V each included 18 *BBXs* (Fig. 2). Similarly, in *S. spontaneum*, Group IV had the most *SsBBXs* (10), and Groups I and II each comprised five *SsBBXs*, while Groups III and V each included three and two *SsBBXs* (Fig. 3a). The distribution of conserved domains in SsBBX proteins is shown in Fig. 3b. Groups I and II *SsBBXs* contained two B-box domains and one CCT domain, Group III *SsBBXs* included a single B-box domain and a CCT domain, Group IV *SsBBXs* had two B-box domains but no CCT domain, and Group V *SsBBXs* only had a single B-box domain. Some of the *SsBBXs* were not classified as expected. For instance, *SsBBX10* and *SsBBX19* were in Group I, despite having only one B-box domain and the CCT domain, indicating that they should be in Group III; likewise, *SsBBX2*, *SsBBX3*, and *SsBBX18* were in Group II, but they should also be in Group III. Protein sequence alignment and WebLogos were generated for the SsBBXs depicted in Fig. 4 and Fig. S1. The results confirmed the complete structure of the domains and suggested that the two B-box domains and the CCT domain were highly conserved in *S. spontaneum*.

To functionally characterize the *SsBBX* genes, a total of 10 motifs were determined, of which motif 1 was most conserved (Fig. 3c). Almost all genes in Group I encoded motifs 1, 2, 4, and 10 (except for *SsBBX11*). The majority of the genes in Group II contained motifs 1, 2, 4, and 7. The majority of the genes in Group III contained motifs 1, 2, and 9. The majority of the genes in Group IV contained motifs 1, 3, 4, and 5. All genes in Group V contained motif 1.

To better understand the evolution of the *SsBBX* gene family, the exons and introns were analyzed (Fig. 3d). The number of exons in the *SsBBX* genes ranged from 2 to 5. Almost all genes in Groups I, III, and IV had 2 or 3 exons, whereas Group II contained 4 or 5 exons. The last two genes in Group V comprised 1 or 2 exons. These findings demonstrate the structural similarities between the *S. spontaneum BBX* genes and the gain and loss of exons throughout evolutionary history. Moreover, the tertiary structures of the same subgroup of SsBBX proteins are highly similar (Fig. S2), indicating that the protein structure is related to species evolution.

### Synteny analysis and gene duplication prediction

**Fig. 5:**
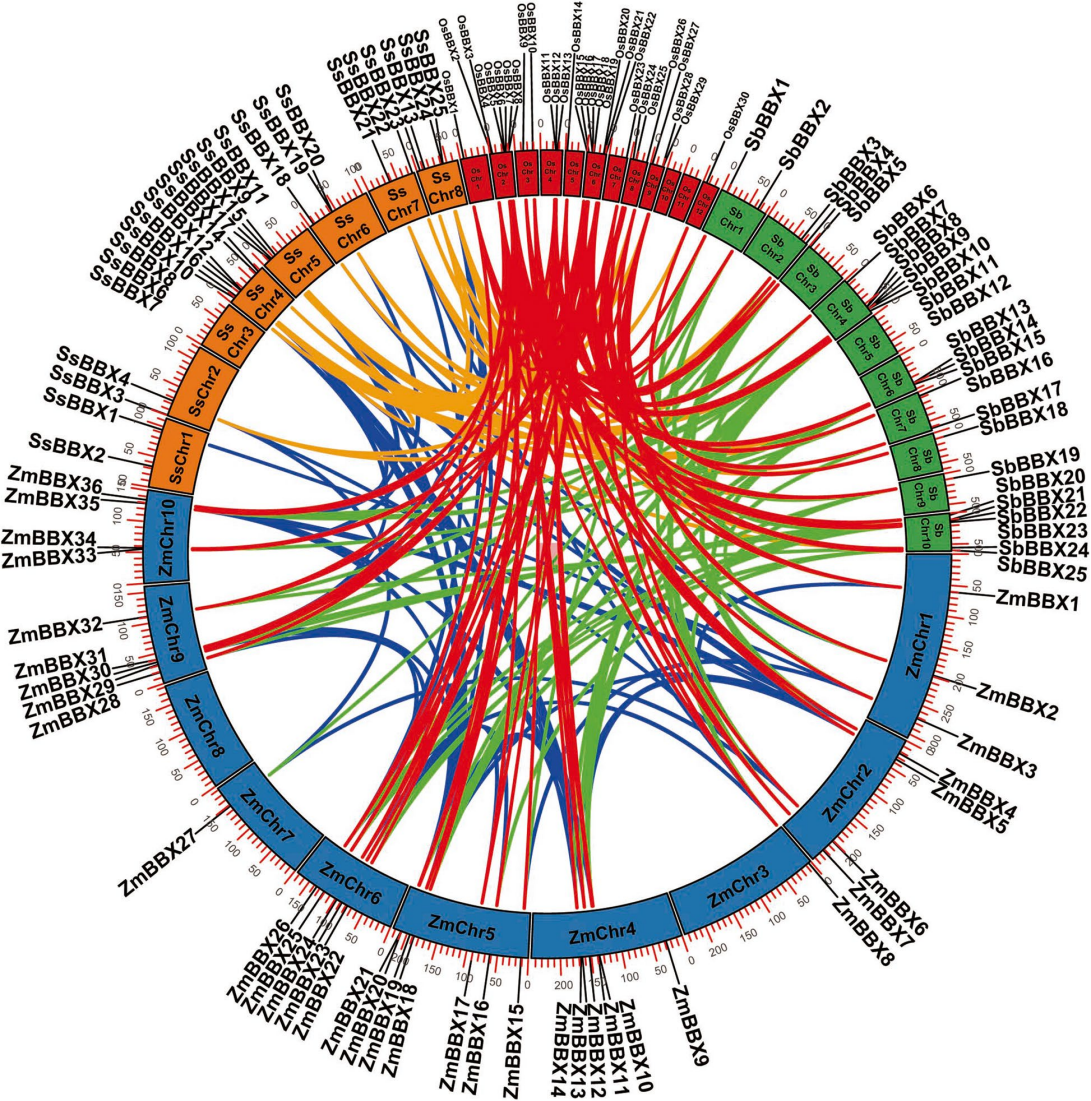
Collinearity relationships of *BBX* genes from *S. spontaneum*, *O. sativa*, *S. bicolor*, and *Z. mays*. *BBX* collinear gene pairs were mapped to their respective locus in their genome in a circular diagram. The chromosomes of *S. spontaneum*, *O. sativa*, *S. bicolor*, and *Z. mays* are indicated by boxes of different colors with the prefixes ‘Ss’, ‘Os’, ‘Sb’, and ‘Zm’, respectively. The numbers along each chromosome box represent the sequence length of the corresponding chromosome in mega-bases. Lines of different colors represent the duplication pairs of *BBX* genes

The synteny among *BBX* orthologous pairs of *S. spontaneum, O. sativa*, *S. bicolor*, and *Z. mays* was conducted by comparative analysis to determine the evolution of the *BBXs.* There were 260 pairs of orthologous genes that had syntenic relationships among these four species, including 22 pairs between *S. spontaneum* and *S. bicolor*, 26 pairs between *S. spontaneum* and *Z. mays*, 23 pairs between *S. spontaneum* and *O. sativa*, 51 pairs between *S. bicolor* and *Z. mays*, 41 pairs between *S. bicolor* and *O. sativa*, 54 pairs between *Z. mays* and *O. sativa*, and 2, 9, 9, and 23 intragenomic pairs among the four species, respectively (Table S3 and Fig. 5). Two species-specific syntenic relationships in *S. spontaneum* were observed (Table S3), namely, SsBBX8/SsBBX9 and SsBBX12/SsBBX13. Four *SsBBXs* (*SsBBX3*, *SsBBX5*, *SsBBX6*, and *SsBBX17*) were not mapped on any other *BBXs*, indicating that *S. spontaneum* has the fewest orthologous gene pairs of the four species, which implies that the *BBXs* are less conserved in *S. spontaneum* under evolutionary dynamics. In general, orthologous genes belong to the same group based on their synteny. For instance, *SsBBX4* (Group IV) was syntenic with *OsBBX29*, *SbBBX3*, and *ZmBBX6* (Group IV) (Fig. 2 and Table S3).

For *S. spontaneum*, the *BBX* genes were unequally distributed on the chromosomes (chrs) (Table 1 and Fig. 5). In *S. spontaneum*, chr4 had the most *SsBBX* genes (7), followed by chr5 (4), chr8 (4), and chr6 (3), and the remaining chrs contained 2 except chr3, which contained only one. According to the chr distribution analysis, the *BBX* genes were found mainly on chr4 in all examined *S. spontaneum*, possibly due to gene duplication events.

For the purpose of exploring the relationship between duplications and *BBX* genes, the gene duplication events of the four species above were analyzed and classified into four types (Table S4). Among the *SsBBXs*, 16 out of 25 genes (64.0%) were identified as WGD or segmental duplication genes, while six out of 25 genes (24.0%) were classified as dispersed duplicates. Similarly, the other three *BBXs* were mostly labeled as WGD or segmental duplication genes (67 out of 91, 73.6%), and the remaining *BBXs* were considered dispersed duplicates (23 out of 91, 25.3%). These results indicated that the expansion of the *BBX* gene family was primarily driven by WGD or segmental duplication.

Molecular evolution and functional divergence may occur from gene duplication events. To explore the selection pressure related to the duplication of *BBX* gene pairs within species, the ratio of the nonsynonymous substitution rate to the synonymous substitution rate (Ka/Ks) was determined. The orthologous *BBX* family members between *S. spontaneum* and *S. bicolor* were identified. The results showed that the Ka/Ks ratios of each *BBX* gene pair in *S. spontaneum* and *S. bicolor* were below one (Table 2), suggesting that the evolution of these gene pairs might have been driven mainly by purifying selection. According to the Ks value, the divergence times of *SsBBXs* and *SbBBXs* were calculated (Table 2). In terms of divergence time, *S. spontaneum* diverged from *S. bicolor* 7.779 million years ago (Mya) [51]. In the present study, *SsBBX1*, *SsBBX4*, *SsBBX7*, *SsBBX8*, *SsBBX10*, *SsBBX11*, *SsBBX12*, *SsBBX15*, *SsBBX21*, and *SsBBX23* diverged with their orthologous *SbBBXs* ranging from 5.056 Mya to 7.689 Mya, respectively, indicating that they separated after *S. spontaneum* and *S. bicolor* (7.779 Mya). In contrast, the remaining 12 *SsBBXs* diverged from their orthologs 7.967 Mya and 17.092 Mya, respectively, indicating that they separated before *S. spontaneum* and *S. bicolor*.

**Table 2:** Nonsynonymous (Ka) and synonymous (Ks) substitution rates and estimated divergence time for paralogous BBX genes in *Saccharum* and sorghum

Paralogous pairs	Ka	Ks	Ka/Ks	Divergence time (Mya)
SsBBX1 vs. SbBBX1	0.026	0.067	0.380	5.522
SsBBX2 vs. SbBBX2	0.020	0.097	0.202	7.967
SsBBX3 vs. SbBBX4	0.063	0.112	0.561	9.206
SsBBX4 vs. SbBBX3	0.017	0.094	0.180	7.689
SsBBX5 vs. SbBBX5	0.029	0.154	0.187	12.655
SsBBX6 vs. SbBBX9	0.023	0.114	0.199	9.351
SsBBX7 vs. SbBBX11	0.015	0.087	0.175	7.109
SsBBX8 vs. SbBBX12	0.029	0.079	0.374	6.456
SsBBX10 vs. SbBBX8	0.007	0.062	0.110	5.056
SsBBX11 vs. SbBBX14	0.011	0.062	0.183	5.061
SsBBX12 vs. SbBBX6	0.035	0.080	0.436	6.535
SsBBX13 vs. SbBBX24	0.028	0.122	0.231	9.968
SsBBX15 vs. SbBBX13	0.025	0.067	0.376	5.452
SsBBX16 vs. SbBBX7	0.009	0.137	0.067	11.197
SsBBX17 vs. SbBBX15	0.034	0.110	0.313	9.013
SsBBX18 vs. SbBBX17	0.035	0.121	0.291	9.912
SsBBX20 vs. SbBBX16	0.038	0.118	0.322	9.703
SsBBX21 vs. SbBBX19	0.016	0.079	0.202	6.479
SsBBX22 vs. SbBBX18	0.063	0.115	0.551	9.408
SsBBX23 vs. SbBBX25	0.059	0.086	0.690	7.047
SsBBX24 vs. SbBBX23	0.069	0.209	0.332	17.092
SsBBX25 vs. SbBBX21	0.028	0.106	0.266	8.657

### Cis-acting elements of the *SsBBX* gene family

**Fig. 6:**
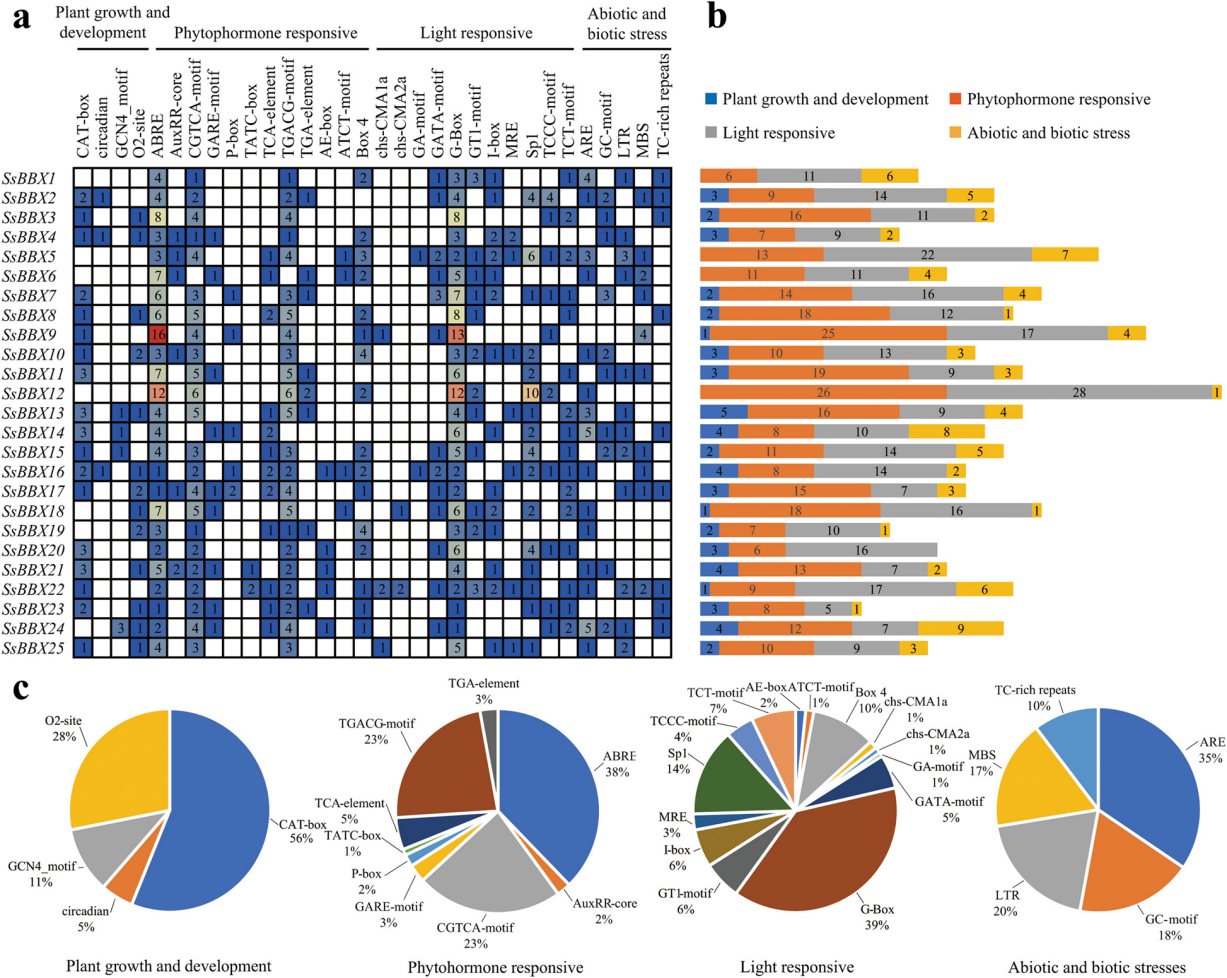
Identification of cis-acting elements in all *SsBBXs*. **a** Four categories of cis-acting elements in the *SsBBXs*. Different colors and numbers of the heatmap box represented the number of different elements in these *SsBBXs*. Red indicates higher elements while blue indicates lower elements. **b** Histogram of the cis-acting elements in each *SsBBX* gene. **c** Pie charts of different sizes indicated the ratio of each promoter element in each category, respectively

The cis-acting element sequences in the promoter regions were used to better understand the transcriptional regulation and gene function of the *SsBBXs*. In total, 773 cis-acting elements were identified and divided into four categories, including plant growth and development, phytohormone responsive, light responsive, and abiotic/biotic stress (Fig. 6). Among the plant growth and development elements (Fig. 6c), 56% were associated with meristem expression (CAT-box), followed by zein metabolism regulation (O2-site), endosperm expression regulation (GCN4_motif), and circadian rhythm. The phytohormone responsive and light responsive elements accounted for the largest proportion (both up to 41%) (Fig. 6a and b). The light responsive elements contain diverse kinds of cis-acting elements, including the G-box, Sp1, Box 4, TCT-motif, I-box, GT1-motif, GATA-motif, TCCC-motif, and MRE (Fig. 6c). Among the phytohormone responsive elements (Fig. 6c), ABA-related elements (ABRE) were the most common cis-acting elements (38%). The other elements were the MeJA responsive (CGTCA-motif and TGACG-motif), the salicylic acid responsive (TCA-element), the gibberellin responsive (GARE-motif, P-box, and TATC-box), and the auxin responsive (TGA-element and AuxRR-core), suggesting that *SsBBXs* might be regulated by different hormones. Among the abiotic and biotic stress elements (Fig. 6c), anaerobic induction (ARE) was the most common cis-acting element (35%), followed by the low temperature response element (LTR), GC-motif element, drought stress response element (MBS), and defense stress related element (TC-rich repeats), implying that *SsBBXs* contribute to various stress responses.

### Expression patterns of *BBX* genes during various plant developmental stages and in different tissues

**Fig. 7:**
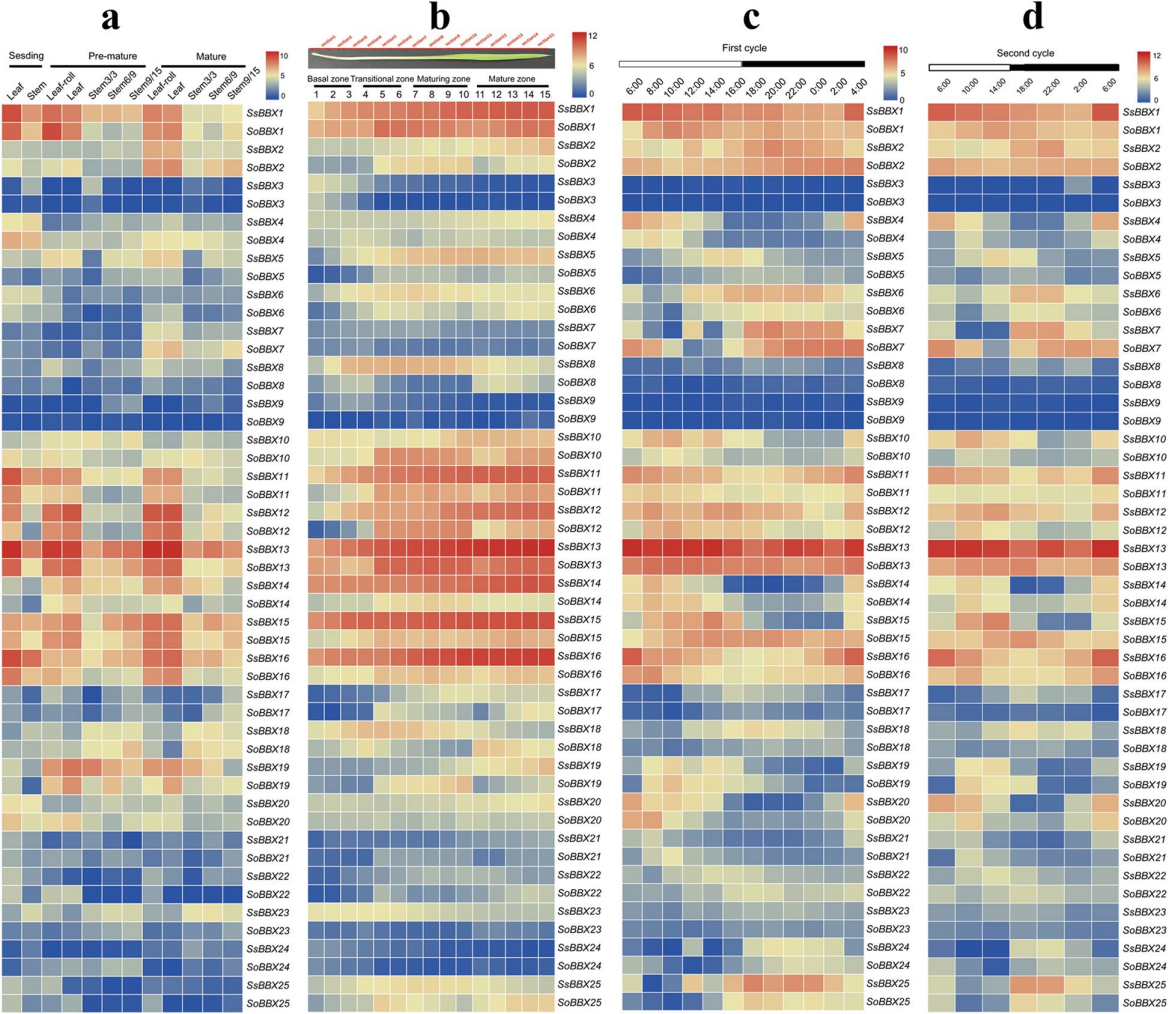
The expression pattern of *BBX* genes based on log_2_-transformed FPKM values in three treatments. **a** Heatmap based on gene expression in different tissues at different stages in *S. spontaneum* and *S. officinarum.***b** Heatmap based on gene expression across leaf gradients in *S. spontaneum* and *S. officinarum*. c&d) Heatmap based on gene expression during the diurnal cycles in *S. spontaneum* and *S. officinarum*. The heat map was plotted with the TBtools software (v1.098). Expression values were normalized to genes based on the average linkage algorithm. The scale bar represents the log2 normalized expression. Red indicates higher expression while blue indicates lower expression

To explore the expression patterns of the *BBX* genes in diverse plant growth and development processes, we investigated the expression patterns of the sugarcane *BBX* genes during development. The expression patterns of the *BBXs* in 2 *Saccharum* species, *S. spontaneum* and *S. officinarum*, were analyzed and compared using available transcriptome data [43] during 3 developmental stages in various tissues (Fig. 7a). Gene expression levels varied among genes, and some genes were expressed differently depending on the tissue. Among the 25 *BBX* genes analyzed, 2 genes (*BBX1* and *BBX13*) were relatively highly expressed throughout all developmental stages and tissues in sugarcane, suggesting their overall involvement in plant development, whereas 7 genes (*BBX3/9/17/21/22/23/24*) demonstrated extremely low or undetectable levels in all examined tissues in different growth stages. Additionally, almost all *BBXs* (except *BBX10* and *BBX18*) were more highly expressed in leaves than in stems. Notably, 9 genes (*BBX5/8/11/12/13/14/15/16/19*) were expressed at higher levels in *S. spontaneum* than in *S. officinarum*, while 5 genes (*BBX2/4/7/10/20*) showed the opposite trend, and 4 genes (*BBX1/6/18/25*) were expressed at equal levels in 2 *Saccharum* species. The results presented here suggested that *BBX* genes function differently at various developmental stages and might affect biological processes in diverse tissues. To confirm this finding, detailed analyses of their expression levels in roots and in meristematic and reproductive tissues are needed for a more complete understanding of their functions.

The study of the expression pattern of *BBX* genes in continuously developing leaf segment gradients from *S. spontaneum* and *S. officinarum* provided further insights into the functional divergence of *BBX* genes for photosynthesis and sugar transport in source tissues (Fig. 7b). Similar to the expression pattern at different developmental stages, 2 genes (*BBX1* and *BBX13*) were relatively highly expressed in all examined leaf segments, indicating their overall involvement in sugarcane photosynthesis and sugar transport, whereas the expression of 8 genes (*BBX3/7/9/17/21/22/23/24*) was low or undetectable, suggesting their limited contribution to photosynthesis and sugar transport. Interestingly, the expression of 8 genes (*BBX1/10/11/12/13/14/15/16*) increased from the basal zone to the mature zone in *S. spontaneum*, while the expression of these genes increased in the transition zone and mature zone but extremely low in the basal zone in *S. officinarum*. Notably, 11 genes (*BBX4/5/6/8/11/12/13/14/15/16/23*) were expressed at higher levels in *S. spontaneum* than in *S. officinarum*, while only 3 genes (*BBX1/18/25*) were expressed at equal levels in the 2 *Saccharum* species. These results indicated functional divergence of the *BBX* genes in leaf segment gradients, and interspecies differentiation could also contribute to this divergence.

To examine the expression patterns of the *BBXs* during diurnal cycles, we examined the expression patterns of mature leaves in the 2 *Saccharum* species over a 24 h period at 2 h intervals followed by 4 h intervals over another 24 h (Fig. 7c and d). Similar to the RNA-seq profiles at different developmental stages as well as in the leaf segment gradient, 2 genes (*BBX1* and *BBX13*) were expressed at very low or undetectable levels in both *Saccharum* species, whereas 8 genes (*BBX3/8/9/17/21/22/23/24*) were expressed at either very low or undetectable levels in all examined leaf segments, further supporting their involvement or limited roles in growth and development. Additionally, 6 genes (*BBX4/10/14/16/19/20*) were expressed higher in the daytime than at night and displayed the lowest expression level at night in both *Saccharum* species, whereas some genes (*BBX2/6/7/15/18/25*) were expressed at the highest levels in the evening and constitutively expressed at other times in either *S. spontaneum* or *S. officinarum*. Notably, 10 genes (*BBX4/5/6/10/11/12/13/16/18/25*) were expressed at higher levels in *S. spontaneum* than in *S. officinarum*. These findings imply functional differences of the *BBX* genes in diurnal rhythms.

### Expression patterns of *BBX* genes under low-nitrogen stress

**Fig. 8:**
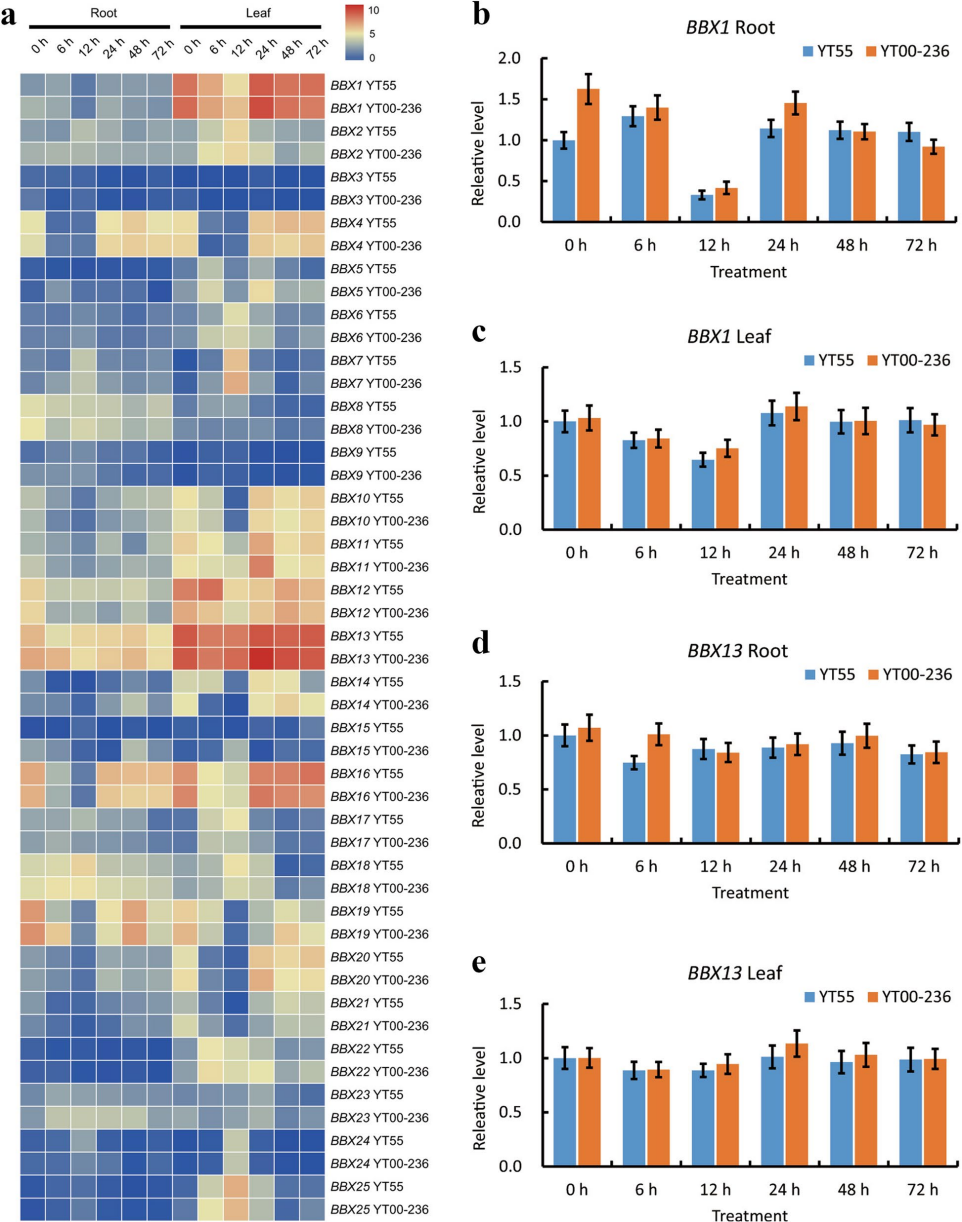
The expression pattern of *BBX* genes in *Saccharum* hybrid YT55 and YT00–236 under low-nitrogen stress conditions based on log_2_-transformed FPKM values (**a**) and verification of *BBX1* and *BBX13* expressions in root and leaf under low-nitrogen stress by RT-qPCR (**b**, **c**, **d**, and **e**). YT55 and YT00–236 seedlings were subjected to 100 mM nitrogen treatment, and samples were collected at 0, 6, 12, 24, 48, and 72 h after the treatment. The expression at 0 h was set to 1.0. Values are mean ± SD of three replicates

To determine the functional differentiation of the sugarcane *BBX* genes in response to low-nitrogen (LN) stress, we evaluated the expression profiles of the *BBXs* under LN stress (Fig. 8a). The expression patterns were observed for the *BBX* genes in roots and leaves of 2 *Saccharum* hybrid varieties YT55 and YT00–236 at 0 h, 6 h, 12 h, 24 h, 48 h, and 72 h. A different expression pattern was exhibited under LN treatment in the *BBX* family. Among the 25 *BBX* genes analyzed in both *Saccharum* hybrid varieties, 2 genes (*BBX1* and *BBX13*) were significantly highly expressed in the leaves, while 10 genes (*BBX3/5/6/9/15/17/21/22/23/2*4) showed noticeably low or undiscovered expression levels under all levels of LN stress, implying their involvement or restricted functions in abiotic stress. Obviously, 4 genes ((*BBX1/4/13/16*) were down-regulated at 12 h and 48 h but up-regulated at 24 h and maintained a constant high level at 72 h in the leaves of both *Saccharum* hybrid varieties. Importantly, 12 genes (*BBX1/2/4/7/10/11/12/13/14/16/20/25*) showed higher expression levels in leaves than in stems, while 3 genes (*BBX8/18/19*) showed the opposite trend. Intriguingly, in both roots and leaves, *BBX1* and *BBX13* expressions were higher in YT00–236 than in YT55. These results may elucidate the differential tolerance to LN between YT55 and YT00–236. The transcriptional expression levels of *BBX1* and *BBX13* at 0 h, 6 h, 12 h, 24 h, 48 h, and 72 h under LN stress in YT55 and YT00–236 were validated by RT-qPCR (Fig. 8b, c, d, and e). The FPKM values and relative expression levels were favorably correlated, supporting the validity of the transcriptome-based estimates of gene expression.

## Discussion

The *BBX* gene family is extensively distributed in plants as a kind of transcription factor that participates in diverse developmental processes, including light signal transduction, flowering, and stress signaling pathways [4]. The function and evolution of the *BBX* genes in different species have been evaluated with an unbiased bioinformatics method including *Arabidopsis* [3], rice (*Oryza sativa*) [6], pear (*Pyrus pyrifolia*) [7], and tomato (*Solanum lycopersicum*) [9]. Previous research has revealed wide variation in the number of *BBXs* among plant species [6, 52]. Sugarcane (*Saccharum* spp.) is a vital crop worldwide and offers essential sugar and energy for daily life [53]. Therefore, the investigation of sugarcane *BBX* genes will help elucidate and improve sugarcane development. However, knowledge about the sugarcane *BBX* family is scarce. In this study, 25 *SsBBXs* were found in the wild sugarcane *S. spontaneum* genome database. Characterization of the phylogenetic relationship, gene structure, synteny, gene duplication, and expression profiling of the *BBX* gene family in sugarcane was carried out to explore their evolution and possible functional divergence.

The localization of proteins in diverse organelles may correlate with their function [54, 55]. Previous reports have suggested that the nuclear localization signal (NLS) plays a crucial role in localizing the BBX protein to the nucleus and is a part of the CCT domain [4], which was supported by our results on the subcellular location of BBXs in sugarcane, sorghum, maize, and rice (Table S4). SsBBX protein sequences were Blast search, and the results indicated that 5.2% ~ 70.0% sequence similarities within the members and 80.0% ~ 97.0% shared homology with *S. bicolor* (Table S1). The findings of this study were in agreement with those of earlier studies on *A. thaliana* [3] and other higher plants [6], suggesting high differentiation among the members of the *BBX* gene family and great conservation among the same type of *BBXs*.

Gene function of the *BBX* family in various species can be predicted based on evolutionary history analyses [5]. According to the analysis of the phylogenetic relationships, the sugarcane *BBX* family members were divided into five groups, with Group IV containing the most *SsBBX* genes, which was consistent with the *BBXs* in *A. thaliana*, pear, potato, and tomato [3, 7, 9, 56, 57]. The results implied that *BBXs* clustering in the same group shared common gene structures, whereas slight differences played a vital role in gene evolution. The *SsBBX* family members from Groups I, II, and IV harbored two B-box domains, with the exception of *SsBBX10*/*19* and *SsBBX2/3/18*. These five *SsBBXs* should have been in Group III, yet they were in Group I and II, respectively (Fig. 3). Similar to previous reports, during the process of evolution, the B-box 2 domain of some BBX proteins was lost, indicating that a deletion event might have occurred in the B-box 2 domain [52, 58]. WebLogos analysis verified that the CCT domain was highly conserved and that the two B-box domains were highly homologous in the various *SsBBX* genes, suggesting that the *BBX* genes of diverse plant species might have a similar ancestor, which was consistent with previous studies [59, 60]. Furthermore, the rapid expansion of *BBX* gene families during evolution, and the fact that BBX proteins are highly conserved throughout the plant kingdom indicate that these proteins might play vital roles in the adaptation of terrestrial plants [4, 61, 62].

Increasingly, research on higher plant genome sequencing has revealed that the generation of novel genes is related to gene duplication. Repeated episodes of tandem duplication and segmental duplication (or whole-genome duplication, WGD) are two main classes of gene duplication events across the plant genome [63]. Segmental duplication/WGD is a large-scale duplication event that leads to amplification of a gene family [64]. The *Saccharum* genome has undergone two WGD events, which were directly involved in most of the expansion of numerous gene families [26]. In *S. spontaneum*, almost all *SsBBXs* were associated with segmental/WGD, and none of the *SsBBX* genes were defined to be tandem duplications (Table S4). These results implied that WGD or segment duplications were the major driving force for the expansion of *SsBBX* gene family members, which was consistent with the previously described expansion of *BBX* family members in *Poaceae* [25] and other plant species [5]. Moreover, previous research reported that tandem duplication often occurred in the large and rapidly evolving gene family, whereas segmental duplication normally occurred in the slowly evolving gene family [63]. The present results suggested that *the SsBBX* gene family should be categorized as a slow-evolving gene family. The ancestor of *S. officinarum*, *S. spontaneum*, diverged approximately 7.779 Mya from sorghum, whereas *S. spontaneum* diverged approximately 769 thousand years ago from *S. officinarum* [51]. In this study, the divergence times of ten out of 22 *SsBBXs* with their ortholos *Sorghum bicolor BBXs* (*SbBBXs*) were shorter than 7.779 Mya (Table 2), whereas the remaining 12 of the 22 *SsBBXs* diverged longer ago than 7.779 Mya. Based on these findings, the duplications of the *SsBBXs* with their orthologs most likely occurred near the divergence of *S. spontaneum* and sorghum. The Ka/Ks ratios among paralogous pairs of *SsBBXs* and their orthologous *SbBBXs* were calculated (Table 2). Generally, Ka/Ks ratios greater than and less than 1 indicate positive and purifying selection pressures, respectively. Ka/Ks ratios equal to 1 indicate neutral selection. All Ka/Ks ratios of *BBXs* in sugarcane were less than 1, suggesting that the evolution of *BBX* members was influenced by strong purifying selection. Purifying selection pressure may be a contributor to the conserved structures of *BBX* family members during evolution. Generally, these results suggest that the large-scale duplication event (WGD or segmental duplication) may help sustain the conserved structures of the *SsBBX* gene family during evolution under purifying selection pressure.

There is evidence that *BBX* genes are involved in a variety of plant growth and development processes, such as shade avoidance, seedling photomorphogenesis, chlorophyll accumulation, and flowering [4, 11]. There is increasing evidence that *BBXs* exhibit specialized gene expression patterns associated with their functions. The expression profiles revealed that more than half of the *SsBBXs* showed spatial variations under multiple plant growth and development processes in sugarcane, and similar findings were found in other plants [5]. Almost all *BBXs* in sugarcane were highly expressed in leaves, suggesting that they might perform functions in controlling leaf growth, which is consistent with the functional activities of the *BBX* genes that have previously been discovered [25, 65]. Expression analysis was carried out with *BBX1* and *BBX13*, which were mainly expressed at different stages of vegetative growth, suggesting the conservation and importance of gene function. We observed that *BBX1/10/11/12/13/14/15/16* may have similar functions to *A. thaliana AtBBX* genes and were most highly expressed in the mature zone of leaves; these genes are primarily involved in processes associated with light and controlled by circadian rhythm [4]. Taken together, our results imply that sugarcane *BBX* family members contribute to leaf development as well as photosynthesis and sugar transport.

Environmental conditions affect the growth and development of plants as well as their productivity [66]. In the current study, cis-acting element analysis, transcriptome data, and RT-qPCR approaches were applied to explore the functions of sugarcane *BBXs* under various stresses and hormone treatments (Fig. 6, Fig. 8, and Fig. S3). Transcriptome data and RT-qPCR were used to deeply understand the stress and hormonal response mechanisms in sugarcane *BBXs*. Our results showed that the *BBXs* exhibited divergent expression profiles under hormonal treatment and stresses in roots, leaves, and buds. For instance, *BBX1* and *BBX13* were up-regulated by low-nitrogen, cold, drought, smut, and ABA in both leaves and buds at most of the time points, while *BBX1* was significantly down-regulated by low-nitrogen and low-potassium conditions in roots at most of the time points. Accumulating evidence has demonstrated that plant *BBX* genes are involved in response to various stresses, and can be regulated by exogenous hormones [9, 25, 60]. In pear, 43.2% of *PbBBXs* (16 out of 37) were controlled by drought stress, and 81.3% (13 out of 16) were up- or down-regulated after dehydration stress within 12 h [57]. *CmBBX19* was down-regulated under drought treatment [67]. In *Petunia*, three *PhBBX* genes were regulated by drought treatment, eight *PhBBX* genes were regulated by salt treatment, and 18 *PhBBX* genes were regulated by cold treatment [68]. In apple, *MdBBX10* was significantly affected by sodium chloride and polyethylene glycol (PEG) in leaves and roots [69]. In rice, *OsBBX9* was up-regulated under nickel stress [25]. In *Brassica napus*, *BnaBBXs* participated in regulating nutrient assimilation [70]. In this research, it was revealed that the transcriptional levels of more than half of the *SsBBXs* (15 out of 25) were affected by low-nitrogen stress, indicating that most of the genes are involved in the sugarcane stress response. Gene expression profiles can provide important information into gene function, and the RT-qPCR sinvestigation of two chosen *SsBBXs* revealed tissue-specific expression patterns in roots and leaves (Fig. 8). On YT55 and YT00–236, several studies have been done to study how nitrogen is used and regulated [71, 72]. By examining physiological and morphological variables like as nitrogen concentration, dry matter content, and root phenotype, it was possible to determine how these two varieties used nitrogen. All of the indicators between YT55 and YT00–236 showed a discernible, implying that YT55 had a greater NUE than YT00–236 [71, 72]. Nevertheless, there was no obvious justification for the variation in NUE between YT55 and YT00–236. In the present study, the expression levels of *BBX1* and *BBX13* in both leaves and roots of YT00–236 were typically higher than those of YT55. The expression levels of *BBX1* and *BBX13* under LN stress may explain the differentiation in NUE between YT55 and YT00–236. Since a limited number of researchers have examined the role of the *BBX* genes in nutritional stress, additional studies will be conducted to investigate the function of *BBX* gene family members as they relate to abiotic stress tolerance.

## Conclusions

In the current study, we characterized 25 *SsBBXs* in the wild sugarcane genome database and systematically researched their genome-wide identification and expression patterns. Phylogenetic relationships, evolutionary analysis, and gene structure analysis elucidated the conservation of *SsBBXs* and revealed that some members had diverged from their ancestors. The transcription of certain sugarcane *BBXs* can be induced or repressed under various stresses and hormonal treatments (low-nitrogen, low-potassium, cold, drought, smut, and ABA), indicating that they may have essential functions in various biological processes. In summary, the data produced in this work may lay the foundation for further functional characterizations of sugarcane *BBX* genes, particularly concerning abiotic stress responses and plant development, thereby promoting their application in cultivated sugarcane breeding.

## Supplementary Information

**Additional file 1** The amino acid sequences of the BBX family used in this study.

**Additional file 2: Supplementary Table 1.** Sequence matrix identities (%) of BBX proteins calculated by BioEdit software.

**Additional file 3: Supplementary Table 2.** The *BBX* gene alleles in *Saccharum spontaneum.*

**Additional file 4: Table S3.** Information about orthologous genes among *S. spontaneum*, maize, rice and sorghum.

**Additional file 5: Table S4.** Gene duplications, number of transmembrane domains, and subcellular location of the BBXs among *S. spontaneum*, maize, rice and sorghum.

**Additional file 6: Table S5.** The cloning primers for SsBBX13.

**Additional file 7: Table S6.** The primers for RT-qPCR verification of two *BBX* genes in *Saccharum* hybrid YT55 and YT00-236.

**Additional file 8: Fig. S1.** WebLogos of the amino acid sequences alignment of B-box1, B-box2, and CCT were shown. The y-axis and x-axis indicated the conservation rate of each amino acid and the conserved sequences of the domain, respectively. The height of each letter indicates how conserved the residue is across all proteins. **Fig. S2.** The tertiary structure modeling of SsBBX proteins. The structure image was generated using the SWISS-MODEL software. **Fig. S3.** Expression patterns of the sugarcane *BBX* genes from the sugarcane transcriptome data in five different conditions. (a) Expression patterns of the *BBX* genes in the roots of *Saccharum* hybrid cultivar YT55 under low-potassium stress, and samples were collected at 0, 6, 12, 24, 48, and 72 h (PRJNA262715). (b) Expression patterns of the *BBX* genes in the leaves of *Saccharum* hybrid cultivar GX87–16 under cold stress, and samples were collected at 0, 0.5, 1, and 6 h (PRJNA636260). (c) Expression patterns of the *BBX* genes in the leaves of *Saccharum* hybrid cultivar Co_06022 (susceptible cultivar) and Co_8021 (resistant cultivar) after 0, 2, 6, and 10 d drought stress and recovery treatment (PRJNA590595). (d) Expression patterns of the *BBX* genes in the buds of *Saccharum* hybrid cultivar ROC22 (susceptible cultivar) and YC05–179 (resistant cultivar) after smut pathogen infection at 0, 1, 2, and 5 d (PRJCA011580). (e) Expression patterns of the *BBX* genes in the buds of *Saccharum* hybrid cultivar ROC22 (susceptible cultivar) and GT42 (resistant cultivar) after ABA treatment at 0, 1, and 6 h (PRJNA555450). The heat map was plotted with the TBtools software (v1.098), with the transcript level of the *BBX* genes transformed as log_2_ FPKM (fragments per kilobase million), ranging from blue (low expression level) to red (high expression level).

## Data Availability

All RNA-seq data can be downloaded from the GenBank website with the BioProject ID- PRJNA262715, PRJNA636260, PRJNA590595, and PRJNA555450, and the NGDC databases with the BioProject ID- PRJCA011580. The *S. spontaneum* genome project was deposited into Genbank with accession numbers: QVOL00000000.
