# Supplementary material for: Characterization and expression profiles of the B-box gene family during plant growth and under low-nitrogen stress in Saccharum
Source: BMC Genomics. 2023 Feb 17;24:79. doi: 10.1186/s12864-023-09185-9 (PMC9936747; doi:10.1186/s12864-023-09185-9)
Supplement: Supplementary file 1 — Additional file 1 The amino acid sequences of the BBX family used in this study. [file 12864_2023_9185_MOESM1_ESM.docx]

**File S1**. The amino acid sequences of the BBX family used in this study.

>Sspon.01G0026670-1A_SsBBX1

MTSAGAAAGAALGARTARSCDGCMRRRARWHCPADDAFLCQTCDVSVHSANPLARRHHRVRLPSASCSSPPCDPDAPTWLHGLKRRPRTPRSKPGGGKHEATTPNSIALAASTAVPDLEAEESGSGIVGDNDDEHGFQDDDEDLLYRVPVFDPMLAEFYNPVADEGEQKPLAEFYNPVADDGEQKPACLMPPLVETSLEFAYGGSAEADGLSAGFDVPDMELASFAADMESLLMGVNDGFDDLGFLDEEKPQVNADADLVAMAAPATEREDKKRKRPEMILKLNYEGVIASWVRDGGSPWFHGERPHLDPYELWSDFPAGSRGLFGGAVTAVTGGEREARVSRYREKRRTRLFAKKIRYEVRKVNAEKRPRMKGRFVKRTTLPPLPRPPPQQQQQQQKQLARTLPHVGMVLAPPPVANGRFH

>Sspon.01G0039310-1B_SsBBX2

MGEGEDDQRNQMLGAGRDHEPERAEAEEAKKPAPGEAEAGGDGAGTGTEAATCDYCGSAAAAVYCRADSARLCLPCDRLVHGANGVCSRHARAPLCADCHAAGAVFRRASSSAFLCSNCDFGRHRDGGDPPLHDRCAVQPYSGCPPAGDLAALLGVALFDKPAAEDGAWWNIWEEPQVLSLEDLIVPTTPCHGFEPLLTPSSPKNRSISPDGKLNEEILRQLGELAESDGGVQASAGREEAQQAGGDQFPSWASPQYATGHGNFGTENNHEVATMPTPVYENGRWNNCDLDALNDACKVEVAYDQVPVNSAEPCLSSFAPLSEICPSMSNGNSMEDNHQANPGIGMPMQGLPKRTGFDVVPCPDRDSVISRYKAKRKTRRFDRQVRYESRKVRADGRLRIKGRFAKANQT

>Sspon.02G0000150-1A_SsBBX3

MSSAADAASGKEAPAPACESCTSLPAVVYCRADSARLCLPCDRHVHGANAVSTRHVRAPLCAGCRAAAAATAAAGGSFLCANCHFGSEKDERHPDGGDPQPLHHDRGAVEGYAGCPSIAELAAILGVAGCDEKAAAAAGDGGWWPASAWEEPQVLRLEDVIVPTTSCHGLQPLLTPPSPKNLSSGGEMADEVVRQLGELAKLEEAVAAAFAEMEPADGEQLPPWTSPEVGIGHADFGALDADAAWHDAATIAAVPSTEEQEAWIATGCDVDACRTEVEEAREHAALAPAPAPADPCLSSFVEMSEICPASVTALNHGGIGGTADVDNTGKTDAGTAPRPQTPPVLMPVPEPEPLTEKKGSYDVAYPDRGTVISRYKEKRKNRRFDKQIRYESRKARADGRMRIKGRFAKSGE

>Sspon.02G0036490-1B_SsBBX4

MRTICDVCESAPAVLFCAADEAALCRPCDEKVHMCNKLASRHVRVGLADPNKLARCDICENSPAFFYCEIDGTSLCLSCDMTVHVGGKRTHGRYLLLRQRVEFPGDKPGHMDDVPMEIKDPENQRDQKTVPKEQMANHHNGDDPASDGNCDDQGNIDSKMIDLNMRPVRTHGQGSNSQTQGVDLSVNHHDSPGVVPTSNSERDAN

>Sspon.03G0036880-1B_SsBBX5

MKVLCSACEAAEARLLCCADEAALCARCDRDVHAANHLAGKHHRLPLLPPADVSAPNCDICQEAHAYFFCVEDRALLCRACDVAVHTANAFVSAHRRFLLTGVQVGLQPDAAAAAQDPDPHPPTAAAAADPLQTPPPPDRKASAGGGSPAPLYSDDDIDWAAGADAAGVGVSVAVGLPDWALVHEQFGAPPVPRSAEPALARTPASKRSPRRSLAAAFTVQGGGGGLAAGLPDWPLDEFFGFSEYSAGLGFAENGTSKADSGKLGSTDGSPAGRSSSDASQDFFGEVPEFHQWSVPELPSPPTASGLHWQGGPRHGATTNTDTDTAAVFVPDISSTENPFRCYATAAGQPPAKRRRRC

>Sspon.04G0004240-2C_SsBBX6

MSSSKHAAAGAAAVGGKAARACDSCLRRRARWYCAADDAFLCQGCDASVHSANPLARRHERLRLRPTTSRPDPPHSTLEAGVGVAASTSTWKKRQQQQVAPAAWSKRKARTRRPHVKSVGQLLSRKLVVVPEVASVESSEERKAEEEEEEEEEEEQQLLYCVPTFDRALAELCTPPPPPVDDPTATASSSCCRDDVDVAVENTKAPASPAVVVAESPVQQLPDSFAGFGPTDAELREFAADMEALLGQGLGDSNELDESFYMESLGLMTTQQAEDVGRVKMEPNGSVISRSRSESAPGFCPAELMKPEASSAEVLDIDFNCSSPTVMMDNEDEDSFEQKASASNGGDAAAGTQFLKRSLDLSLNYEAIIESWGSSPWTDGQRPNVQLDDFWPHAHLTGWMAGGGRLGGEAAVTPRLGMGGGREARVTRYREKRRTRLFAKKIRYEVRKLNAEKRPRMKGRFVKRPAAAGGGATIAASCAVT

>Sspon.04G0004590-2B_SsBBX7

RPRPLLDVLAKPPPSPLSSRRRSLFSLSARQQRKLLDLSPRSPAVGWLVPRLGRARASGIRAPWRSAARYGYRKVDLVVALNSKWNFSIQSRIMASLCDFCGKQRSMIYCRSDAASLCLSCDRNVHSANALSRRHTRTLLCDRCGSQPASVRCLEDNASLCQNCDWNGHDAASGASGHKRQAINCYSGCPSSAELSRIWSFIMDIPTVAAEPNCEDELSMMTIDDSDVTNHHGASDDKRLLEIANTTLMSDPPSADKLKPLIGFSSGDGFDVLLLATDQPAGPVSVTPKVPYARDDDKFNDGMYEDLCVDDADLTFENYEELFGTSHIRTEELFDDAGIDSYFETKETPPFFNEQPKTMQLECSNVVSADCGMSNPGARADSSLCIPVRQVRSSISHSLSGLTGESSAGDHQDCGVSPMLLMGEPPWHSPGPEGSVAGGSRDSALTRYKEKKKRRKFDKKIRYASRKARADVRKRVKGRFIKAGEAYDYDPLSQTRSY

>Sspon.04G0007300-1A_SsBBX8

MKVQCDVCAAEAASVFCCADEAALCDACDRRVHRANKLAGKHRRFSLLNPAPLSSSGSSAQQAPPPLCDICQEKRGLLFCKEDRAILCRDCDVSVHTTSELTMRHTRFLLTGVRLSAEPAACPAPPSEDENSSGSFCCSAGGGDAAAAPPSAAPATSSHGSGSDNGSSISEYLIKTLPGWHVEDFLVDEAAAAAATNIGVSSADASYLQGGLARIGGRQDGYGYSAWMVQEQFFYEDSAAAAGGGGARGSREQWVPQMAMYSSTGLAGAGSKRSRATSAASSYSY

>Sspon.04G0007300-1P_SsBBX9

MKVQCDVCAAEAASVFCCADEAALCDACDRRVHRANKLAGKHRRFSLLHPCSSSSAAAQKPPLCDICQLLLVFPSNQASLIELGLARMMLYVGRYPSGVGVGVQERRGFLFCKEDRAILCRECDAPVHSASDMTRRHSRFLLTGVHLSSAPVDSAGPSEEEEQEQEQENSSSPCKDACSGAGAGAATTVSASDGSSISEYLTKTLPGWHVEDFLIDDASAGDVVGACSDGLYQGQHGQISGVLQEEAYMPWTGPTDVADERASWERWVPQMHAEFAGGSKRPRASPSPPCSNW

>Sspon.04G0008520-1A_SsBBX10

MDTAVELEQKPAVGYWSVVGARPCDACAAEPARLHCREDGAFLCPGCDDRAHGAGSRHARVWLCEVCEHAPAAVTCRADAAALCAACDADIHSANPLARRHERLPVAPFFGALADAPQPFPSPAFAAAAAAGAQAQGEAAAADDDDGSNEAEAASWLLAEPDNSHEDSAAATAADTLFAESDAYLGVDLDFARCMDGVKAIGVPVAPPELDIAAGSFFYPEHSMIHSLSSSEVAVVPDAQAAGVPAVMSRGKEREARLMRYREKRKNRRFDKTIRYASRKAYAETRPRIKGRFAKRCSAEAEDDALEHDEGACFSPAGSAHAASDGVVPSF

>Sspon.04G0008520-1P_SsBBX11

MEGDEKSAGGAPAYWGLGARLCDACGAEAARLYCRADAAFLCAGCDARAHGAASRHARVWLCEVCEHTPAAVTCRADAAALCASCDADIHSANPLARRHERLPVAPFFGALADAPKPFASSTAVPPKAAPDDDGSNEAEAASWLLPEPDHGHKEGTTTEVFFADSDPYLDLDFARSMDDIKTIGVQGGPPELDLNGAKLFYSDHSMNHSVSSSEAAVVPDAAAGAAPVVAVVSRGLEREARLMRYREKRKSRRFEKTIRYASRKAYAETRPRIKGRFAKRTPGAGADGEDPLEEHEEMYSSAAAAVAALMAPGGADADYGVVPTY

>Sspon.04G0017060-1A_SsBBX12

MEALQVGRYWGVGGRRCGACGASPAAVHCRTCPGGGAYLCAGCDAGHARAGHHRVWVCEVCERAPAAVTCRADAAALCAACDADIHDANPLARRHERVPVQPIGAAAPAAEALLFGAAAEEKDDDDAAAAKVVVDAGKLDFLFADVMDPFFGQDFAGGRFPHADIVVPNNGSSGGAVDLDFGGGVAAAAKPSYSSYTAASLGHSGSSSEVGLVPDVMCGRGGSVTSGVSSLDVGAVPERSDGVMASRVATTPAAAAESREARLMRYREKRKNRRFEKTIRYASRKAYAESRPRIKGRFAKRADDNDADADTDFDTAAAAPAPARSQQQQPSYPYVLDFAAGYGVVPAF

>Sspon.04G0017060-1P_SsBBX13

MELHKYWGVGGRRCGSCEAAPAAVHCRTCVGGSFLCTTCDARPAHARLGHERVWMCEVCELAPAAITCKADAAVLCAACDADIHDANPLARRHARVPVAPIGSEAAAAAVEAMLFGTGEAAAASEADEHNAAAAEQQQHAHAHAHAHALNLNVEAKDMKLDYFFSELDPYLSVEIPRFQHADSVVPNGAGGAAVELDFTCGIGVKHSYSSYTATSLDLAHSGSSSEVGVVPEAFGGGGGSFELDFTRPKPQAYMPYTATPQSHSVSSVDVEVVPERGDLPAVRPVPLMGESREARLMRYREKRKNRRFEKTIRYASRKAYAETRPRIKGRFAKRADHDGDADADDAEAEAAVPSSYVLDFGYGVVPSFA

>Sspon.04G0028530-1B_SsBBX14

MGVAGDAAATASRNRRTGTGTGTRCELCGGAAAVHCAADSAFLCLRCDAKVHGANFLASRHLRRRLPRPPAAAESGAASSASSASSSSCVSTADSAESTAAAPASGAPAAGRAPARRRRPRAEAVLEGWAKRIGFAAGPARCCAAAAAAALRALGRGVAAARVPLRVAMAAALWSEVAPAPAGCRGNGGDAALLRRLEAAAHVPARLVLTVASWMARAASRPVAGPAADADADAQDQEEGWAECS

>Sspon.05G0007690-2C_SsBBX15

MRIQCDACEAAAATVVCCADEAALCARCDVEIHAANKLASKHQRLPLGDGDAAPSLPRCDVCQEKPAFIFCVEDRALFCRDCDEPIHVPGTLSGNHQRYLATGIRVGFSSVCGAAGAEGIPPPAPPKGGSKPAAVGAPAAGGATKTTTVKDTLPQEVPSSPFLPPSDSPLGFKELEWFADIDLFHAHSPAKTTTAEVPEFFASPQPASNAGFYKTNGVARQSKKPRMEVPEDDEDYFIVPDLG

>Sspon.05G0007690-1P_SsBBX16

MKIQCDACEGAAATVVCCADEAALCARCDVEIHAANKLASKHQRLPLEALSAAKLPRCDVCQEKAAFIFCVEDRALFCRDCDEPIHVPGTLSGNHQRYLATGIRVGFASASACSDGACDAHDSDHHAPPKATVEPPPQAAVPAAAQQVPSPPQFLPQGWALHKESPLGFKELEWFADIDLFHEQAPKAGRTLAEVPELFGSQAANDAAYYRPAKAAAGAGVRQSKKARIEVTDDEDYFIVPDL

>Sspon.05G0024260-1B_SsBBX17

MKVQCDVCAAEAASVFCCADEAALCDACDRRVHRANKLAGKHRRFSLLHPCSSSSPAAQKPPLCDICQERRGFLFCKEDRAILCRECDAPVHSASDMTRRHSRFLLTGVRLSSAPVDSAGPSEEEEEQENSSSPCNDGSCSAGAATTASASDGSSISEYLTKTLPGWHVEDFLIDDASAADVVGACSDDLYQYQGQHGQISGVLQEAYIPWTAQEQVLADVVDERASWE

>Sspon.06G0002910-2B_SsBBX18

MSSGGGGGGGGGGQQWPCDYCGEAAAALHCRADAARLCVACDRHVHAANALSRKHVRAPLCAGCAARPAAARVSPVPGADPAFLCADCCEGCDAAMRVPVEGFSGCPSAAELAASWGLDLRRAAVGDDGHGRAEDKGGGDIDLDHEPFLSALDYSVLGVVDPDLRDLYVPCDPPRVPAPDAAGARPLRGEALSDQLAEMARREAGTAHAHPHSDLSPRTPRRTSAASSGRLPPGKMAPPAAMPTHHPPPAAAQEVPPPYTSLLMMASANCADLIGVADRVGDDDEQLLWDCAAPSVLPTQIWDFNLGRSRDHDEKSALEVGYGSNHGGFMIKGYSDMLKEISSGTTKDLEDIYDSRYCSTAEDIMSSNMCQLSSKNVSTASNKRKLSSCASTMDGPTTSGNHVPTSGPALTREISFGDQTVSAPAAERPAVRIDSETLAQNRDSAMQRYREKKKNRRYEKHIRYESRKLRADTRKRVKGRFVKSTEALNAGYGG

>Sspon.06G0008250-1A_SsBBX19

MGSEGSTSPAAGGGAACAVCGGAAAVYCTADAAALCTPCDAAVHAANLLASRHERVPLSMAAVAAASGVYDDLFAPDDIDAASSWPTAPAHGQGQLGSPQNGSSSTSFTTSDSGAEGRSLFDLLSDVDLAAACVTGGGGGYLPDGVAPVHHGAAPLWAQLGLQASAWTTTWSPADAAAAAAVVGVPGAAAAAVVAAAAEREARVQRYREKRKNRKFQKTIRYASRKAYAEARPRIKGRFVKRAAGTSSSSSGAGTSDGNTDATDAASKFWLSFSDDARDDGVGFYVDAGAYGVVPSF

>Sspon.06G0023380-1B_SsBBX20

MSSAAAAGEGGKEKGGAGPGPGGACELCGAAARVYCSADEATLCWGCDAQVHGANFLVARHARALLCRGCARPTPWRAAGPRLGPTASFCDRCVRRGPGAVGVGGADEEMGGAGGGRDDEDDDNGSSDGDDEVVVEDEDEDEDEEGEGENQVVPWTEEAEATPPPVASSTSSSSREAPANGASAAECAKENVPCSTSQPGLCHHGGRSDEATSSRNGGRFLASRHRKRSPSDFFSSGSAQSGSGTPARNCSNAGIGRNDFT

>Sspon.07G0011100-1A_SsBBX21

MKVLCSACEAAEASVLCSADDAALCARCDREVHAANRLAGKHQRLPLLAPGNQSAAAVSPPKCDICQECDAYFFCLEDRALLCRSCDVAVHTANAFVSAHRRFLLTGVQVGQELELDDLSREQPEASPPPPSKSEPAPPPLYSESDFGWAAGAGGATGSLADWSAVEEEFGSPATRLAEAAPRATPKRSPRAPAPAFGAGQGRVAGGVMDWPLGEFFRGVSDFNGGFSFGESGTSKADSGKLGGSAGGSPYYRSSSEDRDANELFGQVPEIQWSVPALPSPPTASGLHWQHGGPDSTAFVPDICSPDGGAVRCFPTADGAAKRQRNR

>Sspon.07G0020490-1A_SsBBX22

MKIGCDACERAEASVLCCADEAALCRSCDAAVHSANKLAGRHHRVALLPSSTAHPSPSPIVDGSGGGGHPACDICQEKTGYFFCLEDRALLCRPCDVAVHAAGAHVSSHRRFLITGVRVGDVESPSHHVPGSDGVSPSASSGNGSCSAPCCSSGGNRMTTTMPDKARPSSSVHATAATTEGSGGQGQQWLWSEFLADDVGVAMEQCCHAELSEPGSSGLTRC

>Sspon.08G0001580-1A_SsBBX23

MQVRCDVCGAAPAAVLCCADEAALCSACDRRVHRANKLAHKHRRIPLLQPASGNDDSSDAAAAAEAPLCDVCKERRGLVFCVEDRAILCPDCDDPIHSANDLTAKHTRFLLVGAKLSAALVDQVPASPDDDDCGRGDGAACEPDAVPAVCAQGSCAAKASALESGGGGGSSISDYLTNICPGWRVDDLLFDDPAFSAASKAGYSDDGHEQVPSLDADLFDVVAGGRPGKRGSAWSGGGGALGFDKVPASVVVDPTAAKQQQGCVRERSWNSDSDSDVFAVPEFPQPPPPKKARPAPAPAPAPTFWCF

>Sspon.08G0009590-1A_SsBBX24

MGALCDFCGEQRSMVYCRSDAASLCLSCDRNVHSANALSRRHTRTLLCDRCASQPAMVRCLAENASLCQNCDWNGHIAGSSAAGHKRQTINCYSGCPSSAELSRIWSFVSDIPNVAPEPNCEQGISMMSISDSGVSNQDNAAGDMLDIASATLISDLGTCDKPLVGSSSGAGVNLLPLATDQTAGSVDSTPDKVPYTPDKDMFSKDSIYEDFCVDDVDLAFENYEELFGTSHIQTEQLFDDAGIDSYFEVKEAPAGNSTEVCSLLMNIVFLNPLQSFTSMCTCTSHSVGVLLLCQSTEPI

>Sspon.08G0021500-2C_SsBBX25

MIATTGSSAKAAAAAVGGKAARACDACLRRRARWYCAADDAFLCQGCDTSVHSANPLARRHERLRLQPASPLRTPPRTGAAAAKRERHDEVVPAWFRRKARTPRGGHAKSVGGQALSRRLVVPHAAAGGDSPDDGRNGEGEFEVVEEEQLLYRVPIFDPALAEFCSPPPPEDAAALASSCNEDGAVEDRAKPDPATPAAAPVVQFFPDGGHANFEPTDAELREFAADMEALLGHGLDDGNEEGSSFYMETLGLLDDPAEVGDDATRVKVETDGGTACEASGTLACALELLDPEGSDEMLLDIDFNYGSPLDTTTDEKAASSDTGAADAQFLQTSLSLTLNYEAIIQSWGTSPWTGGGERPHVKLDDSWPHDYTNMWVVGGVVGHGGEDLLGTARLGMDGGREARVSRYREKRRTRLFSKKIRYEVRKLNAEKRPRMKGRFVKRATAGGSLAIAGLA

>Sobic.001G118100_SbBBX1

MTTSAGAAAGAALGARTARSCDGCMRRRARWHCPADDAFLCQTCDVSVHSANPLARRHHRVRLPSASCSSPPCDPDAPTWLHGLKRRPRTPRSKPGGGKHEATTPNSMAAAASAAVPDLEAEESGSGIVGDNDDHGFLVDDDEDLLYRVPVFDPMLAEFYNPVADEGEQKPLAEFYNLVADEGEQKPACLMPPLVETSPEFASGGLAEADGLSGFDVPDMELASFAADMESLLMGVHDGFDDLGFLDEEKPQVNADAYLEAMAAPVPEREDKKRKRPEMILKLNYDGVIASWVRDGGSPWFHGERPHLDPYELWSDFPAGSRGLLGGAVTAVTGGEREARVSRYREKRRTRLFAKKIRYEVRKVNAEKRPRMKGRFVKRTTLPPLPRPPPQQQQKQLPRALPHVGMVLAPPPGANGRFQF

>Sobic.001G372700_SbBBX2

MGEDDDDQRNQMLGAGLDHEPERRPGEAEPEEGKKPAASEAEAGGDGAGTEAATCDYCGTAAAAVYCRADSARLCLPCDRLVHGANGVCSRHARAPLCADCRAAGAVFRRASSSAFLCSNCDFGRHRDGGDPPLHDRCAVQPYSGCPPASDLAALLGVPLFDKPATEDGGAWWNIWEEPQVLSLEDLIVPTTPCHGFEPLLTPSSPKNRSISPDGKMNEEILRQLGELAESDGGVQASAGREEAEQAGGDQFPSWASPQYATGHGNFGTENNHEVATMPTPLYENGRWNNCDLDALNDACKVEVAYDQVPVSSAEPCLSSFAPLSEICPSISNGNSMEDNHQVNPGIGMPMQGLPKRTGFDVVPCPDRDSVISRYKAKRKTRRFDRQVRYESRKVRADGRLRIKGRFAKANQT

>Sobic.002G273300_SbBBX3

MRTICDVCESAPAVLFCAADEAALCRSCDEKVHMCNKLASRHVRVGLADPNKLARCDICENSPAFFYCEIDGTSLCLSCDMTVHVGGKRTHGRYLLLRQRVEFPGDKPGHMDDVPMETVPMETKDPENQRDQKKAPKEQMANHHNGDHPACDGNCDDQGNIDSKMIDLNMRPVRTHGQGSNSQTQGVDLSVNNHDSPGVVPTSNSERDASK

>Sobic.002G408500_SbBBX4

MSSAAADAASGKEAPACESCTSLPAVVYCRADSARLCLPCDRHVHGANAVSTRHVRAPLCSGCRATATVTAGGGTFLCANCHFGSEEEEGRHRDGDDPQPLHHDRAAVEGYVGCPSIAELAAILGVAGYDEKAAAAGNGGWWPASAWEDPQVLRLEDVIVPTTSCHGLQPLLTPPSPENRSSGGEMADEVVRQLGELAKLEATVAAAYAEMEPADGEQLPPWASPELAIGHADFGALDAGAAWHDAATIAAVPSTEEQEAWIAAGCDVDAAGRTDEEAREHAALAPAPAEPCLSSFVEMSEICPGSVVTLSHGGVGGGGTADVDNSGKTDAETAPRPQLAPTAPVLVAVPVPVTEKMGGYDVAYPDRGTVISRYKEKRKNRRFDKQIRYESRKARADGRMRIKGRFAKSGGEV

>Sobic.003G026700_SbBBX5

MKVLCSACEAAEARLLCCADEAALCARCDRDVHAANRLAGKHHRLPLIPHADVSAPNCDICQEAHAYFFCVEDRALLCRACDVAVHTANAFVSAHRRFLLTGVQVGLQPDAAADADDPNPPTAAAASDPLQTPPPPDRKAAAAGGGSPAPLYSDDDIDWAPGADAGAGVGLPDWALVHEQFSAPPVTRPADPALARTPASKRSPRRSLAAAFTVQSGGGLAGGLPDWPLDEFFGFSEYSAGLGFAENGTSKADSGKLGSTDGSPAGRSSSDASQDFFGQVPEFHQWSVPELPSPPTASGLHWQGGPRHGATTTTDTNTAAVSVPDISSPENPFRCYAATAAGQPPAKRRRRC

>Sobic.004G063200_SbBBX6

MEALVAGRYWGVGGRRCEACGGSPAAVHCRTCPGGGAYLCAGCDAGHARAGHERVWVCEVCERAPAAVTCRADAAALCAACDADIHDANPLARRHERVPVQPIGAAAAAPAAETLLFGAAAEENQDDDDGAAAAAKVVGVDAGKLADFLFADVMDPFFGQDFTGGTRFPHADSVVPNKGSCGGGGAVDLDFGGGVAAAAVAAKPSYSSYTAASLGHSGSSSEVGLVPDAMCGRGGSVTGGVIELDFAQSKAAYLPYAATPTHSMSSLDVGAVPERGDGVMAGRVATPPAAAAAESREARLMRYREKRKNRRFEKTIRYASRKAYAESRPRIKGRFAKRADDNDADADADFDFDAGAAAATAPARSRSQQQQPSYPYVLDFAAGYGVVPTF

>Sobic.004G208400_SbBBX7

MKIQCDACEGAAATVVCCADEAALCARCDVEIHAANKLASKHQRLPLEALSARLPRCDVCQEKAAFIFCVEDRALFCRDCDEPIHVPGTLSGNHQRYLATGIRVGFASASACSSDGACDAHDSDHHAPPKATVETPQAQAAVSAAAAAQQVPSPPQFLPQGWAVDDLLQFSDYESSDKLHKESPLGFKELEWFADIDLFHEQQAPKAGRTLAEVPELFGSQAANDAAYYRPAKAAAGAGVRQSKKARIEVTDDEDYLIVPDLG

>Sobic.004G211200_SbBBX8

MDTAVELELEQKPAVGYWSVVGARPCDACAAEPARLHCREDGAFLCPGCDARAHGAGSRHARVWLCEVCEHAPAAVTCRADAAALCAACDADIHSANPLARRHERLPVAPFFGALADAPQPFPSPAFAAAAAAGGQAQGEAAAADNDDDDGSNEAEAASWLLAEPDNSHEDSAAATAADTLFAESDAYLGVDLDFARCMDGVKAIGVPVAPPELDIAAGSFFYPEHSMNHSLSSSEVAVVPDAQAAGVPAVVSRGKEREARLMRYREKRKNRRFDKTIRYASRKAYAETRPRIKGRFAKRCSAEADDDALEHDEGACFSPAGSAHAASDGVVPSFC

>Sobic.004G249500_SbBBX9

MSSSKHAAAGAGAVGGKAARACDSCLRRRARWYCAADDAFLCQGCDASVHSANPLARRHERLRLRPMTSPPDPAHSTLEAGGVGVASTSTWKKRQQQQQQVAPAWSKRKARTRRPHVKSVGQLLSRKLVVVPEVATVESSEERKVEEEDEEEEEEEEQLLYCVPTFDRALAELCTPPPPLDDPTATASSSCCRDNDVDGAVDNAKAAPPAVVVAESPVQQLPDSFAGFGPTDAELREFAADMEALLGQGLGDSNELDESFYMESLGLMTTTQQAEDVDVGRVKMEPNGSVISRSRGEGAPGFGPAELMKPEASSAEVLVLDIDFNCSSPTVMMDHEDEDSFEHKASASNGDAAAAGTQFLKRSLDLSLNYEAIIESWGSSPWTDGQRPNVQLDDFWPHAHLTGWMAGGGRLGGEAAAVSPRLGMVGGREARVTRYREKRRTRLFAKKIRYEVRKLNAEKRPRMKGRFVKRPAAAGGGGAAIAAPCAVT

>Sobic.004G252300_SbBBX10

MRYQKNGRRYEALGRSSPTARPCDGCHAVQSVVYCHSDAKCLCVSRDKQVHSANQVAERAHVCEVCKSASTVLTCCADAPALCTTCDAKLHSANTLSQRHQRVPVLPLPAAAIQTTSSFDEGKAFVITHGIKEEEEEVDSWLLLTEDSDYSNCTNSTATANNNRNKKMGFGDVDQYFDLSGYNPYYHSNITRNPEEQYMQEQQQIQRRYLEKEWNECAVPSQLTMVYEQQQSVYGIGGAKNAVSVTSSISLSSMEAGIVPDNTIAGISNLNILTTGGVDLLPVRSFQMPVHLSPRDRAARILRYKEKRQARNFNKTIRYATRKAYAQARPRIKGRFTKISDVELKVDLMSSPPDLPNSSYGTVPWF

>Sobic.004G256200_SbBBX11

MASLCDFCGKQRSMIYCRSDAASLCLSCDRNVHSANALSRRHTRTLLCDRCGSQPASVRCLEDNASLCQNCDWNGHDAASGASGHKRQAINCYSGCPSSAELSRIWSFIMDIPTVPAEPNCEDGLSMMTIDDSDVTNHHDASDDKRLLEIANTTLMSDPPSADKPKPLISSSSGDGFDVLPLATDQPAGSVSVTPKVPYARDDDNFNDGMYEDLCVDDADMTFENYEELFGTSHIRTEELFDDAGIDSYFEMKETQPFDFNEEPKTMQLECSNVVSADCGMLNPGARADSSLCIPVRQVRSSISHSLSGLTGESSAGDHQDCGVSPMLLMGEPPWHSPGPEGSVAGGSRDSALTRYKEKKKRRKFDKKIRYASRKARADVRKRVKGRFIKAGEAYDYDPLSQTRSY

>Sobic.004G301000_SbBBX12

MKVQCDVCAAEAASVFCCADEAALCDACDRRVHRANKLAGKHRRFSLLNPAPPSSSGSGSPAQQQAQPPLCDICQEKRGLLFCKEDRAILCRDCDVSVHTASELTMRHTRFLLTGVRLSAEPAACPAPPPPPSGSEDENSSGSGSFCCSAGGDASAAPPPSSAAPATSHGSGSDNGSSISEYLIKTLPGWHVEDFLVDEAAAGAATNIAGVSADASYQGGLARIGGLQDGYGYSAWMAPEQLFYEDSSAAGGARGIREQWVPQMAMYSSSTGLSVAGAGSKRSRATSAASSYSYW

>Sobic.006G131800_SbBBX13

MRIQCDACEAAAATVVCCADEAALCARCDVEIHAANKLASKHQRLPLALGDATAASASSLPRCDVCQEKPAFIFCVEDRALFCRDCDEPIHVPGTLSGNHQRYLATGIRVGFSSVCGAGAGAEGLPPPAPPKGSSKPAAVVSAPAAGATKTTTTVKDTLPQEVPSSPFLPPSGWAVEDLLQLSDYESSDKKDSPLGFKELEWFADIDLFHAHSPAKTTTAEVPELFASPQPASNAGFYKTNGVARQSKKPRMEVPEDDEDYFIVPDLG

>Sobic.006G135100_SbBBX14

MEGDEKSAGGAPAYWGLGARPCDACGAEAARLYCRADAAFLCAGCDARAHGAGSRHARVWLCEVCEHAPAAVTCRADAAALCASCDADIHSANPLARRHERLPVAPFFGALADAPKPFASSAAAVPPKATAGADDDGSSEAEAASWLLPEPDHGHKEEGATTEVFFADSDPYLDLDFARSMDDIKTIGVQGGPPELDLNGAKLFYSDHSMNHSVSSSEAAVVPDAAAGAAPVVAVVSRGLEREARLMRYREKRKSRRFEKTIRYASRKAYAETRPRIKGRFAKRTPGAGEDPLEEHEEMYSSAAAAVAALMAPGGADADYGVDGVVPTY

>Sobic.006G163100_SbBBX15

MKVQCDVCAAEAASVFCCADEAALCDACDRRVHRANKLAGKHRRFSLLHPCSSSSSAAAQKPPLCDICQERRGFLFCKEDRAILCRECDAPVHSASDMTRRHSRFLLTGVRLSSAPVDSAAGPSEEEGEEEENSSSPCNDDSCSGGAGGAGATTTPSASDGSSISEYLTKTLPGWHVEDFLVDDASAGDVGAACSDGLYQQGQRGHISGVLQEEAYTPWTGREQVLGDVADERASWELWVPQMHAEFAGDSKRPRPSPSPPCSYW

>Sobic.007G062100_SbBBX16

MSSAAAGEGGKERGGAGAGPGGACELCGAAARVYCGADEATLCWGCDAQVHGANFLVARHARALLCRGCARPTPWRAAGPRLGPTASLCDRCVRRGPGAVGVGGGDEEMGGAGDGRGHEEEDHDDDGGDDDDEVVVEDDEDEEEEEEEGEGENQVVPWTEEAEATPPPVASSTSSSSREAPANGASAADCAKENMPCSTSQPGLCHHLSSAHHGGRSDEATSSRNGGRFLASSRHRKRSPSDFFSSGSAQSGSGTPARNCSNAGIGRNDFT

>Sobic.007G189800_SbBBX17

MKSGGGGGGGGGGGGQQWPCDYCGEAAAALHCRADAARLCVACDRHVHAANALSRKHVRVPLCAGCAARPAAARVSPVPGADPAFLCAGCCDDAASAAVRVPVEGFSGCPSAAELAASWGLDLRRAEEGKDGAGGDIDDGDPFLSVLDYSVLGVAVDPDLRDLYVPCDPPRVPAPDAAGARPLRGQALCDQLAEMARRETDTAHAHPHSDLSPRTPRRTSAASGGRLPPGKMSPPAAMPTHHPPPAAVQEVPLPYTSLLMMASANCADLIGGADRVGDDDEQLLWDCAAPSVPPTQIWDFNLGRSRDHDEKSALEVGYGSNHGGFMIKSYSDMLKEISSGTTKDLEDIYDSRYCSTAEDIMSSNICQLSSKNVSTASNKRKLSSCASTIDGPTTSGNHVPTSGPALTREISFGDQTVSTPAAERPAVRIDSETLAQNRDSAMQRYREKKKNRRYEKHIRYESRKLRADTRKRVKGRFVKSTEALNAGYGG

>Sobic.008G073400_SbBBX18

MKIGCDACERAEAAVLCCADEAALCRSCDAAVHSANKLAARHHRVALLPSSTAHPPSSTSPIADDGSGSGGGGGDGHPACDICQEKTGYFFCLEDRALLCRPCDVAVHAAGVHVSSHRRFLITGVRVGDVESLSHGVPGSDGGASPSTTSSGNGSSNAPGSSSGGGNPTTTTTTTTMPDQVRPSSSSSIRATAATTAEGSPGQWQQWLWSDFLADDVGGGGGVDMEEECCHAELSEPGSSGLTRS

>Sobic.009G075600_SbBBX19

MKVLCSACEAAEASVLCCADDAALCARCDREVHAANRLAGKHQRLPLLAPGGQSAAAVSPPKCDICQECDAYFFCLEDRALLCRSCDVAVHTANAFVSAHRRFLLTGVQVGQELESDDLSREQQPEASPPPPPSKSEPAPPPPPPLYNESDFGWAAGAGATGSLADWSAVEEEFGSPAPCLAEAAPRATPKRSPRAPPAFGAAGQGRVAGGVMDWPLGEFFRGVSDFNGGGFSFGESGTSKADSSGKLGGSAGGSPYYRSSSEDRDAANELFGQVPEIQWSVPALPSPPTASGLHWQHGGHDSNAFVPDICSPDGGGAGVRCFPTANGAAKRQRNR

>Sobic.010G041700_SbBBX20

MKIQCNACGAAEARVLCCADEAALCVACDEEVHAANKLAGKHQRVPLLTDADAAGTAAAAPAVPKCDICQEASGYFFCLEDRALLCRDCDVAIHTVNSFVSVHQRFLLTGVQVGLDPADPVPPIAEKHVNAAGGSVNQPVKHLPRRSPTVQFSVEGSASVPSKNVTNGDYSRQNSVPTARAEVVDWTMNNSTIRSVESPPKYMSEESPTLLQSSQTTTAFNQINGNSDGPYHLSFSGGNVTDSLPDWPVEEFFSNSEYGPNFGFSEHGSSKGDNAKLGNAGGSPQCRLAEGSVAEELLGQVPGLITDEYMSRVPENSWTVPEVPSPPTASGLNWHGNLCFPAYDSTMFVPEITSLQTSQNQFAVPSSFKRRRREY

>Sobic.010G108500_SbBBX21

MIATTGSSAKTAAAAAVGGKSARACDGCLRRRARWYCAADDAFLCQGCDTSVHSANPLARRHERLRLQPAAASSSPLHTPPRTGAAANNKRERHDEVVPAWFRRKARTPRGGHAKSVGGQALSRSRRLGVVVPHAAAGGGDSPDDGRSAEGEFEAEEEQLLYRVPIFDPALAEFCSPPPAPLEDAAALASSCNEDGAVEDPANSKPDPGPATPAPAPVVQFFPDSGHANFEPTDAELREFAADMEALLGHGLDDGNEEDSSFYMETLGLLDPVEVGDDATRVKVETDGGSACGEASGTLACALELLDPAEVSDEMLDIDFNYGSPLDTMMDDEKAASSDTGGADDAQFLQTSLSLTLNYEAIIQSWGSSPWTAGGERPHVKLDDSWPHTNMWVVGGVAGHGGEDLLLGTARLGMDGGREARVSRYREKRRTRLFSKKIRYEVRKLNAEKRPRMKGRFVKRATAGGSSLAIAGLA

>Sobic.010G115800_SbBBX22

MNYNFSSNALDEEEVAGRGGEGGSCAAAPAWARPCDGCRAAPSVVYCHADAAYLCASCDVRVHAANRVASRHERVRVCEACERAPAVLACRADAAALCVVCDAQVHSANPLAGRHQRVPVLPLPVAAIPAASVLAEAAATAVAVGDKQEEEVDSWLLLTNTKDPVSDNNNCNCSSSSNNNISSSNTSTFYADVDEYFDLVGYNSYCDNHINSNPKQYGMQERQQQQQLLLQKEFGDKEGSEHVVPASQVAMANEQQQSGYGVIGVEQAASMTAAVSAYTDSITNSISFSSSMEVGIVPDNMATTTDMPNSGILLTPAEAISLFSSGSSLQMPLHLTSMDREARVLRYKEKKKSRKFAKTIRYATRKTYAEARPRIKGRFAKRSSDMEIEVDQMFSSAALSSDGSYGTVLWF

>Sobic.010G123500_SbBBX23

MGALCDFCGEQRSMVYCRSDAASLCLSCDRNVHSANALSRRHTRTLLCDRCASQPAMVRCLAENASLCQNCDWNGHIAGSSAAGHKRQTINCYSGCPSSAELSRIWSFVSDIPNVAPEPNCEQGISMMSISDSGVSSQDNAAGDNNLLDIASETLISDLGTCDKPLVGSSSGAGVNLLPLATDQTAGSVDSPPDKDSCVQVPYTPDKDMFSKDSIYEDFCVDDVDLAFENYEELFGTSHIQTEQLFDDAGIDSYFEVKEAPAGNSTEQSKLKQPANSNAVSADSGMSNPGVKGDSSVCIPLRQARSSLSLSFSGLTGESSAGDHQDCVVSSLLLMGEPPWQPPGPEGSIAGGSRDSAITRYKEKKKRRKFDKKIRYASRKARADVRKRVKGRFVKVGEAYDYDPLCQTRSY

>Sobic.010G214000_SbBBX24

MELHKYWGVGGRRCGSCEAAPAAVHCRTCVGGSSSFLCTTCDARPAHARLAHERVWVCEVCELAPAAVTCKADAAVLCAACDADIHDANPLARRHARVPVAPIGSEAAAAAVEAMLFGTGDAAEADDQHNNAAAAAEQHQHQHHAHHAHALNLNVEAKDMKLDYLFSELDPYLSVEIPRFHHADSVVPNGAGAAGAVELDFTCGIGVKHSSYSSYTATSLDLAHSGSSSEVGVVPEAFGGGGGGGGGSFELDFTRPKPQAYMPYTATPQSHSVSSVDVEVVPERGDLPAVRPVPLMGESREARLMRYREKRKNRRFEKTIRYASRKAYAETRPRIKGRFAKRADHDGDGDADDAEAEAAVPSSYVLDFGYGVVPSF

>Sobic.010G262200_SbBBX25

MQVRCDFCGAAPAAVLCWADEAALCSACDRRVHRANKLVHKHRRIPLVQPASGNVSDADADAAAPLCDVCKERRGLVFCVEDRAILCPDCDDPIHSANDLTAKHTRFLLVGAKLSAELVDQAPASPDDDDDDDDACGRDTRAAAEPDAVPALGAQGSCAAKASALESGSVGGGSSISDYLTNICPGWRVDDLLFDDPAFSAASQKASGYSDDGHEQVPSLDADLFDVVAGGRPGKRGGVWSTGAGALGFDKATPASVVAVPTQGFVREMSWNSDSDSDVFAVPEFPHPPPAKKARPAPASTFWCF

>Zm00001d029149_ZmBBX1

MGEGDDDRRDELLGAGRDHEPEPADAEEAKKPAPGEAEAGNGAGAEAATCDYCGTAAAAVYCRADSARLCLPCDRLVHGANGVCSRHARAPLCAGCCAAGAVFRRASTSAFLCSNCDFGRHRDGGDPPLHDRCAVQPYSGCPPASDLAALLAVPLFDKPAAEDGAWWNIWEEPQVLSLEDLIVPTTPCHGFEPLLTPPSPKNRSISPDRKVNEEILRQLGELAESDGGMQASAGREEAEQAGGDQFPSWASPQYATGHGNFGTEDNHENGRWNNSEYHDLNDACKLEVTYDQAPVNSAEPCLSSFAPLSEICPSMSNGSSKEDNHQANPGIGMPMQGLPKRSGFDVVPCPDRDSVISRYKAKRKTRRFDRQVRYESRKVRADGRLRIKGRFAKANQT

>Zm00001d031662_ZmBBX2

MKSCGGGGADGQQCPCDYCGEAAAALHCRADAARLCVACDRHVHAANALSRKHVRAPLCAGCAARPAAARVSLGADPAFLCADCCEGCAAASAARVSVEGFSGCPSAAELAASWGLDLRRAAVAVGDDGDGGDDDDPFLSVLDYSVLGVGVADTDLRDLYVPCDPPRVPVPDAGARPLRGEALCDQLAEMARRDEADTSHAHPHSDLSPRTPRRTSAASSGRLPSGKMAPPAALPVPAHPPPAAPQEVPLPYTSLLMMASANCSDLIGGGDRVGDTDEQLLWDCAAPSVPPTQIWDFNLGRSRHHDEKSALEVGYGSNHGGFMIKSYSDMLKDISSGTTKDLEDIYDSRYCSTAEDIMSSNICQLSSKNVSTGSNKRKVRSCAASTMDGPTTSGNHNHVPASASGPGAALTREISFGDQTVSAPAAETERPAAVRIDSETLAQNRDSAMQRYEKHIRYESRKLRADTRKRVKGRFVKSTEALNAARYNG

>Zm00001d033719_ZmBBX3

MTNAGAATGAALGARTARSCDSCMRRRARWHCPADNAFLCQSCDVSVHSANPLARRHHRVRLPSASCSSPPRDPDAPTWLHGLKRRPRTPRSKPGGSNKHEAAPSSIAAAASAAVPDLEAEAEAEAEESGSGILGDNDDDHGFQDDDENLLYCVPVFDPMLAEFYNPVADEGEQKPACLMLPLVETSPEFASGGLAEADGLSGFDVPDMDLASFAADMESLLMGVDDGFDDLGFLDEQKPQVNADVDLEAMAAPEPEREDKKRKRDGFDYLGFLDEEKPQVNADVDLEAMVAPEPEREDKKRKRTDMILKLNYEGVIASWVRDGGSPWYHGERPHLDDPYELWLEFPATGSRGLFGGTMTAVTGGEREARVSRYREKRRRRLFAKKIRYEVRKLNAEKRPRMKGRFVKRTTLPPLPRPPPPSQQQQKKKQLPRSLPHVGMRALVPVTVPAGTSAQGRGALAHIFRLAISTN

>Zm00001d002806_ZmBBX4

MKVQCDVCTAEAASVFCCADEAALCDACDRRVHRANKLAGKHRRFSLLHPCSSSSSSAAHKPPLCDICQERRGFLFCKEDRAILCRECDAPVHSASDMTRRHSRFLLTGVRLSSAPVDSAGPSEEEEQENSRGPCNDESCSSGSGAGGATTATASDGSSISEYLTKTLPGWHVEDFLIDDASAGDVGACSDGLYQGQNGQISGVLQEAYLPWTEREQVQTDVADERASWERWVPQMHAEFGGGGKRPRASPSPPCSYW

>Zm00001d003162_ZmBBX5

MEGDEKSAGGAPAYWGLGARPCDACGAEAARLYCRADAAFLCAGCDARAHGAGSRHARVWLCEVCEHAPAAVTCRADAAALCASCDADIHSANPLARRHERLHVAPFFGALADAPKPFASAAPPKATDDDGSNEDEAASWLLPEPDHGQKEGATTEVFFADSDPYLDLDFARSMDEIKTIGVQQSGSPELDLAGTKLFYSDHSVNHSVSSSEAAVVPDAASGMAPMVAVVSRGLEREARLMRYREKRKSRRFEKTIRYASRKAYAETRPRIKGRFAKRTPGAGEDTLEEHEEMYSSAAAAVAALMAPGGADADYGVVPTY

>Zm00001d006198_ZmBBX6

MTLAAGAARRWDADDLRCVRERTGGALLCGRRGCALPALRREVLILLIADMCMAIEDYPFSQERISKAFTVHMCNKLASRHVRVGLADPNKLARCDICENSPAFFYCEIDGTSLCLSCDMTVHVGGKRTHGRYLLLRQRVEFPGDKPGHMDDVPMEIKDPENQREQNTPKEQMANHHNVNDPVSDGNCDGQGNIDSKMIDLNMRPARTHGQGSNSQELLGAEMGLFSGLSVITSKRISGRVGANNCISIRLVVP

>Zm00001d007107_ZmBBX7

MSDADAAGSKEAPRCDYCMGLPAVVYCRADSARLCLPCDRHVHGANTVSTRHARAPLCARCRAAAATAVASPRGGGGGFVCADCYCLEKEDEEKGEEHRDPRPLHHDHDAVEEYAGCPSIAELAAILGVAGYDEKAAAAGGDVGWWSTWEEPQVVCLEDVVVPTTSCHGLHPLLAPPSPKNRSSGGELADVVIRQLEELAKSEAAAVAASYAELEPGDGEQLPPWASPELDIGAAADFGALDAADADAAWHDAATMAFAAVPSHEEQEAWIATACDVDARRAEVEVEEAREQAAPAPDEPCLSSFVDMSEIFPASVTLSSGGDVDNSGNKPDEETAPRPQLLATTALVPVTEKKGGYDVAHPDRGTVISRYKEKRKNRRFGKQIRYESRKARADGRMRIKGRFAKSGEV

>Zm00001d039437_ZmBBX8

MKVLCSACEAAEARLLCCADEAALCARCDRDVHAANRLAGKHHRLPLLPPDDVSAPNCDICQEAHAYFFCVEDRALLCRACDMAVHTANAFVSAHRRFLLTGVQVGLQPAAAAQDADPHPPAAAEPLQTPPPPDRKAAAAGDGSSPALLYSDDDIDWAAGADAGGGVSVAVTLPDWSLVHEQFGAPPVVPRHADPALARTPSSKRSPRRSVAAAFTVQGGGGLAGGLPDWPLDEFFGFSEYSAGLAFAENDTSKRSVFHWQADSGKLGSTDGSPAGRPSSDASQDFFGQVPEFHQWSVPELPSPPTASGLHWQGGPRHGAATTTDVAAVFVPDISSPENPFWCYATAAGQAPTKRRRRC

>Zm00001d049347_ZmBBX9

MGGEGSTSPAPGGAACAVCGGAAVVYCVADAAALCSPCDAAVHAANLLASRHERVPLSMAAVPSASGAYDDLFAPDDVDAAAPAQAQGLGSPRNGSSSASFTTSGDSGAEGSSLFDFDLLSGVDLAACVTDGVAPLHHDDAAPLWAQPGLAVAWATAWSPADSAAAVVAAAAEREARVQRYREKRKNRRFHKTIRYASRKAYAEARPRIKGRFVKRAAGTSSSSSSGAGTPDDGNDATGAAAKFWLSFSDDARDGFYVDAGAYGVVPSFYRVRSEQLTS

>Zm00001d051018_ZmBBX10

MKIQCDACEGAAATVVCCADEAALCARCDVEIHAANKLASKHQRLPLEALSASLPRCDVCQEKAAFIFCVEDRALFCRDCDEPIHVPGTLSGNHQRYLATDIRVGFASASSACSDACDAHDDSDHHAPPKAAVSSAAQQVPSPPQFLPQGWAVDELLQFSDCESSDKLHKESPLGFKELEWFTDIDLFHEQTPKAGRRLAEVPELSGTQAANDAAYYRPAKATATAGAGVRQSKKARTEVTDDEDHLIVPDLG

>Zm00001d051047_ZmBBX11

MELEQKPPPPPAGYWSGVAGGRPCDACAARPARLHCRADGAFLCPGCDARAHGAGSRHARAWLCEVCEHAPAAVTCRADAAALCAACDADIHSANSLARRHERLPVAPLLGALSDAPAPQHFPSAAAAAGEEASAAEEEDGSDEAEAASWLLPEPDNSHEDSAAADSFFAEPDAYLGVDLDFARCVDGVKAIGVPVTPAPLELDMAAGSFFYPQHSMNHSIVQVPSSSEVAVSRGKEREARLMRYREKRKSRRFDKTIRYASRKAYAETRPRVKGRFAKRCSEDDDALEHEEAACFSPAGSASASSDGVVPSLC

>Zm00001d051309_ZmBBX12

MKVQCDVCAAEAAEVFCCADEAALCDACDRRVHRANKLAGKHRRFSLLSPAPPPPPPLCDICQDKRGLLFCKEDRAILCRDCDVSVHTASDLTMRHARFLLTGVRLSAEPAAACPAPEDEEEEDDENSSGSFCCSAGDAAAHPPPLPSSAPATSHGSDSSSISEYLTKTLPGWHVEDFLVDEAAAAAATDIGVSAADASYQISFPPHYLIGGLQDGYPAWMAAQERLLCEGGGGARGSRERWVPQMATYSGPGLAVAVAGTNKRSRATSAASSFPYW

>Zm00001d051610_ZmBBX13

MQVLCDVCGSAPAAVLRCTGEAALCSACDRRVHRADKRRRIPLVHPCGDDSTAAAPLCDVCKEWRGLVFCMEDRAILCPDCDDPIHSAYDLTAKHTRFLLVGAKLSATLVDQAPPSPDDDDDDDDVAKPDAAPAACAAKAYREEALPHSRNHDKPVVLKDLRWGYYLTDEPVVHFVWQPNQESSVLVMPRSFDDYYIENCGVILAPEVTQRRIDNSDQFVILATVRATDRNRHETCNAIPTKFRLQTSGLSGHRAKRQSDGAATVTSPAAKRARDPSAPAFPTNKEVPDMPPKTRIFCDILASRAAFFRWAGHRHLGHEHSPYSWNLFVDILGKNRLFEPMWDTVKSMHSQQLLSLATFASVFSSLAATPGGSPLKAFMDMPRYGMTRDTAALNSLLSALCRANRFDDARAAIPVAALRLARARMPTPTPSSSRAASLPPTCGLRASDSSTAPPEAMGYLNEAPRGSANI

>Zm00001d051684_ZmBBX14

MVPLCGFCGKQRSMIYCRSDAASLCLSCDRSVHSANALSRRHRRTLLCDRCGLQPASVRCLEDNTSLCQNCDWNGHDAASGASGHKRQAINCYSGCPSSAELSRIWSFIIDIPTVAAEPNCEDGLSMMTIDDSDVTNHHGASDDKRLLEIANTALMSDPPSPDKLKPLIGSSSGDGFDVLPLATDQPAGPVSATPKVPYARDDNKFNDGMYEDLCVDDADLTFENYEELFGTSHIRIEELFDDAGIDSYFEMKETPPFDFNEQPKIVQLQCSDVVPADCAMSNTGERADSSLCIPVRQVRSSISHPLSGLTGESSAGDHQDCGVSPILLMGEPPWYSPGPEGSLAGGSRDSALTRYKEKKKKRMFDKKIRYASRKARADVRKRVKGRFIKAGEAYDYDPLSQTRSY

>Zm00001d013443_ZmBBX15

MTSAGAATGAALGARTARSCDGCMRRRARWHCPADDAFLCQTCDVSVHSANPLARRHHRVRLPSASCSSPPRDPDAPTWLHGLKRRPRTPRSKPGGSKSNKHEATPSFIAAAASSAAVPDLEAEESGSGILGGNDDHHGFLQDDDEDLLYRVPVFDPMLAEFYNPVADEGEQKPACSLLMPSLAETSPEFASGGSAEADGLSVSFHVPDMELASFAADMESLLMGVDDDGFDCLGFLDEEKPQVNADLDAIVAPAPEPEDKKRKRPEMILKLNYEGVIASWVRDGGSPWFHGERPHLDCHELWSDDFTTGSRELLGGAVTPVTGGEREARVSRYREKRRTRLFAKKIRYEVRKLNAEKRPRMKGRFVKRATLPPLPRPPPPQQQQQQKQLPRAPPHVGMVLPPPPVSNGRLWF

>Zm00001d014765_ZmBBX16

MQMLCDVCAAAPAAVICCADEAALCSACDRRVHRANKLAHKHRRIPLAQPSGDESDADAKPLCDVCKERRGLVFCVEDRAILCPDCDDPIHSANDLTAKHTRFLLVGAKLSAALVDAQAPHSPDDDDNDCGRGNGAAAEPDAVPAVCAQGSCAAKASSLESGGGGGGGSGSGSSISEYLTNICPGWRVDDLLFDDSAFSAASVSSSPCSVLVFLYPQPYHANVRIGLSRRFERKQKADSCDDGHEQVPSLDADLFDVVAGAGWPGKRGSAWSGVGALGFDKVPASVVVVPTAAKQQQGCVRERTWDSDSDSDVFAVPELPQPPQAKKARPAPAPAPTFWKKITGPLGPTGSESTI

>Zm00001d015434_ZmBBX17

MGAARDSAAAGQKHGTGTRCELCGGAAAVHCAADSAFLCLRCDAKVHGANFLASRHVRRRLVPRRAADPEASSAASSGSSCVSTADSAESAATAPAPCPSRTAGRRAPARARRPRAEAVLEGWAKRMGFAAGPARRRAAAAAAALRALGRGVAAARVPLRVGMAGALWSEVAAGCRGNGGEEASLLQRLEAAAHVPARLVLTAASWMARRPDARQEDHEEGWAECS

>Zm00001d017176_ZmBBX18

MDTAAELELGLELEQKPAAGYWSVVGARPCDACAAEPARLHCRADGAFLCPGCDARAHGAGSRHARVWLCEVCEHAPAAVTCRADAAALCAACDADIHSANPLARRHERLPVAPLFGALADAPQPFPSPAFAAAAGAEAPAQGEAVAEDYGSSEAEAASWLLPEPDNSHEDSAADTFFAESDAYLGADLDFARCMDGVKAIGVPVAPPELDIGAGSFCYPEHSMNHILSSSSEVAVVPDAQAAGLPVVVVVSRGEEREARLMRYREKRKNRRFDKTIRYASRKAYAETRPRIKGRFAKRRSAEGEDEALEHEEGACFSPTGSAPAASDGVVPSFC

>Zm00001d017412_ZmBBX19

MKVQCDVCAAEAASVFCCADEAALCEACDRRVHRANKLAGKHRRLSLLSPAAPSSSAQQTPPPPLCDICQEKRGLLFCKEDRAILCPDCDVPVHTASELAMRHTRFLLTGVRLSGSPQSPPRARRRRRRTRTTAAAAASPST

>Zm00001d017885_ZmBBX20

MEVKYRIGFHWIQLLSRVLEGGFDTSSEQPVEFLKYCPGSWLLSVISVGNKANALSRRHTRTLLCDRCGSQPASVRCLEDNASLCQNCDWNGHDAESGASGHKRQAINCYSGCPSSAELSRIWSFITDIPTVAAEPDYEDGLSMMTIDGSDVTNRHDTSDDKRLLEIANTTLMSDPPSADKLKSPTGSSSGDGFDVMTLATDQPAGPVSATPKVPNARDDDKFNDGMYEDLCVDDADLTFEDYEELFGTSHIRTEELFDDAGIDGYFELKETPPFYFNEVCYLTLSMHTTVISGWPKRIFSFEFFLWQRELLCLLLQPKAMQIECGNVVSADCAMSNPGARADSSLCIPVRQVRSSISHSLSGLTGESSAGDHHDCGVSPMLLMGEPPWHSPGGPEGSVAGGSRDSALTRYKEKKKRRKFDKKIRYASRKARADVRKRVKGRFIKAGEAYDYDPLSQTRSY

>Zm00001d017939_ZmBBX21

MSSSKHAAGAVGGKAARACDSCLRRRARWYCAADDAFLCQGCDASVHSANPLARRHERLRLRPTDPHSTTLEAGVATATWKKRQQQVAPAWSKRKARTRRPHVKSVGELLSRKLVVVPEVSPIESSEERKAEEEEEEEEGQLLYCVPTFDRALAELCSPPPPVDDPTASSCCRDDVDGAVENNTKAPPVVVAESPVQQLPDSFAGFGPTDAELREFAADMEALLGQGLDDGNELDRSFYMESLGLMAQQAEDVGRIKMEPNGIVSSRSRGEGAPGFGPTEMKPEASSAAAEVLDTDFNCCSPTVMMDNEDEDSFEQKASASNAAAAAAGTQFLKRSLDLSLNYEAIIESWGSSPWTDGQRPSVQLDDFWPHAHLTVCVQGWMAGGGRLGGEAAVTPRLGMGGGREARVTRYREKRRTRLFAKKIRYEVRKLNAEKRPRMKGRFVKRPAAAAGGGGEELPLPPPNL

>Zm00001d036214_ZmBBX22

MRIQCNACGAAEARVLCCADEAALCVACDEEVHAANKLAGKHQRVQLLTDSATAAASPAPAVPKCDICQEASGYFFCLEDRALLCRDCDVAIHTVNSFVSVHQRFLLTGVQVGLDPADPVPPIAEKHVNASGGSVKQSVRHLPRRSPGVQFSVEGSASVPSKNVSNGDYSRQNSVPTARAEVVDWTKNNTTIQSVESPPKYMSEESPTLLQSSQTTTVFSNQINGNSDGTYHLSFSSGNVTDSLPDWPVDEFFSNSEYVPNLGFSEHGSSKASCHTIVLSLQGDNAKLESAGGSLQCRLAEGSIAEELLGQVPGLITDDYMSRVPENSWTVPEVPSPPTASGLNWHGNLCFPAYDSTMFVPEITSLQNSQNQFTVPSSFKRRRREY

>Zm00001d036418_ZmBBX23

MQVLCDVCGGAPAAVLFCTDEAALCSACDRRVHRADKRRRIPLVQPCGDDSAAAAAAPLCDVCKERRGLVFCVEDRAILCPDCDDPIHSANDLTAKHTRFLLVGAKLSAALVDQAPPSPDDDDDDVAEPDAVPAACAAKASSLESGGGSSISDYLTNICPGWRVDDLLLDDSSFSAPSKTGYSDGHDQVPSVDADLFDVVASGRPGKRVGGAALGFDKAPASVVVVPTQGCVTERTWNSDSDVFAVPELPQPPPAKKARPAPAPTFWCF

>Zm00001d036676_ZmBBX24

MGHRRHEGSCRCELCGAPAAVHCAADEAFLCAACDAKVHGANFLASRHRRTRLRLAAAGPPPPDDEAGYGSAASSCVSTADSAPRPRRGARAPAGSVRPEAVLEAWAKRMGLGAGARRRVPPRVAVAAALWWEAAGRGGVGGHAQALRRLEACAHVPARLVLAVAASLARARALLRRAATDAVEGWDECAWAGPKSSPSRS

>Zm00001d037327_ZmBBX25

MIATTRGSSAKAAAAVGGKAARACDGCLRRRARWYCAADDAFLCQGCDTSVHSANPLARRHERLRLCPASPLQTPPDRSAAAAATATNKRERHDEVAVPAWFGRKARTPRGGHAKSVGQVALSRRLVVPHAAGGDSDSPDERNGGEEEQLLYRVPILDPALAEFCSPPPLEDAAGLALDASVCNEDGAIEDPAKPDPAAPLAQFCPVSGHFNFGPTDAELREFAADMEALLGHGLDDGNEEDSSFYMETLGLLDPMEVGDDATQVKVETDGSSACCEASGTLACGLELDLEASDEMLDIDFDYASPQDTATDERAASSDTGADAQFLQTSLSLTLNYEAIIQSWGSSPWTGGGERPHVKLDTRWPHDYTNMWVVGGVVGHGGEDLPGTPRLGMDGGREARVSRYREKRRTRLFSKKIRYEVRKLNAEKRPRMKGRFVKRATAGGSLAIAGLA

>Zm00001d037735_ZmBBX26

MKVLCSACEAAEASVLCCADDAALCARCDREVHAANRLAGKHQRLPLLAPGGQGAAAVSPPKCDICQECDAYFFCLEDRALLCRSCDVAVHTANSFVSAHRRFLLTGVQVGQELEPDDLSLEQREASSPPPAKSEPTPPLYSESDFGWAAGAGATGSLTDWSAVQEEFGSPAPRLAEAAPRATPKRTPRAPAFGAGQGRIAGGVMDWPLGEFFRGVSDFNGGFSFGESGNSKADSGKLGDSAGGSPYYRSSEEERDANELFGQVPEIQWSVPALPSPPTASGLHWQHGGPDSGAFVPDICTPDGAGRCFPTASGAAKRQRNR

>Zm00001d021278_ZmBBX27

MRTICDVCESAPAVLFCAADEAALCRPCDEKVHMCNKLASRHVRVGLADPNKLVRCDICENSPAFFYCEIDGTSLCLSCDMTVHVGGKRTHGRYLLLRQRVEFPGDKPGHMDDVPMEIQDPENQRDQKKPPKEQTANHHNGDDPATDGNCDDQGNIDSKMIDLNMRPVRTHGQESNSQTQGVGLSVNNHDSPGVVPTSNSERDTSK

>Zm00001d045323_ZmBBX28

MKIQCNACGAAEARVLCCADEAALCVACDEEVHAANKLAGKHQRVPLLTDAAAAAAAAAPAVPKCDICQLYLDVANRLQGLMRLAQGANKQQPYPELLSLFEASGYFFCLEDRALLCRDCDVAIHTVNSFVSVHQRFLLTGVQVGLDPADPVPPIAEKHVNAVGGSVNQPVRHQPRRSPTVQFSVEGSASVPTKNVTNGDCSRQNFVPTARAEVVDWTMNNSTIRSVESPPKYISEESPTLLQSSQTTTVFSNQINGNSDGAYHLSFSGGNVTDSLPDWPVDEFFSNSEYGPNFGFSENGSSKAKLESDILSGIYMKKGDTAKLGGAGGSPQCRLAEGSVAEELLGQVPGLITDEYMGRVPENSWTVPEVPSPPTASGLNWHGNLCFPAYDSTMFVPEITSLQNSQSHFTVPSSFKRRRREY

>Zm00001d045661_ZmBBX29

MIATTTTGSSAKAAAVGGKEARACDACLRRRARWYCAADDAFLCQVCDTSVHSANSLARRHERLRLRPTSPLQTPPPPTPASANRESHDEVVPAWFKRKARTPRGGRAKSDVRTLSRRLVVPHAAGGDSPDGRNDEGEFEAEEPEEEVLYRVPVFDPALAEFCSPQPLEDAAALASSCNEDGAVEDPAKTDRETPAAAPLVQFFPDGGHANFGPTDAELREFAADMEALLGYGLDDGNEESSSFCMETLGLLEPVEVGEDASRVKVETDAGSACEASGTLACALELLDPDASDEMLDIDFNYGSPQDTTTTENAASSSHTGTDGQFLQTSLSLTLNYEAIIQSWGSSPWTGGAERPHVKLDDSWPHDCTNMWVVGRGMVGHGGEDLLGTPRLGQGMDDVGREARVSRYREKRRTRLFSKKIRYEVRKLNAEKRPRMKGRFVKRATAGGSLTIAGLA

>Zm00001d045735_ZmBBX30

MTPSVTPWIPLAPTLLLVLCTGLAALWIHHCNFMDYNFDTSVLDEDVAGRGGREGSCPPAWARACDGCRAAPSVVYCHADTAYLCASCNSRVHAANRVASRHERVRVCEACECAPAVLACRADAAALCAACDAQVHSANPLAGRHQRVPVLPLPAAAVPAASVLAEAAATAAAVAGDKDEEVDSWLLLTKDPDDDDKNHNCSSNNNNISSNTSTFYADVDEYFDLVGYSSYCDNHINSNTKQYGMQEQQLLLHKEFGDKEGSEYVVPSQVGQQQSGYHRVIGTEQAASMTPGVSAYTDSISNSISFSSSMEVGIVPDNMATTDMPSSGILLTPAGAISLFSSGPPLQMPLHLASMDREARVLRYREKKKSRKFEKTIRYATRKTYAEARPRIKGRFAKRSSDMDVEVDQMFSAAALSSDGSYGTVPWF

>Zm00001d045804_ZmBBX31

MGALCDFCGEHRSMVYCRSDAASLCLSCDRNVHSANALSRRHTRTLLCDRCASQPAMVRCLAENASLCQNCDWNGHIAGSSSAGHKRQTINCYSGCPSSAELSRIWSFVSDIPNVAPEPNCEQGISMMSISDSGVSNQDNAAGDSILLDIASATLVSDIGTCDKLLVGSSSGAGVNLLPLATGQTETAGSVDSTPDKVPYTPDKDMFSKDSIYEDFCVDDADLAFENYEELFGTSHIQTEQLFDDAGIDIYFEMKEAPAGNSTERIWFGQIRRCVFVFYYMIIIGFLSHAFQQSKLKQPANSNAVSADSGMSNPGVKGDSSVCTPLRQARSSLSLSFAGLTGESSAGDHQDCVVSSLLLMGEPPWQPPGPEGSIAGGSRDSALTRYKEKKMRRKFDKKIRYASRKARADVRKRVKGRFVKAGEAYDYDPLCQTRSY

>Zm00001d046925_ZmBBX32

MELHKYWGVGGRRCGSCEGAPAAVHCRTCVGGSFLCTTCDARPAHARLGHERVWMCEVCELAPAAVTCKADAAVLCAACDSDIHDANPLARRHARVPVAPIGSEAAAAAVEAMLFGTGEAAASEADEQHAAAEHAHAHAHALNLNVEAKDMKLDYLFSELDPYLSVEIPRFQHADSVVPNGAGAAVELDFTCGIGVKHSSYSSYTATSLAHSGSSSEVGVVPEAFGGSGSGGGSFELDFTRPKPQAYMPYTGTPQSHSVPSADVEVVPERGDLAAVRPVPLMGESREARLMRYREKRKNRRFEKTIRYASRKAYAETRPRIKGRFAKRADHDGDADADDAEAEAEAAVPMSYVLDFGYGVVPSF

>Zm00001d024200_ZmBBX33

MGRSEGSTSPAAGGAACACAVCGGAAVVYCAADAAALCSPCDTAVHAANLLASRHERVPLSMVTAASGVYDDDLFAPDDIDAASSWASAPAQGQGSPQNGSSSASFTTGDSGAEGRGLFDLLSDVDFAACVTGGGGYLPDGVAPVVHHGAAPLWAQPGLAAAAWTATWSPAVVVPGAAVVAAAAAEREARVQRYREKRERRKFQKTIRYASRKAYAEARPRIKGRFVKRAAGTSSSSSSGAGTSDSIDAAAKFWLSFSDDARDGFFVDAGAYGVVPSF

>Zm00001d024213_ZmBBX34

MSAAWEGGKEKGAATPAPGGACELCGAMARVYCGADEATLCWGCDAQVHGANFLVARHARALLCRGCARPTSWRAAGPRLGPSASLCDRCVRRGSGAVGVGGDVEMGGTGDGRGDEEEEEDDDEEDDDVVVEDDDDEEEGEGENQVVPWTEEAEATPPPVASSTSSSSREAPANGASGGECAKENMPCSTSQTGLLCHNSSSARHGGRSDEVTSSRNGGRFLASGHRKRSPSDFFGSASAQSGSGPPAMNGSNAGIGRNE

>Zm00001d025770_ZmBBX35

MKGDEKSAGGAPAYWGLGARPCDACGAEAARLYCRADAAFLCAGCDARAHGAGSRHARVWLCEVCEHAPAAVTCRADAAALCASCDADIHSANPLASRHERLPVAPFFGELADAPKPFASSAAVPKAADDDGSNEAEAASWLLPEPDHGQKEGATTEVFFADSDPYLDLDFARSMDDIKTIGVQGGPPELDLAGAKLFYSDDSMNHSVSSSEAAVVPDAVAGAAPEVAVVCRGLEREARLMRYREKRKSRRFDKTIRYASRKAYAETRPRIKGRFAKRTPGAGADGEEPLEEHEEIYSSAAAAVAALMAPGGADADYGVVPTH

>Zm00001d025957_ZmBBX36

MKVQCDVCAAEAASVFCCADEAALCDACDDRVHRANKLAGKHRRFSLLHPCSSSAQKPPLCDICQERRGFLFCKEDRAILCRECDAPVHSANDMTRRHSRFLLTGVRLSSAPVDSADPSEGEEEEEQENSSRPGNGESCSGGAGATTATASDGSSISEYLTKTLPGWHVEDFLVDDAYASDVGACSSDGLYQGQQDGQISGVLQEAYMPWTGRELVPADVADERANWERWVPQMHAEFAGDSKRPRASPSPPCSYW

>LOC_Os01g10580_OsBBX1

LCSACEAAEARVLCCADDAALCARCDLHVHAANRLAGKHHRLPLLSSSSSSSSPSPPTCDICQDAHAYFFCVEDRALLCRACDVAVHTANALVSAHRRFLLTGVHVGLDAAADDDDKHPPHPLSSSLPRNTAPPPQPPPKRSPSPIYSDDDVIDWATGGHDIGITGNLPDWSLVDEQFNTPALPPVVTKTPPKRASRGPVTAGTAAAVFGNLAGGSPDWPLNEFFGFADFSSGFGFAENGTSKADSGKIGSMDGSPNGGRSSSSSSSSSAAAAGGGGGGQDFFGQVPEVHWAVPELPSPPTASGLHWQRDPRYGGGATDASAVFVPDISSPENPFRCFAAAAAGDHTMKRRRRC

>LOC_Os02g07930_OsBBX2

MEVGNGKCGGGGAGCELCGGVAAVHCAADSAFLCLVCDDKVHGANFLASRHRRRRLGVEVVDEEDDARSTASSSCVSTADSASSTAAAAAAVESEDVRRRGRRGRRAPRAEAVLEGWAKRMGLSSGAARRRAAAAGAALRAVGRGVAASRVPIRVAMAAALWSEVASSSSRRRRRPGAGQAALLRRLEASAHVPARLLLTVASWMARASTPPAAEEGWAECS

>LOC_Os02g08150_OsBBX3

GACAVAPAAVHCRTCDGDGGGGGYLCAGCDAEHGRAGHERVWVCEVCELAPAAVTCKADAAALCAACDSDIHDANPLARRHERVPVHPIGSSAAPPPDALLLGGENDAAAAVDGGGGGKEVKLDFLFADFMDPYLGGSPELARFPHADSVVPNHNGSAGPAMELGFAGGGGAAVKPSYSSYTAASLGNSGSSSEVGLVPDAICGGGGGGIIELDFAQSKAAYLPYASTPSHSMSSSMDMGVAAPEMSDCAAAAAGRAYAAEGRAARLMRYREKRKNRRFEKTIRYASRKAYAETRPRVKGRFAKRADDHDAAAPPPQIMLDFAGYGVVPTF

>LOC_Os02g39360_OsBBX4

MKIQCDACESAAAAVVCCADEAALCAACDVEVHAANKLAGKHQRLPLEALSARLPRCDVCQEKAAFIFCVEDRALFCRDCDEPIHVPGTLSGNHQRYLATGIRVGFASASPCDGGSDAHDSDHHAPPMGSSEHHHHHQQPAPTVAVDTPSPQFLPQGWAVDELLQFSDYETGDKLQKESSPPLGFQELEWFADIDLFHNQAPKGGAAAGRTTAEVPELFASQAANDVAYYRPPTRTAAAAFTAATGFRQSKKARVELPDDEEDYLIVPDLG

>LOC_Os02g39710_OsBBX5

MEAVEDKAMVGVGGAVAAGYSSSSWGLGTRACDSCGGEAARLYCRADGAFLCARCDARAHGAGSRHARVWLCEVCEHAPAAVTCRADAAALCAACDADIHSANPLARRHERLPVAPFFGPLADAPQPFPFSQAAADAAAAREEDADDDRSNEAEAASWLLPEPDDNSHEDSAAAADAFFADTGAYLGVDLDFARSMDGIKAIGVPVAPPELDLTAGSLFYPEHSMAHSLSSSEVAIVPDALSAGSAAPPMVVVVASKGKEREARLMRYREKRKNRRFDKTIRYASRKAYAETRPRIKGRFAKRTADADDDDEAPCSPAFSALAASDGVVPSF

>LOC_Os02g43170_OsBBX6

MKVQCDVCAAEAASVFCCADEAALCDACDHRVHRANKLAGKHRRFSLLNPSASGRSPTSTTAPLCDICQEKRGFLFCKEDRAILCRECDVPVHTASELTMRHSRYLLTGVRLSSEPAASPAPPSEEENSSSFCCSADDAVPAPAAPATSHGGSSGSSSISEYLTTLPGWHVEDFLVDDATAEAAAAAAATSSGISANGPCQGVTRIGGLQESAGYPAWMAQQQLCCDGLVAGDASPASRERWVPQMYADQLAAGSKRSRTSTASSYSYW

>LOC_Os02g49230_OsBBX7

MDALCDFCREQRSMVYCRSDAASLCLSCDRNVHSANALSRRHTRTLLCDRCVGQPAAVRCLEENTSLCQNCDWNGHGAASSAAGHKRQTINCYSGCPSSAELSRIWSFSMDIPTVAAEPNCEEGINMMSINDNDVNNHCGAPEDGRLLDIASTALMSDLPTGDKFKPLIGSSSGDGMNLLPLNSDQPAEPVSTTPKAPCVTDKDMFNDGSVYGDFCVDDADLTFENYEELFGTSHVQTEQLFDDAGIDSYFEMKDVPADESNEQPKPVQPECSNVASVDSGMSNPAARADSSHCIPGRQAISNISLSFSGLTGESSAGYFQDCGVSSMILMGEPPWHPPGPESSSAGGSRDNALTRYKEKKKRRKFDKKIRYASRKARADVRKRVKGRFVKAGEAYDYDPLSQTRSY

>LOC_Os02g49880_OsBBX8

MSCSSEKAAGAVGGKAARACDSCLRRRARWYCAADDAFLCQGCDTSVHSANPLARRHERLRLRVSSPPPLTARASVEEEAAAAVGTTTTTTSKREGGVTPAWSKRKARTRRPQVKSVGQLLSRRLVVPEMAVESSDERKADEDGAHEELEGQLLYRVPVFDPSLAEFCSPPPIDDAAAASSSCFKEDAADGAVEDAKYPAAAASSPVQQLPDSFVNFEPTDAELREFAADMEALLGQGLDDSNELQDSFYMETLGLITPPVEESGRVKMELDGGVASNSRVSLPSCRAHPKPEDVESADVLDIDFNCTSPDEQKSSASNGAAADSQFFHRSLDLRLNYEAIIESWGNSPWTDGRPPHGQLDDFWPNDHHYSGLWAAGGGGHGAEVGMMTVRPRMDGPGREARVTRYREKRRTRLFSKKIRYEVRKLNAEKRPRMKGRFVKRPSAAAAPCAVT

>LOC_Os03g22770_OsBBX9

MGQDEVEVGAEKKDQELPEVEVVEEEEEEGSKKAAAGCDYCGDAAAVVYCRADAARLCLPCDRHVHGANGVCSRHARAPLCAACAAAGAVFRRGAGGFLCSNCDFSRHRHGGERDPAAPLHDRSTVHPYTGCPSALDLAALLGISYSDKAAAATAAAGGDDGGWWAIWEEPQVLSLEDLIVPTTSCHGFEPLLTPSSPKIQNSPDGKVNEEVIRQLTELANSDGGGAQIWAHREAAQAGDHQLPSWGTTTQHNTGHGNFGTANSNEVATMPTPGYENGGWDNSDYPALNDPCKVEFTYEQPPASSAEACISSFVQMSELCPSMSNGSSMEETHQTNPGNGTPMQVLPKMPEFVPCPDRNLVISRYKEKRKTRRFDRQVRYESRKARADSRLRIKGRFAKVNQI

>LOC_Os03g50310_OsBBX10

MASAAAATGAALGARTARACDGCMRRRARWHCPADDAFLCQACDASVHSANPLARRHHRVRLPSASSSPASSPRSAAAPRAGSDDPDAPAWLHGLKRRPRTPRTKPGGGGKHDASAATVAAAAASAVPDLEAEESGIVGDTDHDVGEEDDEDLLYRVPVFDPMLAELYNPVAADDEEQQIEQKPAARVVPFSEPSPEFASGSVEADGLSGFDVPDMELASFAADMESLLMGVDEGFDDLGFLDDEKPHVKLDLDMDMDFASISPAPAPEREERKRKRPEMILKLDYEGVIDSWARDGASPWFHGERPRFDPSESWPDFPAGSRGGLGAAVTAVTGGEREARVSRYREKRRTRLFAKKIRYEVRKLNAEKRPRMKGRFVKRAAALPPLPLPRHQHPPPPPPRALPPVPMMLAPRGAHGRYRF

>LOC_Os04g41560_OsBBX11

RALFCRDCDEPIHVPGTLSGNHQRYLTTGIRVGFSSVCSANADHLPPPAPKGNSKPPASGIAAAAAPKPAVSAAAQEVPSSPFLPPSGWAVEDLLQLSDYESSDKKGSPIGFKDLEWLDDIDLFHVQSPAKGGSTAAEVPELFASPQPASNMGLYKASGARQSKKPRVEIPDDDEDFFIVPDLG

>LOC_Os04g42020_OsBBX12

MEGDDKSAVVGGAYWGLAARACDACGGEAARLFCRADAAFLCAGCDARAHGPGSRHARVWLCEVCEHAPAAVTCRADAAALCAACDADIHSANPLARRHERLPVAPFFGALADAPKPGSGAHGGDAAAADDDGSNDAEAASWLLPEPDHGQKDGAVGATDELYADSDPYLDLDFARSMDDIKAIGVQNGPPELDITGGKLFYSDHSMNHSVSSSEAAVVPDAAAGGGAPMPVVSRGREREARLMRYREKRKSRRFEKTIRYASRKAYAETRPRIKGRFAKRTKGGAGADADADADADGEDEEMYSSAAAAVAALMAPGGSDADYGVDGVVPTF

>LOC_Os04g45690_OsBBX13

MKVQCDVCAAEAASVFCCADEAALCDACDRRVHSANKLAGKHRRFSLLQPLASSSSAQKPPLCDICQEKRGFLFCKEDRAILCRECDVTVHTTSELTRRHGRFLLTGVRLSSAPMDSPAPSEEEEEEAGEDYSCSPSSVAGTAAGSASDGSSISEYLTKTLPGWHVEDFLVDEATAASSSSDGLFQGGLLAQIGGVPDGYAAWAGREQLHSGVAVAADERASRERWVPQMNAEWGAGSKRPRASPPCLYW

>LOC_Os05g11510_OsBBX14

MSPPPPPYYHHLLLLRSSPTTTGGGARVLAAAELARMKLLCSACEAAEASVLCCADEAALCARCDRDIHAANRLAGKHLRLPLLSPASSSSSSAAALAPPPPSPPKCDICQESHAYFFCLEDRALLCRSCDVAVHTANAFVSAHRRFLLTGVQVGQEQDEHSPDPPEPSPPPPPPPPASKSDHPAPLYGEGGGGFSWDAADSPAAGGLPDWSAVVDQFGSPPPPRHTDTATVTTPPPTKRSPRAPAFGGQGGMMDWPLGEFFGGFTDFTGGFGFGFGDSGTSKADSGKLGGSTDGSPYYRSSSEDDRNADELFGQVPEIQWSVPELPSPPTASGLHWQRHPAATHGGGGGGPDTTAFVPDICSPDSCFPATTSKRRRQ

>LOC_Os06g01340_OsBBX15

MENEVGCECQLCGGRRGVVFCGAHGGRLCLQCDRALHQAHGGAGDHPRAPLCDSCNAAAAELRLNDGATLCGPCAYPYAYAYPYTYTYVYTGCPTPLEMMRLLHAAPPPPPATCSLQQRGEGEELLPTLLSATATPNTATAAPMAMPPPPLQHHTTTSLIMMIRNIHKREERNRAKLRYN

>LOC_Os06g05890_OsBBX16

MKIQCNACGAAEARVLCCADEAALCTACDEEVHAANKLAGKHQRVPLLSDDGGAAPAAAAPAVPKCDICQEASGYFFCLEDRALLCRDCDVSIHTVNSFVSVHQRFLLTGVQVGLDPADPVPPVADKHVKSAGGSVDSATKHLQRNPTDLSGENSASLPSQNVINGNYSRQSSVTMAKTGQVNWTMSNNTIRSIDPPPKYSSEESPALLLASHTSTMAAYSSQISKDSDRIYNLPFTGGNGSDSLHDWHVDEFFSNSEFGFAEHGSSKGDNAKPGSAGGSPQCRLAEGLFVEGLLGQVPDNPWTVPEVPSPPTASGLYWQNNLLCPSYDSTMFVPEISSLENSQNNFTVSAGLKRRRRQF

>LOC_Os06g15330_OsBBX17

MSSTAKAAAAGAVGAKSARACDGCLRRRARWYCAADDAFLCQGCDTSVHSANPLARRHERLRLRPSSPPPLVPPSGSGRRDEAVPAAWFKRKARTPRSHAAKSAAAFGQLLSRRLVVVPEAAAGSGGDSPEERKDEGEIVEEQEQLLYRVPIFDPALSEFCSPPPLEDAAAAVSCCNEDGAVENPTKPSMTTTTATTPPLQFFPDGQANFGPTDAELREFAADMEALLGRGLDDGNDEDSFCMETLGLIEPVDDDAGRVKVEADGDAGMTLAWCHELDTETSSGEMLDIDFDCGSPQAATTPDEKVGSSGPAAADDDAQLQQSNLALSLNYEAIIESWGTSPWTDGERPHVKLDDSWPRDYSVRATPCTPYASSHRILHNLAGTDDLLRRRAAVQGVWMAAAGVFGHGGEEQALTPRLGMDGGREARVSRYREKRRTRLFSKKIRYEVRKLNAEKRPRMKGRFVKRAAAAATAAVATACVA

>LOC_Os06g16370_OsBBX18

MNYNFGGNVFDQEVGVGGEGGGGGEGSGCPWARPCDGCRAAPSVVYCRADAAYLCASCDARVHAANRVASRHERVRVCEACERAPAALACRADAAALCVACDVQVHSANPLPAITIPATSVLAEAVVATATVLGDKDEEVDSWLLLSKDSDNNNNNNNNNDNDNNDNNNSNSSNNGMYFGEVDEYFDLVGYNSYYDNRIENNQDRQYGMHEQQEQQQQQQEMQKEFAEKEGSECVVPSQITMLSEQQHSGYGVVGADQAASMTAGVSAYTDSISNSISFSSMEAGIVPDSTVIDMPNSRILTPAGAINLFSGPSLQMSLHFSSMDREARVLRYREKKKARKFEKTIRYETRKAYAEARPRIKGRFAKRSDVQIEVDQMFSTAALSDGSYGTVPWF

>LOC_Os06g19444_OsBBX19

MGALCDFCGEQRSMVYCRSDAASLCLSCDRNVHSANALSRRHTRTLLCDRCASQPAMVRCLVENASLCQNCDWNGHSAGSSAAGHKRQTINCYSGCPSSSELSKIWTFVSDIPNVAPEPNCEQGISMMSISDSGVSNQDNAAGDSSLLDIASATLMSDLGTAGKPKSLIGSSSEAGVNLLPLATDQMAGSVDSTSAKVPYTADQDMFSKDSIYEDFCVDDVDLSFENYEELFGTSHIQTEQLFDDAGIDSYFESKEIPSGNSDEQPKLMQPVTSNAVSADSGMSIPGAKGDSSLCIPVRQARSSISLSFSGLTGESSAGDYQDCGVSPVLLMGEPPWHPPGPEGSFAGATRDDAITRYKEKKKRRKFDKKIRYASRKARADVRKRVKGRFVKAGEAYDYDPLCETRSY

>LOC_Os06g44450_OsBBX20

MGEGREARLMRYREKRKNRRFEKTIRYASRKAYAETRPRIKGRFAKRADHDADDADADADDPAAVPSSYMLDFGYGVVPSF

>LOC_Os06g45040_OsBBX21

MGGEAERCALCGAAAAVHCEADAAFLCAACDAKVHGANFLASRHHRRRVAAGAVVVVEVEEEEGYESGASAASSTSCVSTADSDVAASAAARRGRRRRPRAAARPRAEVVLEGWGKRMGLAAGAARRRAAAAGRALRACGGDVAAARVPLRVAMAAALWWEVAAHRVSGVSGAGHADALRRLEACAHVPARLLTAVASSMARARARRRAAADNEEGWDECSCSEAPNALGGPHVSDTARQK

>LOC_Os06g49880_OsBBX22

MRVQCDVCAAEPAAVLCCADEAALCSACDRRVHRANRLASKHRRLPLVHPSSSSSGDGGAAAAPLCDVCREKRGLVFCVEDRAILCADCDEPIHSANDLTAKHTRFLLVGAKLSPAALAEQPLPSSDCSSDDDAAAAATEEEYHSSAASTGAAVSAPLDASSNGAGGGGGVGGSSISDYLTTICPGWRVEDLLPDDDAFAAAAAQAGKEKDERVPFLDADLFDVVAGRPEKKGGAWAPHVPHLPAWCLDEVPVVVAASAAPAATPVKAKQGHVRDSHWSDSDAFAVPEFSPPPPPAKRARPSSQFWCF

>LOC_Os07g47140_OsBBX23

MARDDDPAKKLAVDGGVAAAARCCDFCGGLPAVVYCRADSARLCLPCDRHVHAANTVSTRHARAPLCSACRAAPAAAFHRGDGFLCSSCDFDERLRRGSIGGGGDELPLDDRAAVEGYTGCPSIGELAAILGVVGGDSDKPADDGWWSASWEEEAPQVLSLDDIIVPTTSCHGLRPLLTPPSPENQSSPDNGELDGEVVRQLGELARSEAAAQATFVAGDQLASWASPEFTSGHGDFGIEAASTTVPSCENETWIMSTDCTDPTDASKTDIAREEAPASSSAEPCLSSLVEISEICRSMSYSGSGIDNGGHDPSTLAIMPTQALPKKGVYDIAYPDRGTVISRYKEKRKNRRFDKQIRYESRKARADGRLRIKGRFAKSN

>LOC_Os08g08120_OsBBX24

MSVAAEGKEKGVGGGGGGAGAGACELCGAAARVYCGADEATLCWGCDAQVHGANFLVARHARALLCRGCARPTPWRAAGPRLGPTASLCERCVRRGGGGRGGGGGGGAAGGGGRGGGGDEEMGGEGDEEEEDEDEEVVVEEEEDEDDEDEEGEGEGENQVVPWAEEAEATPPPVASSTSSSSREAAANGANAADRVKEDQPCSTSQPSLCRYASSAHHGGGGRSDEATSSRNGGGVGGRFLASRHRKRSPSDFRRSGLAQSVSGVQGRNCSNAVVGRNDFS

>LOC_Os08g15050_OsBBX25

MMASDGSASPASCGGAACGVCGGAATVYCAADAAALCVPCDAAVHAANPLASRHDRVPLAVAMAAASSGVYDHLFAPDDDAASSWAAAAAAGAAVQGQGQGSPNDSSSSFTNDSAAASSSSDDDSTAAAGVSGAGGAGAAATKEAKFWLSFSDDGRADGVGFYMDSTTAATAAYGVVPTF

>LOC_Os08g42440_OsBBX26

MKDGGGGGGRGQQQQWPCDYCGEAAAALHCRADAARLCVACDRHVHAANALSRKHVRAPLCAACAARPAAARVASASAPAFLCADCDTGCGGDDGAALRVPVEGFSGCPAAAELAASWGLDLPGGCGGEEEEADDAFFSALDYSMLAVDPVLRDLYVPCDPPEVVVAGGGRRLKGEALGHQLAEMARREAETAHPHTQPHSDLSPRTPRRTSAAASGRLQEKQAPPPLPHAAATAAPLPYTSLLMMAPANCTELMENNRVGDEDENVLWESTAPSVPPTQIWDFNLGKSRDHNENSALEVGFGSNNGGFMIKSYNDMLKEISSGTTKDLEDIYDSRYFAAAEDIMSTNVCQLSSKNPSTRSNKRKASSCASTIDGPTTSTSHVPAASGALGGSSNDRGSALPKEISFCDQTVVPTGADQRPCTIKIDSETLAQNRDSAMQRYREKKKNRRYEKHIRYESRKLRADTRKRVKGRFVKSNGAPDDVSNGG

>LOC_Os09g06464_OsBBX27

MLKLEPEFPGLPQRCDSCRSAPCAFYCLADSAALCATCDADVHSVNPLARRHRRVPMGVVAAPGAGGAFVVRPAGGVNSSWPIREGRRCDYDDDDADAAGEEDEEATSWLLFDPLKDSSDQGLPPFGDALVADFLNLGGGAGEKEDASSSKDCSSSHGKSSEGSHEFAVPGEPVPERQGFGAVSMDITDYDASNFRRGYSFGASLGHSVSMSSLENMSTVPDCGVPDITTSYLRSSKSTIDLFTAAAGSPVAAHSIMSPPQFMGAIDREARVHRYREKRKTRRFEKTIRYASRKAYAETRPRIKGRFAKRSDTDLEVDQYFSTTADSSCGVVPTF

>LOC_Os09g33550_OsBBX28

MTWRSCDYCGEAAAALHCRADAARLCVACDRHVHGANALSRRHVRAPLCARCEARPAAARVAAVAGAGGCGGGGEARFLCAGCADDDGAEAARVPVVGFSGCPGAAELAASWGLDLGGGGGRDEFEEDPFFPEAGYPMLAADRVLRDMYVPCDPPPEVAAGGRGRRLKGDSLCHQLAELARREMESAPAQANSGSISPSARRGSAAAIRHEAAAAAAAQRATLPYKSTPVTEAAGCGDVGNGEQFTDDNELVWQRTAPSDPPCQIWDFNLGKSRDHDEHSALELHFGPKDGGFMIKSYNDMIEEVSSSSRKDLQYIYDSTYSFATEDIVSANIYQLTPKQLSTATSGNRRHKNEQHGLTNDGPSSSRIDSKTIAMNRDNAMQRYREKRKTRRYDKHIRYESRKMRADTRTRVKGRFVRATDIFNVGGGDGG

>LOC_Os09g35880_OsBBX29

MRTICDVCESAPAVLFCVADEAALCRSCDEKVHMCNKLARRHVRVGLADPNKVQRCDICENAPAFFYCEIDGTSLCLSCDMTVHVGGKRTHGRYLLLRQRVEFPGDKPGHMDDVAMQQKDPENRTDQKKAPHSVTKEQMANHHNVSDDPASDGNCDDQGNIDSKMIDLNMRPVRTHGQGSNSQTQGVDVSVNNHDSPGVVPTCNFEREANK

>LOC_Os12g10660_OsBBX30

MKIGCDACEQAEAAVLCCADEAALCRRCDAAVHSANRLAGKHTRVALLLPSSSSAAAGDDDHHPTCDICQEKTGYFFCLEDRALLCRSCDVAVHTATAHAAAHRRFLITGVRIGGSVDAAAAADVIVSPTSSSIAPAGSASSNHAGAAGNNNGRSPAPVRFSGGDGGVEPEQQWPWSDVFAADDDDDVSAAMEQCYYHGISEPHSSSLTG

>AT5G15840_AtBBX1

MLKQESNDIGSGENNRARPCDTCRSNACTVYCHADSAYLCMSCDAQVHSANRVASRHKRVRVCESCERAPAAFLCEADDASLCTACDSEVHSANPLARRHQRVPILPISGNSFSSMTTTHHQSEKTMTDPEKRLVVDQEEGEEGDKDAKEVASWLFPNSDKNNNNQNNGLLFSDEYLNLVDYNSSMDYKFTGEYSQHQQNCSVPQTSYGGDRVVPLKLEESRGHQCHNQQNFQFNIKYGSSGTHYNDNGSINHNAYISSMETGVVPESTACVTTASHPRTPKGTVEQQPDPASQMITVTQLSPMDREARVLRYREKRKTRKFEKTIRYASRKAYAEIRPRVNGRFAKREIEAEEQGFNTMLMYNTGYGIVPSF

>AT5G15850_AtBBX2

MLKVESNWAQACDTCRSAACTVYCRADSAYLCSSCDAQVHAANRLASRHERVRVCQSCERAPAAFFCKADAASLCTTCDSEIHSANPLARRHQRVPILPISEYSYSSTATNHSCETTVTDPENRLVLGQEEEDEDEAEAASWLLPNSGKNSGNNNGFSIGDEFLNLVDYSSSDKQFTDQSNQYQLDCNVPQRSYGEDGVVPLQIEVSKGMYQEQQNFQLSINCGSWGALRSSNGSLSHMVNVSSMDLGVVPESTTSDATVSNPRSPKAVTDQPPYPPAQMLSPRDREARVLRYREKKKMRKFEKTIRYASRKAYAEKRPRIKGRFAKKKDVDEEANQAFSTMITFDTGYGIVPSF

>AT3G02380_AtBBX3

MLKEESNESGTWARACDTCRSAACTVYCEADSAYLCTTCDARVHAANRVASRHERVRVCQSCESAPAAFLCKADAASLCTACDAEIHSANPLARRHQRVPILPLSANSCSSMAPSETDADNDEDDREVASWLLPNPGKNIGNQNNGFLFGVEYLDLVDYSSSMDNQFEDNQYTHYQRSFGGDGVVPLQVEESTSHLQQSQQNFQLGINYGFSSGAHYNNNSLKDLNHSASVSSMDISVVPESTASDITVQHPRTTKETIDQLSGPPTQVVQQLTPMEREARVLRYREKKKTRKFDKTIRYASRKAYAEIRPRIKGRFAKRIETEAEAEEIFSTSLMSETGYGIVPSF

>AT2G24790_AtBBX4

MASSSRLCDSCKSTAATLFCRADAAFLCGDCDGKIHTANKLASRHERVWLCEVCEQAPAHVTCKADAAALCVTCDRDIHSANPLSRRHERVPITPFYDAVGPAKSASSSVNFVDEDGGDVTASWLLAKEGIEITNLFSDLDYPKIEVTSEENSSGNDGVVPVQNKLFLNEDYFNFDLSASKISQQGFNFINQTVSTRTIDVPLVPESGGVTAEMTNTETPAVQLSPAEREARVLRYREKRKNRKFEKTIRYASRKAYAEMRPRIKGRFAKRTDSRENDGGDVGVYGGFGVVPSF

>AT5G24930_AtBBX5

MDPTWIDSLTRSCEANSNTNHKRKRERETLKHREKKKKRFRERKMASKLCDSCKSATAALYCRPDAAFLCLSCDSKVHAANKLASRHARVWMCEVCEQAPAHVTCKADAAALCVTCDRDIHSANPLARRHERVPVTPFYDSVSSDGSVKHTAVNFLDDCYFSDIDGNGSREEEEEEAASWLLLPNPKTTTTATAGIVAVTSAEEVPGDSPEMNTGQQYLFSDPDPYLDLDYGNVDPKVESLEQNSSGTDGVVPVENRTVRIPTVNENCFEMDFTGGSKGFTYGGGYNCISHSVSSSSMEVGVVPDGGSVADVSYPYGGPATSGADPGTQRAVPLTSAEREARVMRYREKRKNRKFEKTIRYASRKAYAEMRPRIKGRFAKRTDTNESNDVVGHGGIFSGFGLVPTF

>AT5G57660_AtBBX6

MGFGLESIKSISGGWGAAARSCDACKSVTAAVFCRVDSAFLCIACDTRIHSFTRHERVWVCEVCEQAPAAVTCKADAAALCVSCDADIHSANPLASRHERVPVETFFDSAETAVAKISASSTFGILGSSTTVDLTAVPVMADDLGLCPWLLPNDFNEPAKIEIGTENMKGSSDFMFSDFDRLIDFEFPNSFNHHQNNAGGDSLVPVQTKTEPLPLTNNDHCFDIDFCRSKLSAFTYPSQSVSHSVSTSSIEYGVVPDGNTNNSVNRSTITSSTTGGDHQASSMDREARVLRYREKRKNRKFEKTIRYASRKAYAESRPRIKGRFAKRTETENDDIFLSHVYASAAHAQYGVVPTF

>AT3G07650_AtBBX7

MGYMCDFCGEQRSMVYCRSDAACLCLSCDRSVHSANALSKRHSRTLVCERCNAQPATVRCVEERVSLCQNCDWSGHNNSNNNNSSSSSTSPQQHKRQTISCYSGCPSSSELASIWSFCLDLAGQSICEQELGMMNIDDDGPTDKKTCNEDKKDVLVGSSSIPETSSVPQGKSSSAKDVGMCEDDFYGNLGMDEVDMALENYEELFGTAFNPSEELFGHGGIDSLFHKHQTAPEGGNSVQPAGSNDSFMSSKTEPIICFASKPAHSNISFSGVTGESSAGDFQECGASSSIQLSGEPPWYPPTLQDNNACSHSVTRNNAVMRYKEKKKARKFDKRVRYASRKARADVRRRVKGRFVKAGEAYDYDPLTPTRSY

>AT5G48250_AtBBX8

MGYMCDFCGEQRSMVYCRSDAACLCLSCDRNVHSANALSKRHSRTLVCERCNAQPASVRCSDERVSLCQNCDWSGHDGKNSTTTSHHKRQTINCYSGCPSSAELSSIWSFCMDLNISSAEESACEQGMGLMTIDEDGTGEKSGVQKINVEQPETSSAAQGMDHSSVPENSSMAKELGVCEDDFNGNLISDEVDLALENYEELFGSAFNSSRYLFEHGGIGSLFEKDEAHEGSMQQPALSNNASADSFMTCRTEPIICYSSKPAHSNISFSGITGESNAGDFQDCGASSMKQLSREPQPWCHPTAQDIIASSHATTRNNAVMRYKEKKKARKFDKRVRYVSRKERADVRRRVKGRFVKSGEAYDYDPMSPTRSY

>AT4G15250_AtBBX9

MEARCDFCGTEKALIYCKSDSAKLCLNCDVNVHSANPLSQRHTRSLLCEKCSLQPTAVHCMNENVSLCQGCQWTASNCTGLGHRLQSLNPYSDCPSPSDFGKIWSSTLEPSVTSLVSPFSDTLLQELDDWNGSSTSVVTQTQNLKDYSSFFPMESNLPKVIEEECSGLDLCEGINLDDAPLNFNASNDIIGCSSLDNTKCYEYEDSFKEENNIGLPSLLLPTLSGNVVPNMSLSMSNLTGESNATDYQDCGISPGFLIGDSPWESNVEVSFNPKLRDEAKKRYKQKKSKRMFGKQIRYASRKARADTRKRVKGRFVKSGETFEYDPSLVM

>AT3G21880_AtBBX10

MEPKCDHCATSQALIYCKSDLAKLCLNCDVHVHSANPLSHRHIRSLICEKCFSQPAAIRCLDEKVSYCQGCHWHESNCSELGHRVQSLNPFSGCPSPTDFNRMWSSILEPPVSGLLSPFVGSFPLNDLNNTMFDTAYSMVPHNISYTQNFSDNLSFFSTESKGYPDMVLKLEEGEEDLCEGLNLDDAPLNFDVGDDIIGCSSEVHIEPDHTVPNCLLIDKTNTSSFTGSNFTVDKALEASPPGQQMNINTGLQLPLSPVLFGQIHPSLNITGENNAADYQDCGMSPGFIMSEAPWETNFEVSCPQARNEAKLRYKEKKLKRSFGKQIRYASRKARADTRKRVKGRFVKAGDSYDYDPSSPTTNN

>AT2G47890_AtBBX11

MEAEEGHQRDRLCDYCDSSVALVYCKADSAKLCLACDKQVHVANQLFAKHFRSLLCDSCNESPSSLFCETERSVLCQNCDWQHHTASSSLHSRRPFEGFTGCPSVPELLAIVGLDDLTLDSGLLWESPEIVSLNDLIVSGGSGTHNFRATDVPPLPKNRHATCGKYKDEMIRQLRGLSRSEPGCLKFETPDAEIDAGFQFLAPDLFSTCELESGLKWFDQQDHEDFPYCSLLKNLSESDEKPENVDRESSVMVPVSGCLNRCEEETVMVPVITSTRSMTHEINSLERNSALSRYKEKKKSRRYEKHIRYESRKVRAESRTRIRGRFAKAADP

>AT2G33500_AtBBX12

MGTSTTESVVACEFCGERTAVLFCRADTAKLCLPCDQHVHSANLLSRKHVRSQICDNCSKEPVSVRCFTDNLVLCQECDWDVHGSCSSSATHERSAVEGFSGCPSVLELAAVWGIDLKGKKKEDDEDELTKNFGMGLDSWGSGSNIVQELIVPYDVSCKKQSFSFGRSKQVVFEQLELLKRGFVEGEGEIMVPEGINGGGSISQPSPTTSFTSLLMSQSLCGNGMQWNATNHSTGQNTQIWDFNLGQSRNPDEPSPVETKGSTFTFNNVTHLKNDTRTTNMNAFKESYQQEDSVHSTSTKGQETSKSNNIPAAIHSHKSSNDSCGLHCTEHIAITSNRATRLVAVTNADLEQMAQNRDNAMQRYKEKKKTRRYDKTIRYETRKARAETRLRVKGRFVKATDP>AT1G28050_AtBBX13

MSSSERVPCDFCGERTAVLFCRADTAKLCLPCDQQVHTANLLSRKHVRSQICDNCGNEPVSVRCFTDNLILCQECDWDVHGSCSVSDAHVRSAVEGFSGCPSALELAALWGLDLEQGRKDEENQVPMMAMMMDNFGMQLDSWVLGSNELIVPSDTTFKKRGSCGSSCGRYKQVLCKQLEELLKSGVVGGDGDDGDRDRDCDREGACDGDGDGEAGEGLMVPEMSERLKWSRDVEEINGGGGGGVNQQWNATTTNPSGGQSSQIWDFNLGQSRGPEDTSRVEAAYVGKGAASSFTINNFVDHMNETCSTNVKGVKEIKKDDYKRSTSGQVQPTKSESNNRPITFGSEKGSNSSSDLHFTEHIAGTSCKTTRLVATKADLERLAQNRGDAMQRYKEKRKTRRYDKTIRYESRKARADTRLRVRGRFVKASEAPYP

>AT1G68520_AtBBX14

MMKSLASAVGGKTARACDSCVKRRARWYCAADDAFLCHACDGSVHSANPLARRHERVRLKSASAGKYRHASPPHQATWHQGFTRKARTPRGGKKSHTMVFHDLVPEMSTEDQAESYEVEEQLIFEVPVMNSMVEEQCFNQSLEKQNEFPMMPLSFKSSDEEDDDNAESCLNGLFPTDMELAQFTADVETLLGGGDREFHSIEELGLGEMLKIEKEEVEEEGVVTREVHDQDEGDETSPFEISFDYEYTHKTTFDEGEEDEKEDVMKNVMEMGVNEMSGGIKEEKKEKALMLRLDYESVISTWGGQGIPWTARVPSEIDLDMVCFPTHTMGESGAEAHHHNHFRGLGLHLGDAGDGGREARVSRYREKRRTRLFSKKIRYEVRKLNAEKRPRMKGRFVKRSSIGVAH

>AT1G25440_AtBBX15

MMKSLANAVGAKTARACDSCVKRRARWYCAADDAFLCQSCDSLVHSANPLARRHERVRLKTASPAVVKHSNHSSASPPHEVATWHHGFTRKARTPRGSGKKNNSSIFHDLVPDISIEDQTDNYELEEQLICQVPVLDPLVSEQFLNDVVEPKIEFPMIRSGLMIEEEEDNAESCLNGFFPTDMELEEFAADVETLLGRGLDTESYAMEELGLSNSEMFKIEKDEIEEEVEEIKAMSMDIFDDDRKDVDGTVPFELSFDYESSHKTSEEEVMKNVESSGECVVKVKEEEHKNVLMLRLNYDSVISTWGGQGPPWSSGEPPERDMDISGWPAFSMVENGGESTHQKQYVGGCLPSSGFGDGGREARVSRYREKRRTRLFSKKIRYEVRKLNAEKRPRMKGRFVKRASLAAAASPLGVNY

>AT1G73870_AtBBX16

MVVDVESRTASVTGEKMAARGCDACMKRSRASWYCPADDAFLCQSCDASIHSANHLAKRHERVRLQSSSPTETADKTTSVWYEGFRRKARTPRSKSCAFEKLLQIESNDPLVPELGGDEDDGFFSFSSVEETEESLNCCVPVFDPFSDMLIDDINGFCLVPDEVNNTTTNGELGEVEKAIMDDEGFMGFVPLDMDLEDLTMDVESLLEEEQLCLGFKEPNDVGVIKEENKVGFEINCKDLKRVKDEDEEEEEAKCENGGSKDSDREASNDKDRKTSLFLRLDYGAVISAWDNHGSPWKTGIKPECMLGGNTCLPHVVGGYEKLMSSDGSVTRQQGRDGGGSDGEREARVLRYKEKRRTRLFSKKIRYEVRKLNAEQRPRIKGRFVKRTSLLT

>AT1G49130_AtBBX17

MTSHQNIKISEKIMISKYQEDVKQPRACELCLNKHAVWYCASDDAFLCHVCDESVHSANHVATKHERVCLRTNEISNDVRGGTTLTSVWHSGFRRKARTPRSRYEKKPQQKIDDERRREDPRVPEIGGEVMFFIPEANDDDMTSLVPEFEGFTEMGFFLSNHNGTEETTKQFNFEEEADTMEDLYYNGEEEDKTDGAEACPGQYLMSCKKDYDNVITVSEKTEEIEDCYENNARHRLNYENVIAAWDKQESPRDVKNNTSSFQLVPPGIEEKRVRSEREARVWRYRDKRKNRLFEKKIRYEVRKVNADKRPRMKGRFVRRSLAIDS

>AT2G21320_AtBBX18

MRILCDACESAAAIVFCAADEAALCCSCDEKVHKCNKLASRHLRVGLADPSNAPSCDICENAPAFFYCEIDGSSLCLQCDMVVHVGGKRTHRRFLLLRQRIEFPGDKPNHADQLGLRCQKASSGRGQESNGNGDHDHNMIDLNSNPQRVHEPGSHNQEEGIDVNNANNHEHE

>AT4G38960_AtBBX19

MRILCDACENAAAIIFCAADEAALCRPCDEKALHMRLDISKCSESVKRVQIVETSSLIWWIKMGTFCLQSLHLVVHMCNKLASRHVRVGLAEPSNAPCCDICENAPAFFYCEIDGSSLCLQCDMVVHVGGKRTHGRFLLLRQRIEFPGDKPKENNTRDNLQNQRVSTNGNGEANGKIDDEMIDLNANPQRVHEPSSNNNGIDVNNENNHEPAGLVPVGPFKRESEK

>AT4G39070_AtBBX20

MKIWCAVCDKEEASVFCCADEAALCNGCDRHVHFANKLAGKHLRFSLTSPTFKDAPLCDICGERRALLFCQEDRAILCRECDIPIHQANEHTKKHNRFLLTGVKISASPSAYPRASNSNSAAAFGRAKTRPKSVSSEVPSSASNEVFTSSSSTTTSNCYYGIEENYHHVSDSGSGSGCTGSISEYLMETLPGWRVEDLLEHPSCVSYEDNIITNNNNSESYRVYDGSSQFHHQGFWDHKPFS

>AT1G75540_AtBBX21

MKIRCDVCDKEEASVFCTADEASLCGGCDHQVHHANKLASKHLRFSLLYPSSSNTSSPLCDICQDKKALLFCQQDRAILCKDCDSSIHAANEHTKKHDRFLLTGVKLSATSSVYKPTSKSSSSSSSNQDFSVPGSSISNPPPLKKPLSAPPQSNKIQPFSKINGGDASVNQWGSTSTISEYLMDTLPGWHVEDFLDSSLPTYGFSKSGDDDGVLPYMEPEDDNNTKRNNNNNNNNNNNTVSLPSKNLGIWVPQIPQTLPSSYPNQYFSQDNNIQFGMYNKETSPEVVSFAPIQNMKQQGQNNKRWYDDGGFTVPQITPPPLSSNKKFRSFW

>AT1G78600_AtBBX22

MKIQCNVCEAAEATVLCCADEAALCWACDEKIHAANKLAGKHQRVPLSASASSIPKCDICQEASGFFFCLQDRALLCRKCDVAIHTVNPHVSAHQRFLLTGIKVGLESIDTGPSTKSSPTNDDKTMETKPFVQSIPEPQKMAFDHHHHQQQQEQQEGVIPGTKVNDQTSTKLPLVSSGSTTGSIPQWQIEEIFGLTDFDQSYEYMENNGSSKTDVLKMKLLDSACLGKKLEKADTSRRGDSDSSSMMRSAEEDGEDNNNCLGGETSWAVPQIQSPPTASGLNWPKHFHHHSVFVPDITSSTPYTGSSPNQRVGKRRRRF

>AT4G10240_AtBBX23

MKIQCEVCEKAEAEVLCCSDEAVLCKPCDIKVHEANKLFQRHHRVALQKDAASATTASGAPLCDICQERKGYFFCLEDRAMLCNDCDEAIHTCNSHQRFLLSGVQVSDQSLTENSECSTSFSSETYQIQSKVSLNSQYSSEETEAGNSGEIVHKNPSVILSP

>AT1G06040_AtBBX24

MKIQCDVCEKAPATVICCADEAALCPQCDIEIHAANKLASKHQRLHLNSLSTKFPRCDICQEKAAFIFCVEDRALLCRDCDESIHVANSRSANHQRFLATGIKVALTSTICSKEIEKNQPEPSNNQQKANQIPAKSTSQQQQQPSSATPLPWAVDDFFHFSDIESTDKKGQLDLGAGELDWFSDMGFFGDQINDKALPAAEVPELSVSHLGHVHSYKPMKSNVSHKKPRFETRYDDDDEEHFIVPDLG

>AT2G31380_AtBBX25

MKIQCDVCEKAPATLICCADEAALCAKCDVEVHAANKLASKHQRLFLDSLSTKFPPCDICLEKAAFIFCVEDRALLCRDCDEATHAPNTRSANHQRFLATGIRVALSSTSCNQEVEKNHFDPSNQQSLSKPPTQQPAAPSPLWATDEFFSYSDLDCSNKEKEQLDLGELDWLAEMGLFGDQPDQEALPVAEVPELSFSHLAHAHSYNRPMKSNVPNKKQRLEYRYDDEEEHFLVPDLG

>AT1G60250_AtBBX26

MAQVCHTCRHVTAVIHCVTEALNFCLTCDNLRHHNNIHAEHVRYQLCDNCSMYPSILFCYEDGMVLCQSCYSHHYNCATNGHQTQVVFANMNNQHHDHAHMPHVVHHNNNNNHQQQHVGGHQRRAEMFERSCHGDNNCERWMFAMRCELCVASNSNAVVYCPTHNQILCDSCDRMIHSHEDAVPPHSRCKLCVICKRPSRRFLIGGYQFNFPPVHPPAAEGIPVTPPTELPQQDINYDYLDDVDDFSWFGR

>AT1G68190_AtBBX27

MLCIIIIENMERVCEFCKAYRAVVYCIADTANLCLTCDAKVHSANSLSGRHLRTVLCDSCKNQPCVVRCFDHKMFLCHGCNDKFHGGGSSEHRRRDLRCYTGCPPAKDFAVMWGFRVMDDDDDVSLEQSFRMVKPKVQREGGFILEQILELEKVQLREENGSSSLTERGDPSPLELPKKPEEQLIDLPQTGKELVVDFSHLSSSSTLGDSFWECKSPYNKNNQLWHQNIQDIGVCEDTICSDDDFQIPDIDLTFRNFEEQFGADPEPIADSNNVFFVSSLDKSHEMKTFSSSFNNPIFAP

>AT4G27310_AtBBX28

MGKKCDLCNGVARMYCESDQASLCWDCDGKVHGANFLVAKHTRCLLCSACQSLTPWKATGLRLGPTFSVCESCVALKNAGGGRGNRVLSENRGQEEVNSFESEEDRIREDHGDGDDAESYDDDEEEDEDEEYSDDEDEDDDEDGDDEEAENQVVPWSAAAQVPPVMSSSSSDGGSGGSVTKRTRARENSDLLCSDDEIGSSSAQGSNYSRPLKRSAFKSTVVV

>AT5G54470_AtBBX29

MGKKKCELCCGVARMYCESDQASLCWDCDGKVHGANFLVAKHMRCLLCSACQSHTPWKASGLNLGPTVSICESCLARKKNNNSSLAGRDQNLNQEEEIIGCNDGAESYDEESDEDEEEEEVENQVVPAAVEQELPVVSSSSSVSSGEGDQVVKRTRLDLDLNLSDEENQSRPLKRLSRDEGLSRSTVVMNSSIVKLHGGRRKAEGCDTSSSSSFY

>AT4G15248_AtBBX30

MCRGFEKEEERRSDNGGCQRLCTESHKAPVSCELCGENATVYCEADAAFLCRKCDRWVHSANFLARRHLRRVICTTCRKLTRRCLVGDNFNVVLPEIRMIARIEEHSSDHKIPFVFL

>AT3G21890_AtBBX31

MCRGLNNEESRRSDGGGCRSLCTRPSVPVRCELCDGDASVFCEADSAFLCRKCDRWVHGANFLAWRHVRRVLCTSCQKLTRRCLVGDHDFHVVLPSVTTVGETTVENRSEQDNHEVPFVFL

>AT3G21150_AtBBX32

MVSFCELCGAEADLHCAADSAFLCRSCDAKFHASNFLFARHFRRVICPNCKSLTQNFVSGPLLPWPPRTTCCSESSSSSCCSSLDCVSSSELSSTTRDVNRARGRENRVNAKAVAVTVADGIFVNWCGKLGLNRDLTNAVVSYASLALAVETRPRATKRVFLAAAFWFGVKNTTTWQNLKKVEDVTGVSAGMIRAVESKLARAMTQQLRRWRVDSEEGWAENDNV
