# Supplementary material for: Characterization and expression profiles of the B-box gene family during plant growth and under low-nitrogen stress in Saccharum
Source: BMC Genomics. 2023 Feb 17;24:79. doi: 10.1186/s12864-023-09185-9 (PMC9936747; doi:10.1186/s12864-023-09185-9)
Supplement: Supplementary file 8 — Additional file 8: Fig. S1. WebLogos of the amino acid sequences alignment of B-box1, B-box2, and CCT were shown. The y-axis and x-axis indicated the conservation rate of each amino acid and the conserved sequences of the domain, respectively. The height of each letter indicates how conserved the residue is across all proteins. Fig. S2. The tertiary structure modeling of SsBBX proteins. The structure image was generated using the SWISS-MODEL software. Fig. S3. Expression patterns of the sugarcane BBX genes from the sugarcane transcriptome data in five different conditions. (a) Expression patterns of the BBX genes in the roots of Saccharum hybrid cultivar YT55 under low-potassium stress, and samples were collected at 0, 6, 12, 24, 48, and 72 h (PRJNA262715). (b) Expression patterns of the BBX genes in the leaves of Saccharum hybrid cultivar GX87–16 under cold stress, and samples were collected at 0, 0.5, 1, and 6 h (PRJNA636260). (c) Expression patterns of the BBX genes in the leaves of Saccharum hybrid cultivar Co_06022 (susceptible cultivar) and Co_8021 (resistant cultivar) after 0, 2, 6, and 10 d drought stress and recovery treatment (PRJNA590595). (d) Expression patterns of the BBX genes in the buds of Saccharum hybrid cultivar ROC22 (susceptible cultivar) and YC05–179 (resistant cultivar) after smut pathogen infection at 0, 1, 2, and 5 d (PRJCA011580). (e) Expression patterns of the BBX genes in the buds of Saccharum hybrid cultivar ROC22 (susceptible cultivar) and GT42 (resistant cultivar) after ABA treatment at 0, 1, and 6 h (PRJNA555450). The heat map was plotted with the TBtools software (v1.098), with the transcript level of the BBX genes transformed as log2 FPKM (fragments per kilobase million), ranging from blue (low expression level) to red (high expression level). [file 12864_2023_9185_MOESM8_ESM.docx]

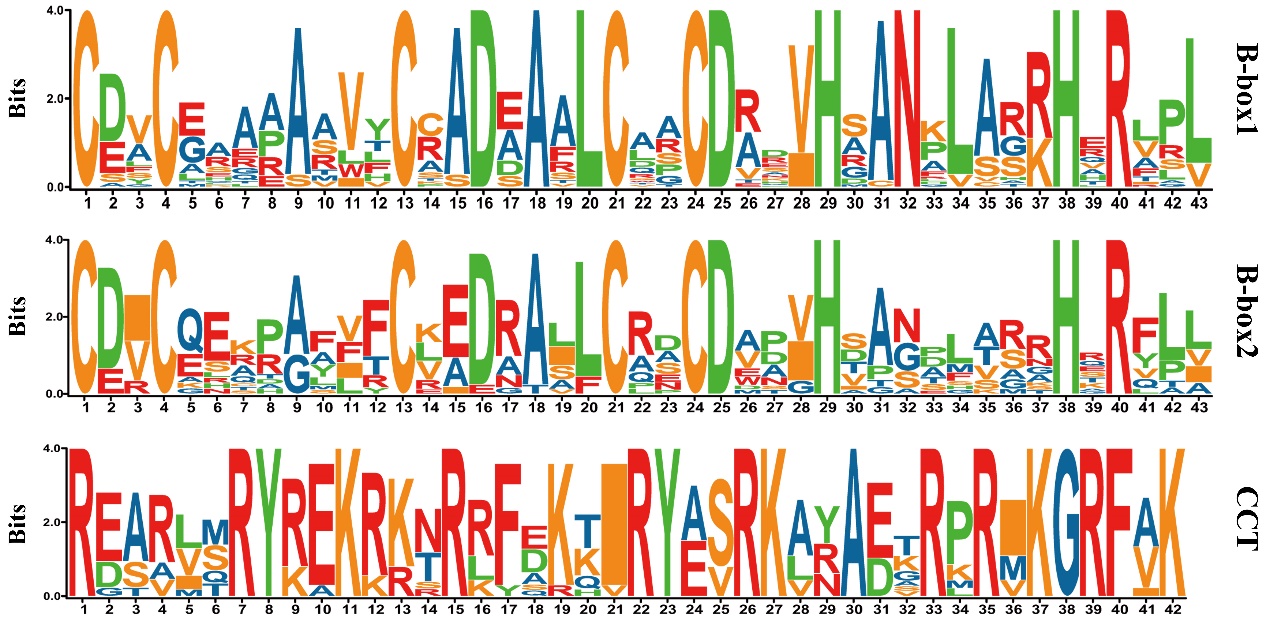


**Figure S1.** Web logos of the amino acid sequences alignment of B-box1, B-box2, and CCT were shown. The y-axis and x-axis indicated the conservation rate of each amino acid and the conserved sequences of the domain, respectively. The height of each letter indicates how conserved the residue is across all proteins.


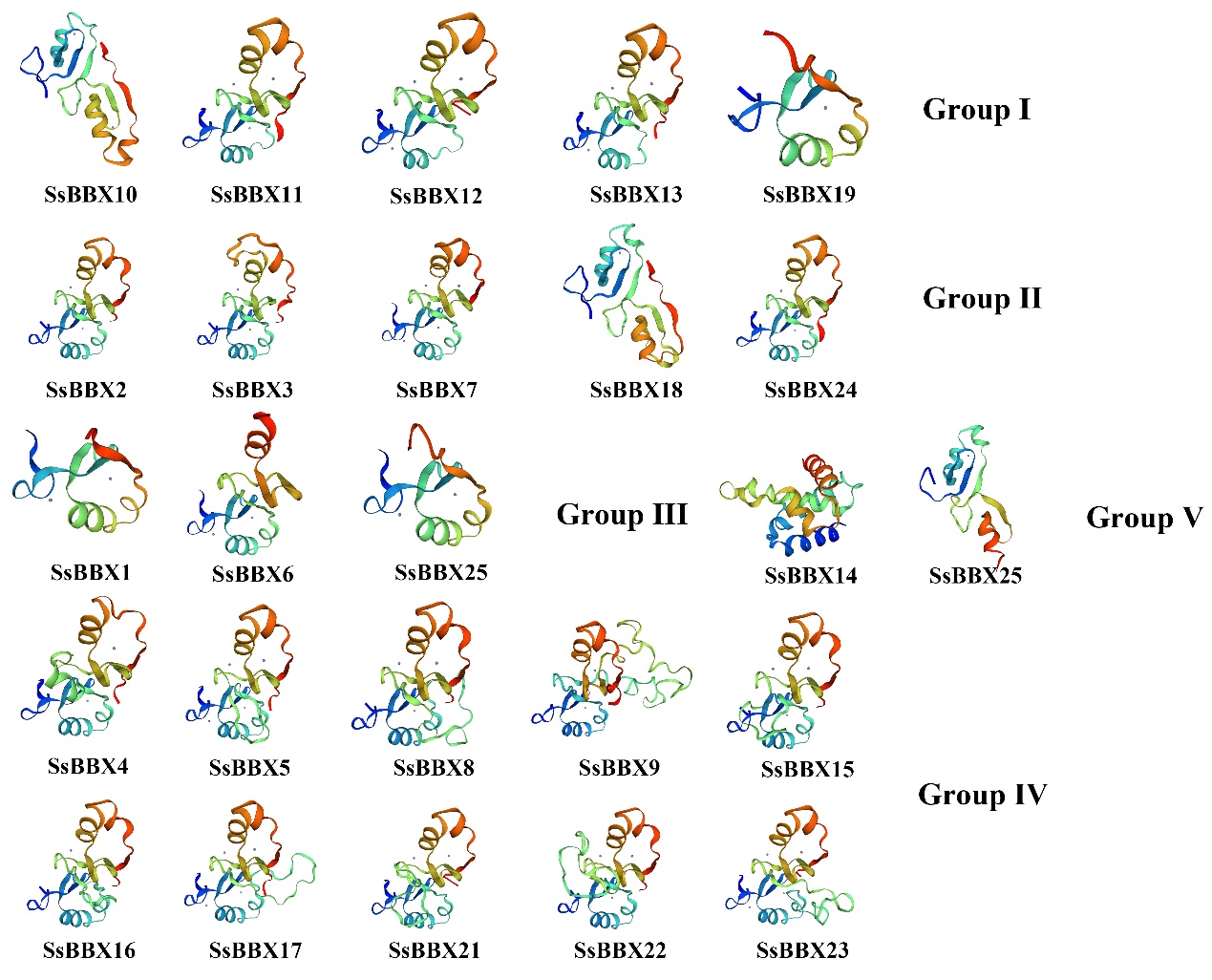


**Figure S2.** The tertiary structure modeling of SsBBX proteins. The structure image was generated using the SWISS-MODEL software.


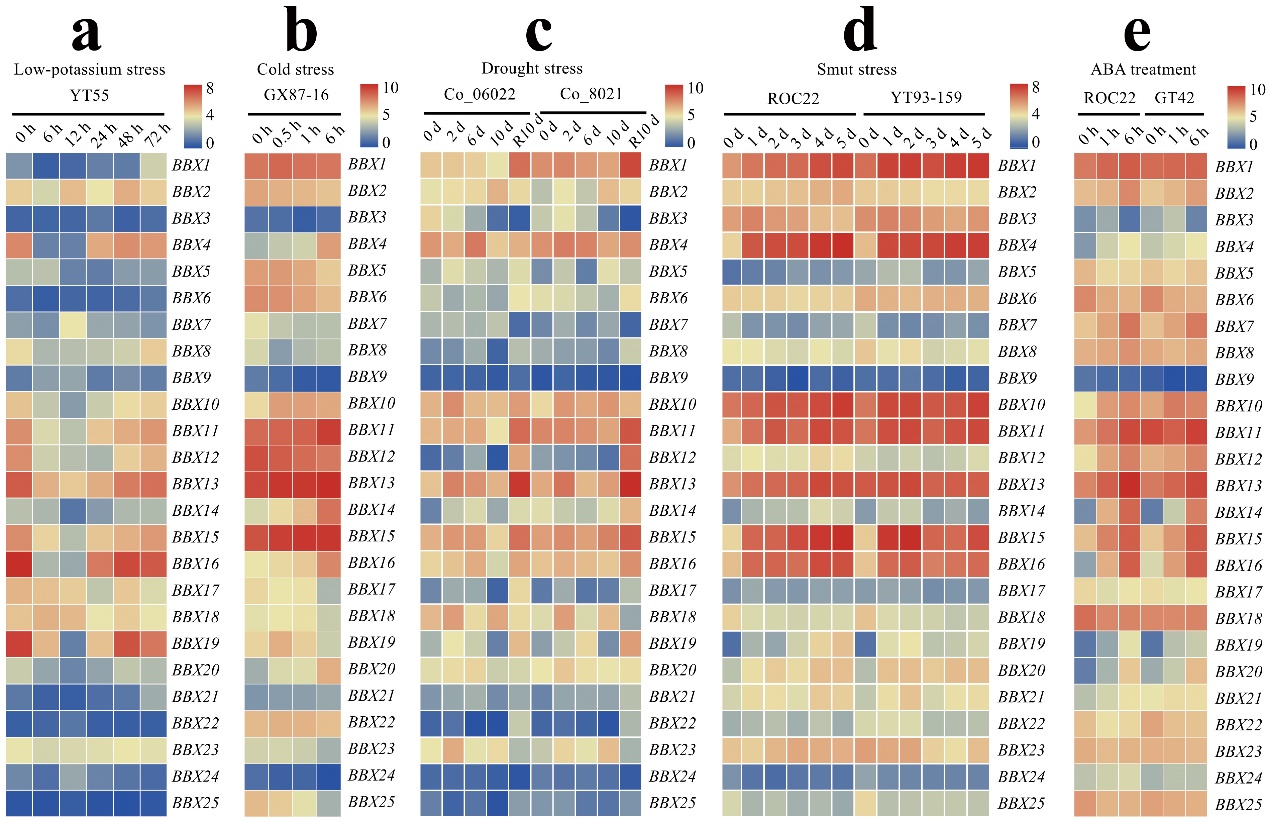
**Figure S3.** Expression patterns of the sugarcane *BBX* genes from the sugarcane transcriptome data in five different conditions. (a) Expression patterns of the *BBX* genes in the roots of *Saccharum* hybrid cultivar YT55 under low-potassium stress, and samples were collected at 0, 6, 12, 24, 48, and 72 h (PRJNA262715). (b) Expression patterns of the *BBX* genes in the leaves of *Saccharum* hybrid cultivar GX87-16 under cold stress, and samples were collected at 0, 0.5, 1, and 6 h (PRJNA636260). (c) Expression patterns of the *BBX* genes in the leaves of *Saccharum* hybrid cultivar Co_06022 (susceptible cultivar) and Co_8021 (resistant cultivar) after 0, 2, 6, and 10 d drought stress and recovery treatment (PRJNA590595). (d) Expression patterns of the *BBX* genes in the buds of *Saccharum* hybrid cultivar ROC22 (susceptible cultivar) and YC05-179 (resistant cultivar) after smut pathogen infection at 0, 1, 2, and 5 d (PRJCA011580). (e) Expression patterns of the *BBX* genes in the buds of *Saccharum* hybrid cultivar ROC22 (susceptible cultivar) and GT42 (resistant cultivar) after ABA treatment at 0, 1, and 6 h (PRJNA555450). The heat map was plotted with the TBTools software (v1.098), with the transcript level of the *BBX* genes transformed as log_2_ FPKM (fragments per kilobase million), ranging from blue (low expression level) to red (high expression level).
